# Genome-wide association scan for heterotic quantitative trait loci in multi-breed and crossbred beef cattle

**DOI:** 10.1186/s12711-018-0405-y

**Published:** 2018-10-05

**Authors:** Everestus C. Akanno, Liuhong Chen, Mohammed K. Abo-Ismail, John J. Crowley, Zhiquan Wang, Changxi Li, John A. Basarab, Michael D. MacNeil, Graham S. Plastow

**Affiliations:** 1 Livestock Gentec, Department of Agricultural, Food and Nutritional Science, University of Alberta, Edmonton, AB Canada; 2 Department of Animal and Poultry Production, Damanhour University, Damanhour, Egypt; 3 Canadian Beef Breeds Council, 6815 8th Street N.E., Calgary, AB Canada; 4 Lacombe Research and Development Centre, Agriculture and Agri-Food Canada, 6000 C & E Trail, Lacombe, AB Canada; 5 Alberta Agriculture and Forestry, 6000 C & E Trail, Lacombe, AB Canada; 6 Delta G, Miles City, MT USA; 7 Department of Animal, Wildlife and Grassland Sciences, University Free State, Bloemfontein, South Africa

## Abstract

**Background:**

Heterosis has been suggested to be caused by dominance effects. We performed a joint genome-wide association analysis (GWAS) using data from multi-breed and crossbred beef cattle to identify single nucleotide polymorphisms (SNPs) with significant dominance effects associated with variation in growth and carcass traits and to understand the mode of action of these associations.

**Methods:**

Illumina BovineSNP50 genotypes and phenotypes for 11 growth and carcass traits were available for 6796 multi-breed and crossbred beef cattle. After performing quality control, 42,610 SNPs and 6794 animals were used for further analyses. A single-SNP GWAS for the joint association of additive and dominance effects was conducted in purebred, crossbred, and combined datasets using the ASReml software. Genomic breed composition predicted from admixture analyses was included in the mixed effect model to account for possible population stratification and breed effects. A threshold of 10% genome-wide false discovery rate was applied to declare associations as significant. The significant SNPs with dominance association were mapped to their corresponding genes at 100 kb.

**Results:**

Seven SNPs with significant dominance associations were detected for birth weight, weaning weight, pre-weaning daily gain, yearling weight and marbling score across the three datasets at a false discovery rate of 10%. These SNPs were located on bovine chromosomes 1, 3, 4, 6 and 21 and mapped to six putative candidate genes: *U6atac*, *AGBL4*, *bta*-*mir*-*2888*-*1*, *REPIN1*, *ICA1* and *NXPH1*. These genes have interesting biological functions related to the regulation of gene expression, glucose and lipid metabolism and body fat mass. For most of the identified loci, we observed over-dominance association with the studied traits, such that the heterozygous individuals at any of these loci had greater genotypic values for the trait than either of the homozygous individuals.

**Conclusions:**

Our results revealed very few regions with significant dominance genetic effects across all the traits studied in the three datasets used. Regarding the SNPs that were detected with dominance associations, further investigation is needed to determine their relevance in crossbreeding programs assuming that dominance effects are the main cause of (or contribute usefully to) heterosis.

**Electronic supplementary material:**

The online version of this article (10.1186/s12711-018-0405-y) contains supplementary material, which is available to authorized users.

## Background

Genome-wide association studies (GWAS) offer the opportunity to use available genotypes in the form of single nucleotide polymorphisms (SNPs) such as the Illumina BovineSNP50 BeadChip (50K; Illumina Inc., San Diego, CA) to identify genomic regions that are associated with phenotypic variation in economically important traits in cattle [1, 2]. For several beef cattle traits, including feed efficiency, growth, carcass, and reproduction, the number of SNPs involved in their genetic variation and mapped to putative quantitative trait loci (QTL) has rapidly expanded [3–5]. However, the genetic effects of the underlying QTL, which are captured by neighbouring SNPs in linkage disequilibrium (LD), are generally modelled as additive effects [6]. The assumption of additivity in genetic evaluation models is made because the goal is to estimate breeding values for selection purposes. In reality, both additive and non-additive gene effects contribute to the total genetic variance for a given quantitative trait [7, 8]. One possibility to better understand and clarify the inheritance of complex traits is to decipher the contributions of non-additive gene effects including within-locus (dominance) and between-loci (epistasis) interactions.

Few studies have investigated the importance of non-additive gene effects and these concluded that accounting for these effects in animal genetic evaluation models can improve genomic prediction in comparison to additive models [9–13]. In beef cattle, thanks to the availability of genomic tools, attempts to estimate the proportion of the total phenotypic variation that is attributed to non-additive genetic effects for those traits that express heterosis [13, 14] have been made. For example, Bolormaa et al. [14] estimated that the proportion of the variance explained by dominance ranged from 0 to 42% for growth, carcass, and fertility traits in beef cattle. In an earlier study, Akanno et al. [13] reported estimates of the proportion of variance explained by dominance from 0 to 9% for growth and carcass traits in beef cattle. Both studies suggest that non-additive genetic effects may contribute to variation in beef cattle traits, which may be explained by specific loci across the bovine genome. Nevertheless, none of these studies evaluated the mode of inheritance and the magnitude of the non-additive effects across the genome. Here, we hypothesised that the non-additive genetic effects may be due to QTL with dominance, over-dominance or epistatic interactions, which are the suggested genetic mechanisms that underlie heterosis [7, 9–15].

Therefore, the objective of our study was to identify genomic regions or SNPs with simultaneously additive and non-additive effects that are associated with growth and carcass traits in beef cattle and to understand the mode of action of these associations.

## Methods

### Animals and phenotypes

Data from 6796 multi-breed and crossbred beef cattle born between 1998 and 2012 were collated from various projects and research herds across Canada including: 3692 from the Phenomic Gap Project based at Lacombe Research Centre; 2350 from the University of Alberta’s Roy Berg Kinsella Research Ranch; and 754 from the University of Guelph’s Elora Beef Cattle Research Station. The population structure, breeds, and animal management were previously described in detail by Lu et al. [16]. Briefly, the whole dataset consisted of 968 Angus, 572 Charolais, 316 Hereford, 17 Simmental, 17 Limousine, 1225 Angus-Hereford crossbred, 484 Angus-Simmental crossbred, 353 Charolais-Red Angus crossbred, 1178 Kinsella composite, 1105 Beefbooster TX composite, and 561 animals of other breed combinations. Kinsella composite is a dual-purpose hybrid that is strongly influenced by approximately 50% Hereford and 30% Angus breeds with 20% infusion of Holstein [17]. Beefbooster TX composite is predominantly Charolais-based (approximately 60%) with 40% infusion of other breeds including Holstein, Maine Anjou, and Chianina (http://www.beefbooster.com).

Phenotypic records including birth weight (BWT), weaning weight (WWT), pre-weaning daily gain (PDG), average daily gain on feedlot (ADG), yearling weight (YWT), hot carcass weight (HCW), back fat thickness (FAT), rib eye area (REA), marbling score (MBS), lean meat yield (LMY) and yield grade (YG) were available. Yield grade was calculated according to the United States Department of Agriculture (USDA) specification [18]. The data were edited to remove records with more or less 3 standard deviations (SD) from the mean after correcting for systematic effects of sex, age of dam, data source, herd and year of birth. See Table 1 for details of number of animals with records, trait means and standard deviations. Pedigree data extending to purebred ancestors was available for all animals used in the study and assumed to be accurate. Pedigree records consisted of 11,905 individuals including 873 sires and 4483 dams across five generations.

**Table 1:** Number of animals with a record (N), mean and standard deviation (SD) for growth and carcass traits of multi-breed and crossbred beef cattle

Traits	N	Mean	SD
Birth weight (kg)	5481	41.87	6.69
Weaning weight (kg)	6261	239.33	44.60
Pre-weaning daily gain (kg/d)	5255	1.13	0.17
Average daily gain (kg/d)	6772	1.45	0.39
Yearling weight (kg)	6019	366.91	66.92
Hot carcass weight (kg)	4071	335.87	34.26
Back fat thickness (mm)	4002	11.19	4.50
Rib eye area (cm^2^)	4054	85.61	11.18
Marbling score	4054	406.19	94.55
Lean meat yield (%)	4062	58.37	4.57
Yield grade	4008	2.66	0.81

### Genotyping, quality control and genomic breed fractions

All animals with phenotype records were genotyped with the 50K SNP panel at Delta Genomics, Edmonton, AB, Canada. Quality control was performed to remove SNPs with a minor allele frequency (MAF) lower than 0.01, a call rate higher than 0.90 and that deviated significantly (*p* < 0.05) from Hardy–Weinberg equilibrium [16]. Missing genotypes were imputed using FImpute v2.0 [19]. In addition, two animals with a call rate lower than 90% were also removed, and only autosomal SNPs with a known genome position according to the UMD_3.1 bovine assembly map [20] were used. After editing, 42,610 SNPs and 6794 animals were used for the GWAS.

Genomic breed fractions were predicted for all individuals using the ADMIXTURE software [21]. A ten-fold cross-validation procedure available in ADMIXTURE was performed to find the best possible *K* value with the smallest cross-validation error [21], where *K* is the number of postulated ancestral populations. The resulting breed fractions at *K* = 6 identified six breed ancestries in the dataset including Angus, Hereford, Charolais, Kinsella Composite, Beefbooster TX Composite, and two- or three-way crossbreds. See Fig. 1 in Akanno et al. [13] for the distribution of estimated genomic breed fractions in the whole dataset. The genomic breed fraction was used to designate animals as purebreds (n = 1467) based on having Angus, Hereford or Charolais breed fractions greater than 80% while the rest were designated as crossbreds (n = 5327). The same MAF threshold as that applied to the whole dataset was used to filter SNPs in the purebred and crossbred groups, which resulted in 42,270 and 42,536 SNPs, respectively, and these were used for GWAS in these two groups.

**Fig. 1:**
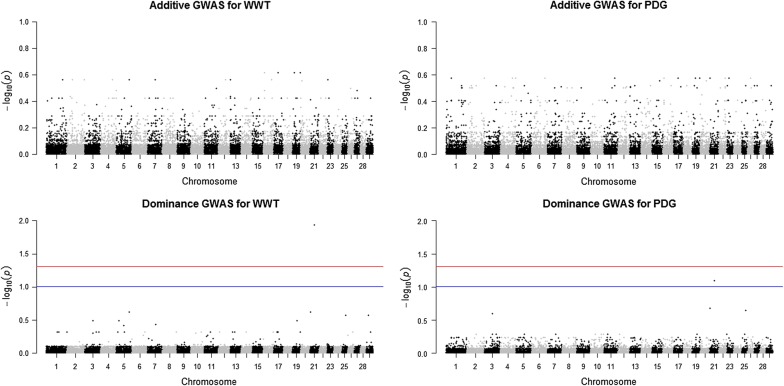
Joint genome-wide association of additive and dominance SNP effects for weaning weight (WWT; left) and pre-weaning daily gain (PDG; right) evaluated in purebreds (n = 1467). The purebred group included individuals with more than 80% of Angus, Hereford and Charolais breed proportions, respectively. Significant SNPs were determined with a false discovery rate correction at 5% (red line) and 10% (blue line)

### Statistical analyses

In an earlier study [13], assuming that heterosis is due to dominance and over-dominance, we investigated the contribution of additive and dominance effects to the total phenotypic variation in purebred, crossbred, and combined data, which underpins the motivation for the current study. Here, a single-SNP GWAS for the joint association of additive and dominance effects was performed on the studied traits in purebred, crossbred and combined data using the ASReml software [22] based on the following linear mixed effect model:

1 $${\mathbf{y}} = {\mathbf{1}}\mu + {\mathbf{Xb}} + {\mathbf{Za}} + {\mathbf{v}}\alpha + {\mathbf{w}}\delta + {\mathbf{e}},$$

where ${\mathbf{y}}$ is a vector of phenotypic observation; $\mu$ is the population mean and ${\mathbf{1}}$ is a vector of ones; depending on the trait analysed, ${\mathbf{X}}$ is the design matrix that relates the fixed effects to the observation and ${\mathbf{b}}$ is a vector of fixed effects including linear covariates of dam age, weaning age, start age for feedlot test and genomic breed fractions, data source and contemporary groups based on herd, year, sex, and management groups. The genomic breed fractions were used for correction of possible population stratification and breed effects. ${\mathbf{Z}}$ is a design matrix relating observations to random animal genetic effects; ${\mathbf{a}}$ is a vector of random additive polygenic effects that is assumed to be normally distributed as: ${\mathbf{a}}\sim{\text{N}}\left( {{\mathbf{0}},\sigma_{{\mathbf{a}}}^{2} {\mathbf{A}}} \right)$, where $\sigma_{{\mathbf{a}}}^{2}$ is the additive genetic variance and ${\mathbf{A}}$ is the additive relationship matrix constructed from pedigree data; ${\mathbf{v}}$ is a vector of SNP genotypes, coded as 0, 1, and 2 for the number of a particular allele at the SNP; $\alpha$ is the allele substitution (additive) effect; ${\mathbf{w}}$ is a incidence vector of dominance coded as 1 for the heterozygous genotype (*AB*) and 0 for the two homozygous genotypes (*AA* and *BB*) for each SNP; $\delta$ is the dominance effect; ${\mathbf{e}}$ is a vector of random residual effects that is assumed to be normally distributed as ${\mathbf{e}}\sim{\text{N}}\left( {{\mathbf{0}},\sigma_{{\mathbf{e}}}^{2} {\mathbf{I}}} \right)$, with ${\mathbf{I}}$ being an identity matrix. Vectors ${\mathbf{v}}$ and ${\mathbf{w}}$ were fitted as covariates. Random maternal genetic and permanent environmental effects were also fitted in the model for the analysis of pre-weaning traits (BWT, WWT and PDG). The GWAS model was parameterised to be able to test the significance of additive and dominance effects simultaneously at each SNP using the Wald F statistics available in the Asreml software [22].

As noted by Falconer and Mackay [7], epistasis without the presence of dominance cannot cause heterosis by itself. Therefore, each of the SNPs with significant dominance effects identified in either the purebred, crossbred or combined data were tested for pair-wise epistatic interaction with the remaining SNPs across the genome using Model (2) and evaluated within the dataset in which the SNP was identified:

2 $${\mathbf{y}} = {\mathbf{1}}\mu + {\mathbf{Xb}} + {\mathbf{Za}} + {\mathbf{s}}\beta + {\mathbf{v}}_{{ - {\mathbf{s}}}} \alpha + \left( {{\mathbf{s}} \times {\mathbf{v}}_{{ - {\mathbf{s}}}} } \right)m + {\mathbf{e}},$$

where ${\mathbf{y}}$, $\mu$, ${\mathbf{1}}$, ${\mathbf{X}}$, ${\mathbf{b}}$, ${\mathbf{Z}}$, ${\mathbf{a}}$ and ${\mathbf{e}}$ are the same as in Model (1); ${\mathbf{s}}$ is a vector of the coded genotypes for one leading SNP with a significant dominant association; $\beta$ is the allele substitution effect of the leading SNP; ${\mathbf{v}}_{{ - {\mathbf{s}}}}$ is the vector of coded genotypes for one of the remaining SNPs across the genome; $\alpha$ is the allele substitution effect as above; (${\mathbf{s}} \times {\mathbf{v}}_{{ - {\mathbf{s}}}}$) is a vector of element-wise multiplication of genotype codes representing the additive $\times$ additive interaction between one leading SNP and one of the remaining SNPs; and $m$ is the epistatic interaction effects.

### Multiple-testing corrections

The false discovery rate (FDR) [23] implemented in the R package GenABEL [24] was used to correct for multiple-testing. A maximum threshold of 10% for the genome-wide FDR was used to control for false positives and to declare associations as significant additive and dominance effects. The quantile–quantile (Q–Q) plots of the *p* values for each SNP were used to compare observed distributions of − log (*p* value) to the expected distribution under the null hypothesis for each trait. The Manhattan plots of *p* values for each SNP were also used to illustrate significant associations at the level of each chromosome and trait. All plots were completed using the R package qqman [25].

### Mapping of candidate genes

The SNPs with a significant dominance association identified from the GWAS analyses were mapped to their corresponding genes or near to the genes, i.e. at 100 kilo base pairs (kbp) on either side using NGS-SNP [26], based on the National Center for Biotechnology Information (NCBI) [27] and Ensembl Genome Browser [28] databanks. The 100-kbp window was chosen because the average LD ($r^{2}$) between pairs of syntenic SNPs within this distance was around 0.20 in a related beef cattle population [29].

### Estimation of genotypic effects

To determine the mode of action of SNPs with significant dominance effects, genotype effects were estimated according to Model (3):

3 $${\mathbf{y}} = {\mathbf{1}}\mu + {\mathbf{Xb}} + {\mathbf{SNP}} + {\mathbf{e}},$$

where ${\mathbf{y}}$, $\mu$, ${\mathbf{1}}$, ${\mathbf{X}}$, ${\mathbf{b}}$ and ${\mathbf{e}}$ are the same as in Models (1) and (2); ${\mathbf{SNP}}$ is a vector of genotype class *AA*, *AB* and *BB*, i.e. the SNP genotype was fitted as a classification factor. The least square means of each genotypic class was determined and plotted to characterise the mode of action for significant associations with the traits of interest. Analyses were conducted in R statistical software using default package where applicable [30].

## Results

The number of significant SNPs with additive and dominance effects that were identified for growth and carcass traits in the purebred, crossbred and combined populations of beef cattle is in Table 2. At a FDR of 10%, 14, 294 and 369 significant additive associations were identified, while only 2, 3 and 4 significant dominant associations were observed in purebred, crossbred and combined data, respectively (Table 2). For both additive and dominance associations, the number of unique and significant SNPs identified was larger when using combined data than crossbred data and was much smaller with purebred data for all studied traits (Table 3) and (see Additional file 1: Tables S1, S2 and S3). A representation of the Q–Q plots of the observed *p* values showed departures from the expected distribution under the null hypothesis of polygenic variation (see Additional file 2: Figures S1, S2, S3, S4, S5, S6, S7, S8, S9, S10, S11 and S12) and the Manhattan plots that show the significant peaks for additive and dominance association for all studied traits and across the three datasets are in Figures S13 to S24 (see Additional file 2: Figures S13, S14, S15, S16, S17, S18, S19, S20, S21, S22, S23 and S24).

**Table 2:** Number of additive and dominance significant SNPs jointly identified at a false discovery rate of 5 and 10% for the studied traits in purebreds, crossbreds and combined populations of beef cattle using the Illumina BovineSNP50 BeadChip

Traits	Purebreds (n = 1467)				Crossbreds (n = 5327)				Combined (n = 6794)			
	Additive		Dominance		Additive		Dominance		Additive		Dominance	
	5%	10%	5%	10%	5%	10%	5%	10%	5%	10%	5%	10%
Birth weight (kg)	5	7	0	0	58	109	1	2	66	116	0	0
Weaning weight (kg)	0	0	0	1	10	21	0	0	18	24	1	1
Pre-weaning daily gain (kg/d)	0	0	0	1	8	10	0	0	2	3	0	1
Average daily gain (kg/d)	1	1	0	0	23	40	0	0	37	49	0	0
Yearling weight (kg)	0	0	0	0	50	71	0	1	64	94	0	0
Hot carcass weight (kg)	0	1	0	0	17	23	0	0	24	29	0	0
Back fat thickness (mm)	0	0	0	0	3	6	0	0	6	8	0	0
Rib eye area (cm^2^)	0	3	0	0	12	12	0	0	15	19	0	0
Marbling score	0	0	0	0	0	2	0	0	3	4	2	2
Lean meat yield (%)	0	1	0	0	0	0	0	0	7	14	0	0
Yield grade	1	1	0	0	0	0	0	0	0	9	0	0

Purebred individuals have > 80% of Angus, Hereford and Charolais; crossbred individuals included Kinsella composite, Beefbooster TX composite (www.beefbooster.com) and two and more way crosses involving Angus, Hereford, Charolais, Gelbvieh, Simmental, Limousin, and Piedmontese breeds

**Table 3:** Identity, position, and effects of significantly associated dominance SNPs obtained by single SNP regression mixed model for birth weight (BWT), weaning weight (WWT), pre-weaning daily gain (PDG), yearling weight (YWT) and marbling score (MBS) in beef cattle

Traits	Groups	SNP reference	BTA	Position	MAF	*p* value	FDR (%)	Allele substitution effects	Dominance effects	Genes	Region
BWT (kg)	C	rs110704582	1	113,215,525	0.327	4.60e−07	5	0.404	− 0.942	*U6atac*	Intergenic
	C	rs41596755	6	55,697,300	0.244	3.91e−06	10	0.552	1.012	–	Intergenic
WWT (kg)	P	rs42779004	21	36,186,103	0.196	2.74e−07	5	− 4.016	6.994	*bta*-*mir*-*2888*-*1*	Intergenic
	A	rs29027109	3	98,222,548	0.387	2.64e−07	5	− 0.361	2.610	*AGBL4*	Intron
PDG (kg/d)	P	rs42779004	21	36,186,103	0.196	1.89e−06	10	− 0.019	0.037	*bta*-*mir*-*2888*-*1*	Intergenic
	A	rs29027109	3	98,222,548	0.387	2.29e−06	10	− 0.001	0.016	*AGBL4*	Intron
YWT (kg)	C	rs109808526	4	113,614,764	0.318	1.18e−06	10	− 1.562	6.316	*REPIN1*	Intergenic
MBS	A	rs110361335	4	17,230,513	0.182	6.47e−07	5	8.317	13.88	*ICA1*	Intron
	A	rs110564527	4	17,657,399	0.207	1.20e−07	5	− 8.023	15.37	*NXPH1*	Intergenic

P = purebred sample included individuals with > 80% of Angus, Hereford and Charolais (n = 1467); C = crossbred sample included Kinsella composite, Beefbooster TX composite (www.beefbooster.com) and two and more way crosses involving Angus, Hereford, Charolais, Gelbvieh, Simmental, Limousin, and Piedmontese breeds (n = 5327); A = combined data of all individuals in the study (n = 6794)

**Fig. 2:**
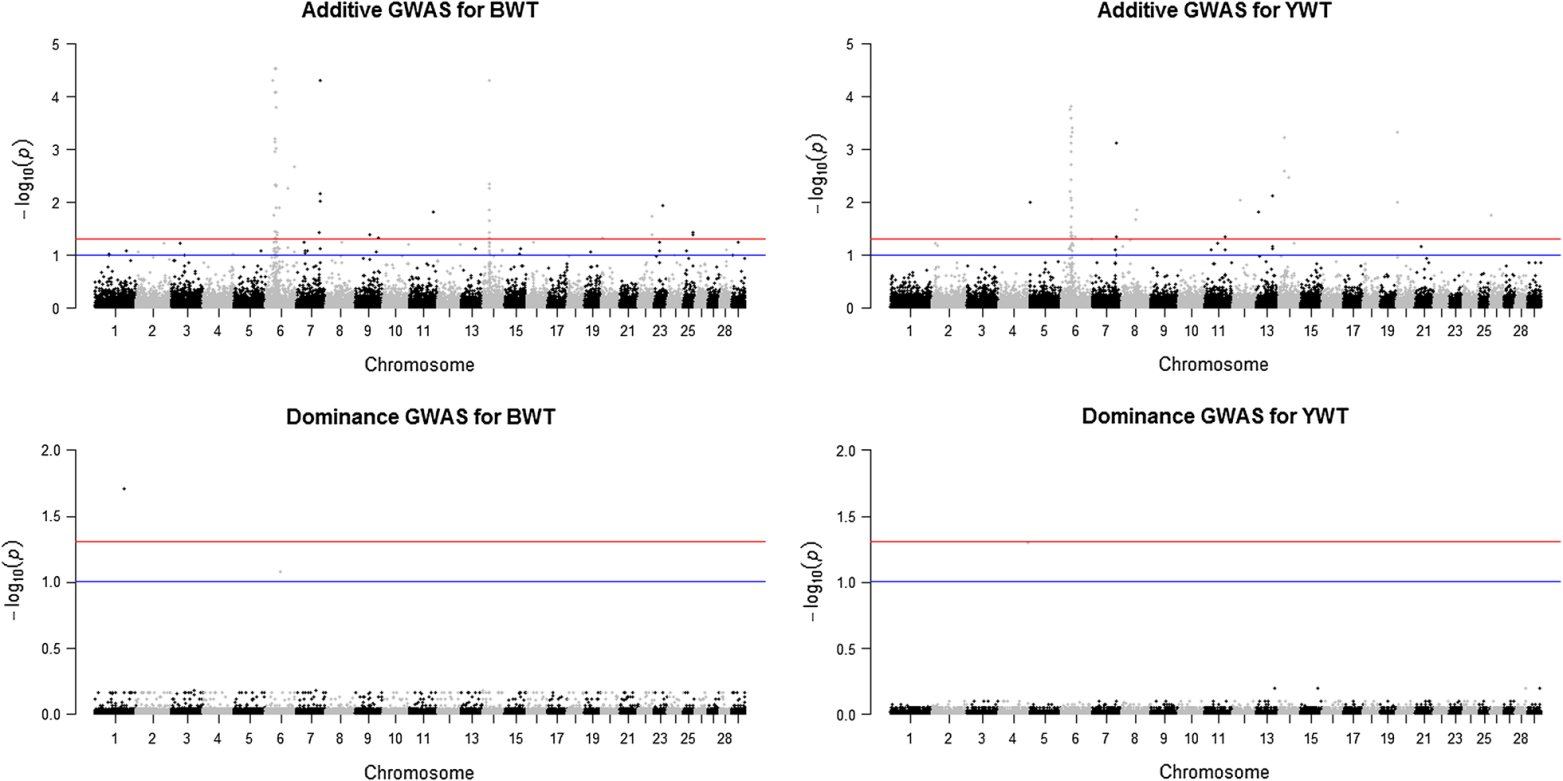
Joint genome-wide association of additive and dominance SNP effects for birth weight (BWT; left) and yearling weight (YWT; right) evaluated in crossbreds (n = 5327). The crossbred group included Kinsella composite, Beefbooster TX composite (www.beefbooster.com) and two and more way crosses involving Angus, Hereford, Charolais, Gelbvieh, Simmental, Limousin, and Piedmontese breeds. Significant SNPs were determined with a false discovery rate correction at 5% (red line) and 10% (blue line)

**Fig. 3:**
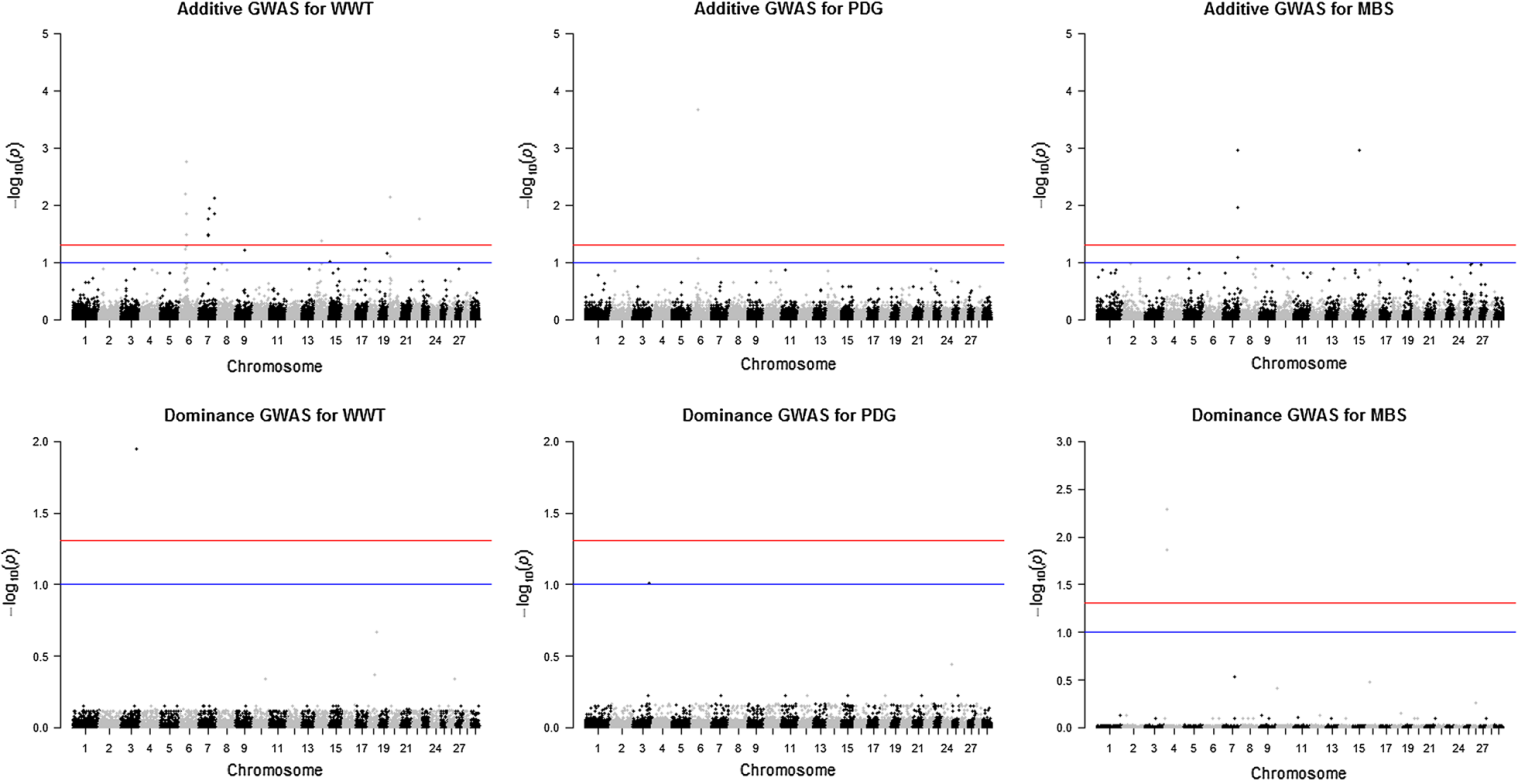
Joint genome-wide association of additive and dominance SNP effects for weaning weight (WWT; left), pre-weaning daily gain (PDG; center) and marbling score (MBS; right) evaluated in combined data (n = 6794). Significant SNPs were determined with a false discovery rate correction at 5% (red line) and 10% (blue line)

Seven SNPs showed significant dominance effects for BWT, WWT, PDG, YWT and MBS across the three datasets at a FDR of 5 and 10% (Table 3 and Figs. 1, 2, 3). SNPs rs42779004, rs110704582, rs41596755, rs1090808526, rs29027109, rs110361335 and rs110564527 were identified in the purebred, crossbred and combined data. Two SNPs, rs110704582 and rs41596755 on BTA1 and 6 (BTA for *Bos taurus* chromosome), respectively, were associated with BWT. For WWT and PDG, two SNPs rs29027109 and rs42779004 on BTA3 and 21 showed significant pleiotropic dominance association for both traits. One SNP, rs109808526 on BTA4 was associated with YWT, while two SNPs, rs110361335 and rs110564527 on BTA4 were associated with MBS (Table 3). The estimated effect of the minor allele ranged from 0.404 to 0.552 kg for BWT, from − 4.016 to − 0.361 kg for WWT, from − 0.019 to − 0.001 kg/d for PDG, − 1.562 kg for YWT and from − 8.023 to 8.315 for MBS, while dominance effects ranged from − 0.942 to 1.012 kg for BWT, from 2.610 to 6.994 kg for WWT, from 0.016 to 0.037 kg/d for PDG, 6.316 kg for YWT and from 13.88 to 15.37 for MBS (Table 3). Six genes were mapped as putative candidates that underlie these associations (Table 3). Two of the heterotic SNPs were located within an intron of the candidate genes, while four SNPs were in intergenic regions, and one SNP was not mapped to any known candidate gene (Table 3).

**Fig. 4:**
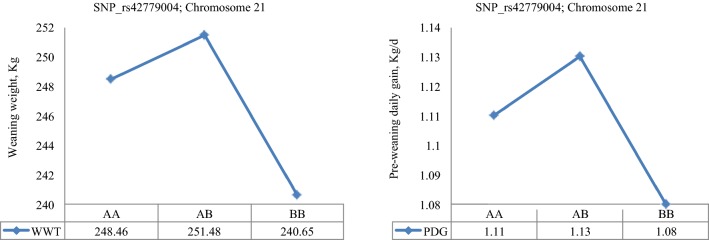
Least square means for the genotypic class of significant heterotic SNPs on BTA21 associated with weaning weight and pre-weaning daily gain in purebreds. Purebred group included individuals with more than 80% of Angus, Hereford and Charolais breed proportions, respectively

**Fig. 5:**
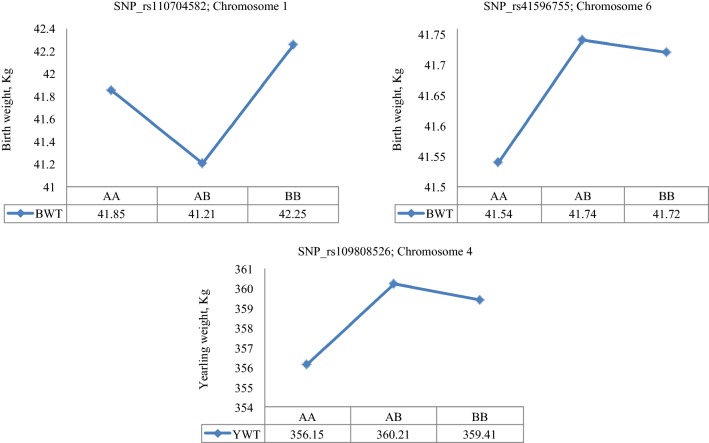
Least square means for the genotypic class of significant heterotic SNPs on BTA1, 6 and 4 associated with birth weight and yearling weight in crossbreds. The crossbred group included Kinsella composite, Beefbooster TX composite (www.beefbooster.com) and two-way or more crosses involving Angus, Hereford, Charolais, Gelbvieh, Simmental, Limousin, and Piedmontese breeds

**Fig. 6:**
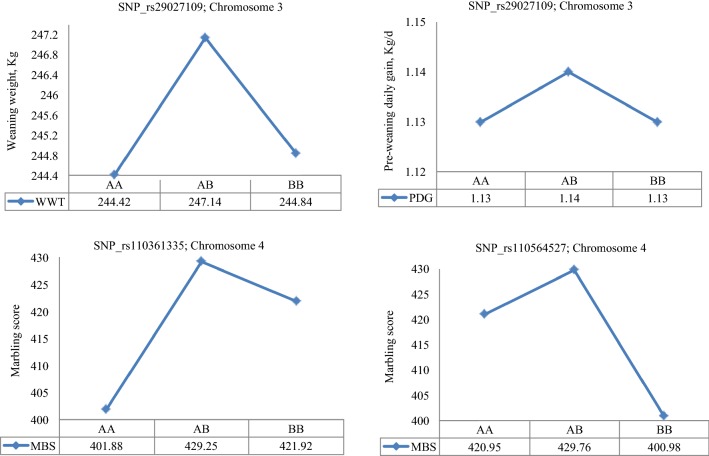
Least square means for the genotypic class of significant heterotic SNPs on BTA3 and 4 associated with weaning weight, pre-weaning daily gain and carcass marbling score in combined data

The least square means of the genotypic classes are in Figs. 4, 5, 6. SNP rs42779004, which was identified in the purebred data showed characteristics of over-dominance association with WWT and PDG, with genotypic values for the heterozygous individuals (*AB*) being significantly (*p* < 0.01) higher than those for either of the two homozygotes (*AA* and *BB*) for both traits (Fig. 4). SNPs rs41596755 and rs109808526, which were identified in the crossbred data, showed over-dominance association with BWT and YWT, respectively, while SNP rs110704582 showed characteristics of under-dominance for BWT since the heterozygotes had a lower birth weight than either of the homozygotes (Fig. 5). The remaining SNPs rs29027109, rs110361335 and rs110564527 detected in the combined data exhibited over-dominance association with WWT, PDG and MBS because the least square means of heterozygotes were significantly (*p* < 0.05) higher than those of either of the homozygotes across the three traits (Fig. 6).

**Fig. 7:**
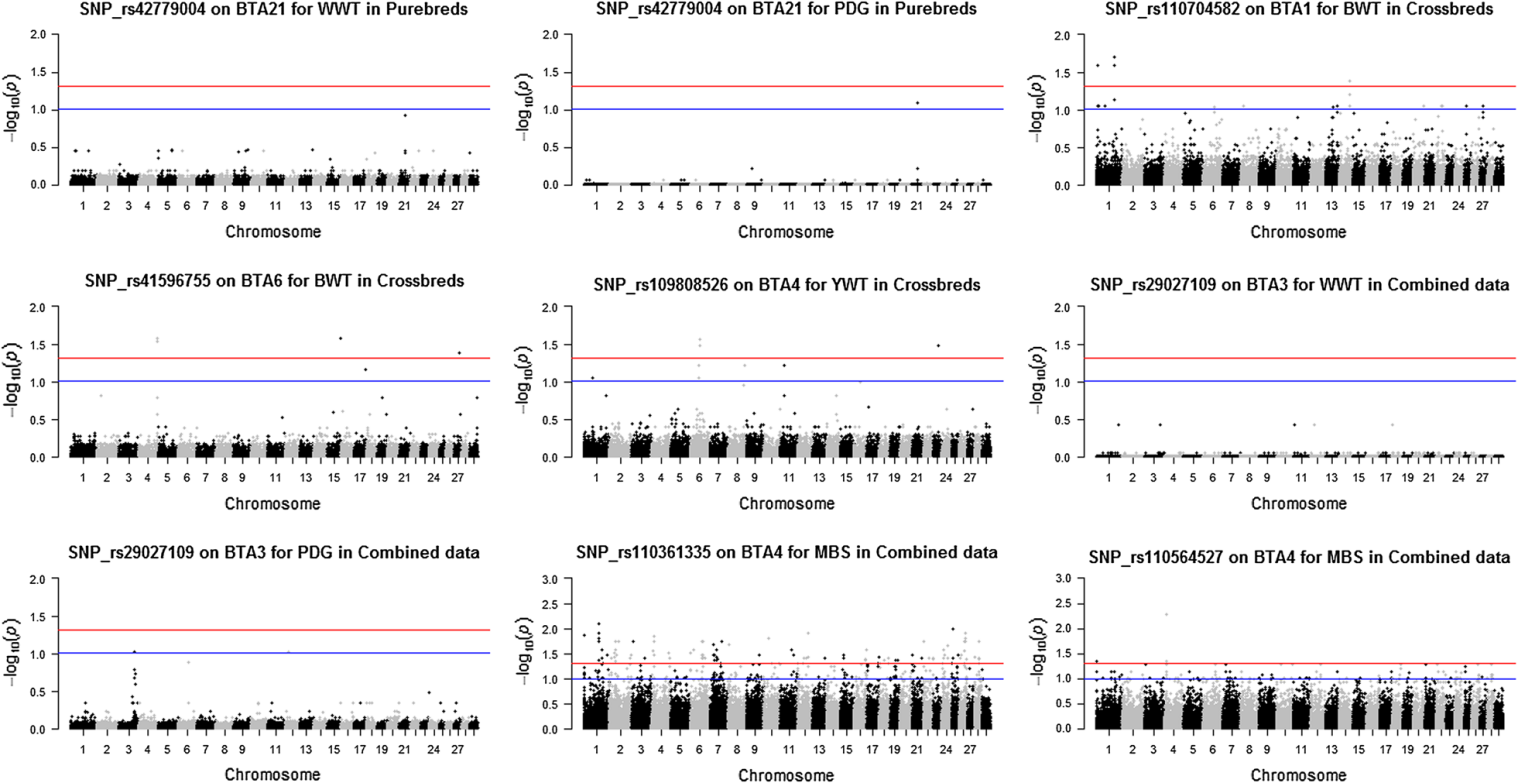
Pair-wise epistatic interaction between the leading significant dominant SNPs and the rest of the SNPs across the genome evaluated in purebred, crossbred and combined data. Only additive x additive interaction was tested. Significant interactions were determined by false discovery rate correction at 5% (red line) and 10% (blue line)

The numbers of additive × additive epistatic interactions between the seven leading significant dominant SNPs and the rest of the SNPs across the genome were examined for BWT, WWT PDG, YWT and MBS in the three datasets (Fig. 7). At least one significant epistatic interaction was identified at a FDR of 10% for all leading SNPs (Fig. 7). However, for MBS, 290 significant epistatic interactions were found between the leading SNP rs110361335 (within the *ICA1* gene) and the other SNPs. Similarly, the numbers of significant epistatic interactions between SNPs rs110564527, rs110704582, rs109808526, rs41596755, rs29027109 and rs42779004 with the other SNPs at a FDR of 10% were equal to 111, 19, 11, 6, 2 and 1 for MBS, BWT, YWT, BWT, PDG and PDG, respectively. Unfortunately, none of the identified SNPs showed a significant epistatic interaction for WWT.

## Discussion

### Heterotic QTL

Most of the studies on the genetic evaluation of beef cattle traits using genomic information have focused on the discovery or use of additive genetic effects [3–5, 31, 32] because these genetic effects are passed from parents to offspring and are the basis of genetic selection and improvement programs. In the current study, many QTL regions with genome-wide significance for additive genetic effects were identified [see Additional file 1: Tables S1, S2 and S3]. For most of the traits studied, peaks for significant additive SNPs across the three datasets were on BTA6 followed by BTA7 and 14 (see Additional file 2: Figures S13, S14, S15, S16, S17 and S18), which correspond to previously identified QTL [3, 33–36]. These additive SNPs can contribute to the process of building consensus beef cattle QTL effects and can also provide a starting point for mapping the underlying candidate genes. However, the motivation here was to identify and characterise QTL that are attributed to heterosis assuming a dominance model. Our results showed very few regions with evidence of significant dominance effects across all the traits studied in the three datasets used [see Additional file 2: Figures S18, S19, S20, S21, S22, S23 and S24]. The possible signals detected were associated with BWT, WWT, PDG, YWT and MBS (Table 3) and peaks for significant dominance SNPs were on BTA1, 3, 4, 6 and 21 (Figs. 1, 2, 3).

The ability to identify QTL with large effects on any trait depends partly on the amount of trait variation that can be attributed to the different genetic sources. For example, in beef cattle, growth and carcass traits are moderately to highly heritable [13], which results in the identification of several QTL with additive effects (see Additional file 1: Tables S1, S2 and S3). In the case of dominance and over-dominance, the percentage of phenotypic variation due to non-additive genetic effects for growth and carcass traits is small [9, 12–14], which suggests that fewer dominance QTL may be identified. The results of our study indicate a lack of power in detecting heterotic QTL, which is also reflected by an even smaller proportion of dominance SNPs observed for the traits studied in an earlier study [13]. Furthermore, the rather small number of SNPs with significant dominance effects may be related to errors introduced by inconsistent LD across multiple populations [37] and to the assumption that QTL effects are the same across multiple breeds. Nevertheless, our study demonstrates that dominance genetic effects may be polygenic (i.e. explained by multiple regions all with a small effect) for most growth and carcass traits of beef cattle.

Most of the heterotic QTL identified in this study were associated with growth traits including birth weight, weaning weight, pre-weaning daily gain and yearling weight, which are known to express heterosis [38–41]. Although, the genetic basis of heterosis is still a subject of scientific investigation, few studies have shown that dominance is an important factor contributing to heterosis [13, 15, 41–45], whereas epistasis has been implicated in other studies [14, 46, 47]. As noted by Falconer and Mackay [7], epistasis without the presence of dominance cannot cause heterosis by itself. Moreover, the power to estimate epistatic effects in segregating populations [46] such as beef cattle populations may be low. Here, several peaks of significant epistatic interactions were associated with MBS followed by BWT, YWT and PDG (Fig. 7) but none with WWT. In a similar approach, Bolormaa et al. [14] observed a number of significant epistatic interactions for several beef cattle traits using 28 previously identified SNPs with additive effects. Therefore, epistatic interactions may have a role in the non-additive genetic variation of beef cattle traits, but this warrants further investigation.

### Trait association, candidate genes and mode of inheritance

Growth traits such as birth weight, weaning weight, pre-weaning daily gain and yearling weight are economically important traits in beef cattle, which are traditionally included in the selection criteria of beef cattle breeding programs [48] because they are moderately to highly heritable [13] and are genetically correlated to carcass and meat quality traits [49]. Several QTL that underlie the variation of growth traits have been detected in several GWAS on different beef cattle populations (Table 2) [5, 32, 34–36]. SNPS were generally mapped to nearly all of the chromosomes, except BTA3, 9, 10 12, 13, 19, 20 23, 24 and 26 [34–36] and were linked to several candidate genes that differed from those detected in the current study. These previous studies mainly considered additivity when evaluating effects of SNP genotypes. However, growth traits are known to express heterotic advantage [39–41], which suggests the implication of non-additive genetic effects, in particular dominance. To date, there is no record of an attempt to detect heterotic QTL for growth traits and to characterise the nature of this association.

In this study, we identified two SNPs with significant dominance effects: rs110704582 and rs41596755 for BWT on BTA1 and 6, respectively. SNP rs110704582 is located in the intergenic region near the candidate gene *U6atac*, which is a known non-coding RNA involved in mRNA splicing, while SNP rs41596755 was not associated with any candidate gene. For WWT and PDG, two SNPs rs29027109 and rs42779004 on BTA3 and 21, respectively, were found to exhibit a pleiotropic effect on both traits, which is very likely due to the relationship between WWT and computation of PDG. SNP rs29027109 is located within an intron of the *AGBL4* gene, which encodes the ATP/GTP binding protein-like 4 [27]. This gene is not characterized in cattle. SNP rs42779004 is located in the intragenic region close to the gene *bta*-*mir*-*2888*-*1*, which encodes the microRNA 2888-1. This microRNA is involved in post-transcriptional regulation of gene expression in multicellular organisms by affecting both the stability and translation of mRNAs [27]. In addition, for YWT, SNP rs109808526 on BTA4 was located in the intergenic region of the gene *REPIN1* at about 11 kbp. It encodes the replication initiator 1 protein, a zinc finger protein that plays a role in insulin sensitivity, body fat mass and lipid metabolism by regulating the expression of key genes associated with glucose and lipid metabolism [50].

As a further step, the modes of action of SNPs with significant dominance associations with growth traits were evaluated across the three datasets. In the purebred data, SNP rs42779004 showed characteristics of over-dominance association with WWT and PDG (Fig. 4), which resulted in heterozygous calves at this locus having an earlier growth and a higher weight at weaning (251 kg) than either of the homozygous ones (241–248 kg). Unfortunately, this association was not observed in the crossbred or combined data. In the crossbred data, SNP rs110704582 exhibited under-dominance association with BWT (Fig. 5) such that heterozygous calves at this locus had a lower BWT (41 kg) than either of the homozygous ones (42 kg). Consequently, a negative heterosis for BWT may be expected in crossbreds, assuming that dominance effects are the main cause of heterosis. This SNP may be useful if calves with a lower BWT are desired in order to decrease the incidence of dystocia. In addition, two SNPs rs41596755 and rs109808526 showed characteristics of over-dominance association with BWT and YWT in crossbreds (Fig. 5) such that heterozygous individuals at these SNPs had a higher birth weight (43.7 kg) and yearling weight (360 kg) than either of the homozygous ones (41.5–42.7 kg for BWT and 356–359 kg for YWT). Therefore, depending on the breeding objective, selection at these loci may be useful for crossbreeding purposes in order to exploit hybrid vigour. In the combined data, one SNP rs29027109 also showed significant over-dominance association with WWT and PDG (Fig. 6) such that heterozygous calves had an early growth and a higher weight at weaning (247 kg) than the homozygous ones (244–245 kg).

Carcass marbling score (MBS) is an objective assessment of flecks of intramuscular fat evaluated at the 12th and 13th rib interface of the *longissimus* muscle and is associated with the tenderness, flavor and juiciness of beef [51]. The greater the amount of marbling, the higher the quality grade of beef carcass. This trait is moderately to highly heritable and genetically correlated with most carcass traits [4, 13]. In a review by Williams et al. [39], positive heterotic effects were observed for crosses involving taurine and indicine cattle breeds. Genome-wide association analyses for MBS identified a few additive SNPs on BTA3, 5, 15, 16, 18 and 25, that were associated with genes related to muscle development and lipid metabolism [4, 52, 53]. In our study, we found two SNPs, rs110361335 and rs110564527, that showed over-dominance associations with MBS in the combined data such that heterozygous individuals at both loci had more carcass marbling than the homozygous ones (Fig. 6). SNP rs110361335 on BTA4 is located within an intron of the *islet cell autoantigen 1* (*ICA1*) gene, which is known to be associated with glucose regulation and type 1 diabetes in humans [54], while SNP rs110564527 is located in the region near the candidate gene *neurexophilin 1 (NXPH1)*, which has no known association or characterization in cattle.

Furthermore, several peaks of epistatic interactions were observed between SNPs rs110361335 and rs110564527 and the other SNPs. Because epistatic interactions were only tested for SNPs with significant dominance association, other important epistatic interactions between pairs of SNPs across the studied traits would not have been found. Therefore, interpretation of the extent of epistatic effects should be done with caution. However, the epistatic interaction between SNP rs110361335 and the other SNPs may be biologically plausible given that this SNP is located within the *ICA1* gene, which has a role in the regulation of glucose metabolism [54]. Thus, it is necessary to investigate these SNPs in other populations to determine if their use in genomic selection would be beneficial in beef cattle crossbreeding program.

## Conclusions

In this work, we detected several SNPs with significant dominance (over or under) associations with growth and carcass marbling in multi-breed and crossbred beef cattle. The identified potential candidate genes within the vicinity of these SNPs (e.g. *U6atac, AGBL4, bta*-*mir*-*2888*-*1, REPIN1, ICA1 and NXPH1*) need further investigation to determine their relevance for crossbreeding programs and their ability to predict heterosis.

## Additional files

**Additional file 1: Table S1.** Identity, position, and effects of significantly associated additive SNPs obtained by single-SNP regression mixed model for growth and carcass traits in the purebred group, which included individuals with more than 80% of Angus, Hereford and Charolais breed proportions, respectively. **Table S2.** Identity, position, and effects of significantly associated additive SNPs obtained by single-SNP regression mixed model for growth and carcass traits in the crossbred group, which included Kinsella composite, Beefbooster TX composite (www.beefbooster.com) and two and more way crosses involving Angus, Hereford, Charolais, Gelbvieh, Simmental, Limousin, and Piedmontese breeds. **Table S3.** Identity, position, and effects of significantly associated additive SNPs obtained by single-SNP regression mixed model for growth and carcass traits in the combined population of beef cattle.

**Additional file 2: Figure S1.** Quantile–quantile (Q–Q) plot of *p* values for additive SNP association with growth traits in purebreds. **Figure S2.** Quantile–quantile (Q–Q) plot of *p* values for additive SNP association with carcass traits in purebreds. **Figure S3.** Quantile–quantile (Q–Q) plot of *p* values for additive SNP association with growth traits in crossbreds. **Figure S4.** Quantile–quantile (Q–Q) plot of *p* values for additive SNP association with carcass traits in crossbreds. **Figure S5.** Quantile–quantile (Q–Q) plot of *p* values for additive SNP association with growth traits in combined data. **Figure S6.** Quantile–quantile (Q–Q) plot of *p* values for additive SNP association with carcass traits in combined data. **Figure S7.** Quantile–quantile (Q–Q) plot of *p* values for dominance SNP association with growth traits in purebreds. **Figure S8.** Quantile–quantile (Q–Q) plot of *p* values for dominance SNP association with carcass traits in purebreds. **Figure S9.** Quantile–quantile (Q–Q) plot of *p* values for dominance SNP association with growth traits in crossbreds. **Figure S10.** Quantile–quantile (Q–Q) plot of *p* values for dominance SNP association with carcass traits in crossbreds. **Figure S11.** Quantile–quantile (Q–Q) plot of *p* values for dominance SNP association with growth traits in the combined data. **Figure S12.** Quantile–quantile (Q–Q) plot of *p* values for dominance SNP association with carcass traits in the combined data. **Figure S13.** Joint genome-wide association analysis for additive SNP effects on growth traits in purebreds. **Figure S14.** Joint genome-wide association analysis for additive SNP effects on carcass traits in purebreds. **Figure S15.** Joint genome-wide association analysis for additive SNP effects on growth traits in crossbreds. **Figure S16.** Joint genome-wide association analysis for additive SNP effects on carcass traits in crossbreds. **Figure S17.** Joint genome-wide association analysis for additive SNP effects on growth traits in the combined data. **Figure S18.** Joint genome-wide association analysis for additive SNP effects on carcass traits in the combined data. **Figure S19.** Joint genome-wide association analysis for dominance SNP effects on growth traits in purebreds. **Figure S20.** Joint genome-wide association analysis for dominance SNP effects on carcass traits in purebreds. **Figure S21.** Joint genome-wide association analysis for dominance SNP effects on growth traits in crossbreds. **Figure S22.** Joint genome-wide association analysis for dominance SNP effects on carcass traits in crossbreds. **Figure S23.** Joint genome-wide association analysis for dominance SNP effects on growth traits in the combined data. **Figure S24.** Joint genome-wide association analysis for dominance SNP effects on carcass traits in the combined data.
