# Supplementary material for: Genome-wide association scan for heterotic quantitative trait loci in multi-breed and crossbred beef cattle
Source: Genet Sel Evol. 2018 Oct 5;50:48. doi: 10.1186/s12711-018-0405-y (PMC6173862; doi:10.1186/s12711-018-0405-y)
Supplement: Supplementary file 1 — Additional file 1: Table S1. Identity, position, and effects of significantly associated additive SNPs obtained by single-SNP regression mixed model for growth and carcass traits in the purebred group, which included individuals with more than 80% of Angus, Hereford and Charolais breed proportions, respectively. Table S2. Identity, position, and effects of significantly associated additive SNPs obtained by single-SNP regression mixed model for growth and carcass traits in the crossbred group, which included Kinsella composite, Beefbooster TX composite (www.beefbooster.com) and two and more way crosses involving Angus, Hereford, Charolais, Gelbvieh, Simmental, Limousin, and Piedmontese breeds. Table S3. Identity, position, and effects of significantly associated additive SNPs obtained by single-SNP regression mixed model for growth and carcass traits in the combined population of beef cattle. [file 12711_2018_405_MOESM1_ESM.docx]

| **Table S1 Identities, positions, and effects of significantly associated additive SNPs obtained by single SNP regression mixed model for growth and carcass traits in purebred group^1^** | | | | | | | |
| --- | --- | --- | --- | --- | --- | --- | --- |
| **Trait^2^** | **SNP reference** | **BTA** | **Position (bp)** | **MAF** | ***P*-value** | **FDR (%)** | **Allele substitution effects** |
| BWT, Kg | rs29010454 | 4 | 43,175,304 | 0.074 | 7.90E-06 | 10 | -2.975±0.664 |
|  | rs110834363 | 6 | 38,939,012 | 0.395 | 4.20E-07 | 5 | 1.141±0.225 |
|  | rs109258862 | 6 | 40,629,318 | 0.408 | 1.37E-06 | 5 | 0.928±0.192 |
|  | rs43459808 | 6 | 42,057,261 | 0.393 | 8.20E-07 | 5 | 0.942±0.190 |
|  | rs41625576 | 7 | 93,289,032 | 0.190 | 7.70E-07 | 5 | -1.519±0.306 |
|  | rs109075576 | 13 | 2,011,011 | 0.228 | 2.52E-06 | 5 | -1.251±0.265 |
|  | rs108951661 | 13 | 76,705,709 | 0.163 | 1.26E-05 | 10 | -1.514±0.346 |
| ADG, Kg/d | rs41569493 | 9 | 45,767,377 | 0.034 | 7.70E-07 | 5 | -0.285±0.058 |
| REA, cm^2^ | rs43131664 | 1 | 22,630,942 | 0.222 | 3.49E-06 | 10 | 2.223±0.477 |
|  | rs42202519 | 21 | 54,247,557 | 0.303 | 6.86E-06 | 10 | -2.131±0.472 |
|  | rs110335461 | 24 | 41,289,711 | 0.132 | 4.29E-06 | 10 | -6.561±1.421 |
| LMY, % | rs41582924 | 21 | 21,017,358 | 0.466 | 2.10e-06 | 10 | -0.674±0.141 |
| YG | rs41582924 | 21 | 21,017,358 | 0.466 | 6.90e-07 | 5 | 0.124±0.025 |
| ^1^Purebred group included individuals with > 80% of Angus, Hereford and Charolais breed proportions, respectively.  ^2^Traits includes birth weight (BWT); average daily gain (ADG); rib eye area (REA); lean meat yield (LMY) and yield grade (YG)  BTA = *Bos taurus autosome*; bp = Base pairs; MAF = Minor allele frequency; FDR = False discovery rate | | | | | | | |

| **Table S2 Identities, positions, and effects of significantly associated additive SNPs obtained by single SNP regression mixed model for growth and carcass traits in crossbred group^1^** | | | | | | | |
| --- | --- | --- | --- | --- | --- | --- | --- |
| **Trait^2^** | **SNP reference** | **BTA** | **Position (bp)** | **MAF** | ***P*-value** | **FDR (%)** | **Allele substitution effects** |
| BWT, Kg | rs43230873 | 1 | 52,696,732 | 0.255 | 0.000245 | 10 | 0.630±0.172 |
|  | rs43111100 | 1 | 120,326,805 | 0.161 | 0.000187 | 10 | -1.068±0.286 |
|  | rs41633180 | 2 | 8,430,514 | 0.433 | 0.000200 | 10 | 0.502±0.135 |
|  | rs41598010 | 2 | 109,740,844 | 0.143 | 0.000101 | 10 | 1.265±0.325 |
|  | rs109084099 | 3 | 33,367,402 | 0.478 | 9.97E-05 | 10 | 0.511±0.131 |
|  | rs42659843 | 4 | 117,086,858 | 0.133 | 0.000241 | 10 | 1.113±0.303 |
|  | rs41622168 | 5 | 102,084,969 | 0.390 | 0.000173 | 10 | 0.540±0.144 |
|  | rs41649876 | 6 | 27,433,375 | 0.471 | 2.00E-08 | 5 | 0.747±0.132 |
|  | rs41613697 | 6 | 30,403,794 | 0.394 | 0.000146 | 10 | 0.540±0.142 |
|  | rs29012331 | 6 | 31,067,604 | 0.387 | 0.000171 | 10 | -0.542±0.144 |
|  | rs41622316 | 6 | 31,275,687 | 0.193 | 1.74E-05 | 5 | -1.116±0.259 |
|  | rs109717572 | 6 | 32,241,952 | 0.119 | 8.29E-05 | 10 | -1.259±0.319 |
|  | rs41665871 | 6 | 35,611,267 | 0.493 | 0.000252 | 10 | 0.494±0.135 |
|  | rs110754910 | 6 | 35,728,464 | 0.378 | 0.000140 | 10 | 0.550±0.144 |
|  | rs108983635 | 6 | 35,922,378 | 0.230 | 0.000148 | 10 | 0.748±0.197 |
|  | rs109704656 | 6 | 36,062,803 | 0.315 | 4.00E-08 | 5 | 0.847±0.154 |
|  | rs110332219 | 6 | 36,986,502 | 0.425 | 6.90E-07 | 5 | 0.667±0.134 |
|  | rs110587419 | 6 | 37,019,972 | 0.320 | 1.00E-08 | 5 | 0.933±0.160 |
|  | rs110767541 | 6 | 37,104,193 | 0.467 | 6.02E-05 | 5 | 0.534±0.133 |
|  | rs41627896 | 6 | 37,218,883 | 0.385 | 3.30E-07 | 5 | 0.742±0.145 |
|  | rs109998457 | 6 | 37,252,345 | 0.384 | 3.90E-07 | 5 | 0.739±0.145 |
|  | rs109514983 | 6 | 37,584,088 | 0.224 | 1.00E-08 | 5 | -1.061±0.185 |
|  | rs109641632 | 6 | 37,653,391 | 0.156 | 3.79E-05 | 5 | -1.010±0.245 |
|  | rs41650794 | 6 | 37,839,427 | 0.482 | 3.44E-06 | 5 | -0.616±0.132 |
|  | rs110909199 | 6 | 37,868,743 | 0.491 | 1.00E-08 | 5 | 0.77±0.135 |
|  | rs29010895 | 6 | 38,042,011 | 0.483 | 1.00E-08 | 5 | -0.875±0.136 |
|  | rs111018320 | 6 | 38,133,743 | 0.427 | 4.00E-08 | 5 | 0.758±0.138 |
|  | rs110668054 | 6 | 38,464,203 | 0.352 | 1.00E-08 | 5 | 0.895±0.148 |
|  | rs109315366 | 6 | 38,576,012 | 0.435 | 1.00E-08 | 5 | -1.492±0.136 |
|  | rs41569339 | 6 | 38,689,886 | 0.249 | 0.000205 | 10 | 0.613±0.165 |
|  | rs109795992 | 6 | 38,825,835 | 0.461 | 1.00E-08 | 5 | -0.895±0.134 |
|  | rs110961068 | 6 | 38,845,992 | 0.453 | 1.00E-08 | 5 | 0.869±0.135 |
|  | rs109294917 | 6 | 38,869,785 | 0.488 | 1.00E-08 | 5 | 1.091±0.133 |
|  | rs110834363 | 6 | 38,939,012 | 0.453 | 1.00E-08 | 5 | 1.247±0.134 |
|  | rs110335851 | 6 | 39,034,201 | 0.256 | 0.000229 | 10 | -0.683±0.185 |
|  | rs43027303 | 6 | 39,172,862 | 0.243 | 4.00E-08 | 5 | 0.996±0.180 |
|  | rs110240246 | 6 | 39,257,620 | 0.444 | 1.00E-09 | 5 | 1.252±0.137 |
|  | rs110012183 | 6 | 39,346,170 | 0.438 | 1.00E-08 | 5 | 1.215±0.134 |
|  | rs110517212 | 6 | 39,371,150 | 0.319 | 5.50E-07 | 5 | 0.774±0.154 |
|  | rs110392766 | 6 | 395,299,73 | 0.484 | 1.00E-08 | 5 | 0.529±0.132 |
|  | rs109692323 | 6 | 39,597,740 | 0.324 | 1.12E-05 | 5 | 0.689±0.157 |
|  | rs109090869 | 6 | 39,752,891 | 0.455 | 1.00E-08 | 5 | -0.860±0.131 |
|  | rs43458270 | 6 | 39,837,065 | 0.277 | 3.72E-06 | 5 | 0.814±0.176 |
|  | rs43467099 | 6 | 40,063,618 | 0.311 | 8.00E-08 | 5 | 0.883±0.165 |
|  | rs41650820 | 6 | 40,086,624 | 0.372 | 5.60E-07 | 5 | -0.699±0.139 |
|  | rs109258862 | 6 | 40,629,318 | 0.359 | 1.00E-08 | 5 | 0.859±0.149 |
|  | rs42521925 | 6 | 40,893,067 | 0.467 | 3.27E-06 | 5 | -0.632±0.136 |
|  | rs43461214 | 6 | 41,178,449 | 0.406 | 7.91E-05 | 10 | 0.543±0.137 |
|  | rs43463290 | 6 | 41,530,548 | 0.245 | 9.16E-05 | 10 | -0.783±0.200 |
|  | rs109862934 | 6 | 41,588,847 | 0.250 | 7.74E-05 | 10 | 0.738±0.186 |
|  | rs43462203 | 6 | 42,567,228 | 0.251 | 4.37E-05 | 5 | -0.817±0.200 |
|  | rs109945067 | 6 | 44,441,744 | 0.215 | 0.000199 | 10 | -0.784±0.211 |
|  | rs29025709 | 6 | 46,260,375 | 0.379 | 4.85E-05 | 5 | 0.576±0.142 |
|  | rs109055778 | 6 | 46,599,570 | 0.492 | 0.000129 | 10 | -0.522±0.136 |
|  | rs109550729 | 6 | 46,820,055 | 0.278 | 0.000134 | 10 | -0.667±0.174 |
|  | rs29017713 | 6 | 46,936,182 | 0.477 | 0.000148 | 10 | -0.490±0.129 |
|  | rs42577887 | 6 | 51,669,513 | 0.443 | 0.000141 | 10 | -0.514±0.135 |
|  | rs41658480 | 6 | 54,237,782 | 0.490 | 1.15E-05 | 5 | -0.579±0.132 |
|  | rs109823008 | 6 | 86,354,888 | 0.341 | 0.000127 | 10 | -0.566±0.148 |
|  | rs110810914 | 6 | 87,281,196 | 0.280 | 4.17E-06 | 5 | 0.775±0.168 |
|  | rs43490545 | 6 | 109,345,352 | 0.241 | 0.000199 | 10 | -0.664±0.178 |
|  | rs43488867 | 6 | 112,204,503 | 0.164 | 1.35E-06 | 5 | 1.316±0.272 |
|  | rs41628990 | 7 | 31,136,178 | 0.271 | 8.34E-05 | 10 | -0.713±0.181 |
|  | rs42546442 | 7 | 34,861,506 | 0.409 | 0.000222 | 10 | -0.517±0.140 |
|  | rs41615523 | 7 | 35,099,734 | 0.439 | 0.000182 | 10 | 0.505±0.135 |
|  | rs43571536 | 7 | 42,052,923 | 0.270 | 0.000188 | 10 | -0.647±0.173 |
|  | rs41763830 | 7 | 87,359,924 | 0.134 | 4.04E-05 | 5 | -1.308±0.318 |
|  | rs110652403 | 7 | 90,460,692 | 0.271 | 0.000145 | 10 | -0.593±0.156 |
|  | rs109819349 | 7 | 93,007,435 | 0.295 | 5.71E-06 | 5 | -0.754±0.166 |
|  | rs41625563 | 7 | 93,073,890 | 0.490 | 8.26E-06 | 5 | -0.595±0.133 |
|  | rs110059753 | 7 | 93,218,452 | 0.398 | 2.00E-08 | 5 | -0.789±0.140 |
|  | rs41656461 | 8 | 61,838,644 | 0.324 | 8.29E-05 | 10 | -0.646±0.164 |
|  | rs110291155 | 9 | 57,658,825 | 0.157 | 4.93E-05 | 5 | -1.330±0.327 |
|  | rs43606817 | 9 | 82,176,129 | 0.346 | 0.000208 | 10 | -0.549±0.148 |
|  | rs43003144 | 9 | 91,580,703 | 0.367 | 6.41E-05 | 5 | -0.571±0.143 |
|  | rs108945111 | 10 | 101,168,543 | 0.405 | 0.000106 | 10 | 0.545±0.141 |
|  | rs110038905 | 11 | 91,995,272 | 0.430 | 1.50E-05 | 5 | 0.596±0.138 |
|  | rs41257042 | 12 | 90,621,015 | 0.287 | 0.000107 | 10 | 0.625±0.161 |
|  | rs110765746 | 13 | 57,524,735 | 0.130 | 0.000151 | 10 | 1.244±0.328 |
|  | rs41628383 | 14 | 23,853,811 | 0.462 | 0.000121 | 10 | -0.525±0.137 |
|  | rs42649775 | 14 | 24,437,778 | 0.232 | 4.32E-06 | 5 | -0.940±0.204 |
|  | rs42646660 | 14 | 24,524,205 | 0.180 | 2.00E-08 | 5 | -1.322±0.235 |
|  | rs42646708 | 14 | 24,573,257 | 0.169 | 3.04E-06 | 5 | 1.170±0.250 |
|  | rs41724332 | 14 | 24,643,266 | 0.152 | 6.11E-05 | 5 | -1.172±0.292 |
|  | rs29021334 | 14 | 25,612,510 | 0.238 | 4.14E-06 | 5 | -0.868±0.188 |
|  | rs42304759 | 14 | 26,196,375 | 0.424 | 9.07E-05 | 10 | 0.550±0.140 |
|  | rs42303720 | 14 | 26,264,142 | 0.422 | 2.38E-05 | 5 | -0.594±0.140 |
|  | rs41726059 | 14 | 26,473,490 | 0.412 | 0.000179 | 10 | -0.539±0.144 |
|  | rs109375770 | 14 | 26,685,204 | 0.460 | 0.000224 | 10 | -0.505±0.137 |
|  | rs41724601 | 14 | 26,713,734 | 0.414 | 4.30E-05 | 5 | -0.580±0.142 |
|  | rs41724536 | 14 | 26,766,010 | 0.382 | 9.92E-05 | 10 | -0.578±0.148 |
|  | rs42404949 | 14 | 26,949,215 | 0.490 | 1.33E-05 | 5 | 0.602±0.138 |
|  | rs109198793 | 14 | 76,524,093 | 0.334 | 0.000167 | 10 | -0.567±0.151 |
|  | rs41633638 | 14 | 76,585,288 | 0.315 | 0.000189 | 10 | -0.574±0.154 |
|  | rs41573465 | 15 | 55,930,081 | 0.400 | 0.000239 | 10 | 0.536±0.146 |
|  | rs42402217 | 15 | 63,281,076 | 0.207 | 0.000155 | 10 | -0.843±0.223 |
|  | rs110173350 | 16 | 24,721,294 | 0.073 | 8.76E-05 | 10 | -1.878±0.478 |
|  | rs41585732 | 19 | 25,458,074 | 0.498 | 0.000206 | 10 | -0.513±0.138 |
|  | rs110708961 | 20 | 4,845,639 | 0.397 | 6.21E-05 | 5 | 0.561±0.140 |
|  | rs110264113 | 22 | 55,511,639 | 0.393 | 1.87E-05 | 5 | -0.586±0.137 |
|  | rs41572498 | 22 | 55,586,234 | 0.385 | 5.01E-05 | 5 | 0.568±0.140 |
|  | rs109406560 | 23 | 20,193,086 | 0.146 | 8.88E-05 | 10 | 1.214±0.309 |
|  | rs41587216 | 23 | 22,300,959 | 0.453 | 0.000175 | 10 | -0.503±0.134 |
|  | rs110996629 | 23 | 34,575,024 | 0.219 | 9.97E-06 | 5 | 1.007±0.228 |
|  | rs110561429 | 25 | 10,896,067 | 0.474 | 0.000190 | 10 | -0.484±0.130 |
|  | rs41605882 | 25 | 35,587,219 | 0.236 | 5.19E-05 | 5 | -0.847±0.209 |
|  | rs110130856 | 25 | 38,330,924 | 0.354 | 4.19E-05 | 5 | -0.603±0.147 |
|  | rs42138871 | 28 | 24,719,677 | 0.341 | 0.000166 | 10 | -0.557±0.148 |
|  | rs41257175 | 29 | 26,009,040 | 0.339 | 8.47E-05 | 10 | 0.582±0.148 |
| WWT, Kg | rs29013268 | 6 | 35,788,711 | 0.231 | 2.42E-06 | 5 | 4.349±0.921 |
|  | rs111018320 | 6 | 38,133,743 | 0.427 | 1.00E-08 | 5 | 4.051±0.616 |
|  | rs110668054 | 6 | 38,464,203 | 0.352 | 1.00E-08 | 5 | 4.102±0.669 |
|  | rs109315366 | 6 | 38,576,012 | 0.435 | 1.00E-08 | 5 | -5.459±0.620 |
|  | rs109795992 | 6 | 38,825,835 | 0.461 | 4.81E-05 | 10 | -2.474±0.608 |
|  | rs109294917 | 6 | 38,869,785 | 0.488 | 2.00E-08 | 5 | 3.415±0.606 |
|  | rs110834363 | 6 | 38,939,012 | 0.453 | 1.00E-09 | 5 | 4.159±0.614 |
|  | rs43027303 | 6 | 39,172,862 | 0.243 | 2.11E-05 | 10 | 3.538±0.831 |
|  | rs110240246 | 6 | 39,257,620 | 0.444 | 1.00E-08 | 5 | 3.637±0.616 |
|  | rs110012183 | 6 | 39,346,170 | 0.438 | 1.00E-08 | 5 | 3.531±0.605 |
|  | rs109090869 | 6 | 39,752,891 | 0.455 | 2.17E-05 | 10 | -2.541±0.598 |
|  | rs41650820 | 6 | 40,086,624 | 0.372 | 2.62E-05 | 10 | -2.666±0.633 |
|  | rs109945067 | 6 | 44,441,744 | 0.215 | 2.02E-05 | 10 | -4.051±0.949 |
|  | rs109055778 | 6 | 46,599,570 | 0.492 | 2.95E-05 | 10 | -2.525±0.604 |
|  | rs109819349 | 7 | 93,007,435 | 0.295 | 1.67E-05 | 10 | -3.350±0.777 |
|  | rs110059753 | 7 | 93,218,452 | 0.398 | 7.20E-07 | 5 | -3.195±0.644 |
|  | rs42431785 | 10 | 95,274,618 | 0.415 | 1.88E-05 | 10 | -2.628±0.614 |
|  | rs41631541 | 13 | 63,257,337 | 0.404 | 3.44E-05 | 10 | -2.582±0.623 |
|  | rs42646660 | 14 | 24,524,205 | 0.180 | 2.53E-05 | 10 | -4.531±1.074 |
|  | rs43350564 | 20 | 4,618,689 | 0.339 | 5.21E-06 | 5 | 3.119±0.684 |
|  | rs42016981 | 22 | 57,486,105 | 0.242 | 2.88E-05 | 10 | 3.537±0.844 |
| PDG, Kg/d | rs110450381 | 2 | 104,297,699 | 0.281 | 8.55E-06 | 5 | 0.021±0.005 |
|  | rs43097370 | 6 | 23,183,379 | 0.335 | 2.66E-06 | 5 | 0.018±0.004 |
|  | rs111018320 | 6 | 38,133,743 | 0.427 | 1.00E-08 | 5 | 0.021±0.004 |
|  | rs110668054 | 6 | 38,464,203 | 0.352 | 1.92E-05 | 10 | 0.016±0.004 |
|  | rs109315366 | 6 | 38,576,012 | 0.435 | 1.00E-08 | 5 | -0.023±0.004 |
|  | rs109294917 | 6 | 38,869,785 | 0.488 | 5.04E-06 | 5 | 0.016±0.004 |
|  | rs110834363 | 6 | 38,939,012 | 0.453 | 8.00E-08 | 5 | 0.019±0.004 |
|  | rs110240246 | 6 | 39,257,620 | 0.444 | 1.24E-05 | 10 | 0.016±0.004 |
|  | rs110012183 | 6 | 39,346,170 | 0.438 | 1.78E-06 | 5 | 0.017±0.004 |
|  | rs29010203 | 13 | 45,925,987 | 0.441 | 4.61E-06 | 5 | 0.016±0.003 |
| ADG, Kg | rs110606974 | 3 | 29,358,344 | 0.497 | 9.18E-05 | 10 | 0.023±0.006 |
|  | rs41655687 | 4 | 63,906,905 | 0.280 | 8.74E-05 | 10 | 0.029±0.007 |
|  | rs43415920 | 4 | 113,506,092 | 0.252 | 3.50E-05 | 10 | 0.033±0.008 |
|  | rs43416912 | 4 | 113,532,717 | 0.253 | 2.48E-05 | 5 | 0.034±0.008 |
|  | rs43705625 | 6 | 28,576,090 | 0.336 | 6.14E-05 | 10 | 0.027±0.007 |
|  | rs109704656 | 6 | 36,062,803 | 0.315 | 4.41E-05 | 10 | 0.028±0.007 |
|  | rs110587419 | 6 | 37,019,972 | 0.320 | 1.21E-05 | 5 | 0.030±0.007 |
|  | rs29010895 | 6 | 38,042,011 | 0.483 | 1.88E-06 | 5 | -0.027±0.006 |
|  | rs111018320 | 6 | 38,133,743 | 0.427 | 1.26E-05 | 5 | 0.025±0.006 |
|  | rs110668054 | 6 | 38,464,203 | 0.352 | 1.00E-07 | 5 | 0.034±0.006 |
|  | rs109315366 | 6 | 38,576,012 | 0.435 | 1.00E-08 | 5 | -0.053±0.006 |
|  | rs109795992 | 6 | 38,825,835 | 0.461 | 1.00E-08 | 5 | -0.037±0.006 |
|  | rs110961068 | 6 | 38,845,992 | 0.453 | 1.00E-08 | 5 | 0.036±0.006 |
|  | rs109294917 | 6 | 38,869,785 | 0.488 | 1.00E-08 | 5 | 0.042±0.006 |
|  | rs110834363 | 6 | 38,939,012 | 0.453 | 1.00E-08 | 5 | 0.051±0.006 |
|  | rs110335851 | 6 | 39,034,201 | 0.256 | 1.10E-07 | 5 | -0.042±0.008 |
|  | rs110240246 | 6 | 39,257,620 | 0.444 | 1.00E-08 | 5 | 0.052±0.006 |
|  | rs110012183 | 6 | 39,346,170 | 0.438 | 1.00E-08 | 5 | 0.048±0.006 |
|  | rs109090869 | 6 | 39,752,891 | 0.455 | 8.70E-07 | 5 | -0.028±0.006 |
|  | rs43467099 | 6 | 40,063,618 | 0.311 | 4.85E-05 | 10 | 0.028±0.007 |
|  | rs43461214 | 6 | 41,178,449 | 0.406 | 5.51E-05 | 10 | 0.024±0.006 |
|  | rs43457653 | 6 | 41,914,851 | 0.496 | 2.61E-05 | 5 | -0.024±0.006 |
|  | rs43459808 | 6 | 42,057,261 | 0.494 | 1.21E-05 | 5 | 0.025±0.006 |
|  | rs41651273 | 6 | 42,655,795 | 0.320 | 6.95E-06 | 5 | -0.031±0.007 |
|  | rs29025709 | 6 | 46,260,375 | 0.379 | 5.10E-07 | 5 | 0.031±0.006 |
|  | rs110207017 | 6 | 46,533,015 | 0.456 | 5.92E-05 | 10 | -0.023±0.006 |
|  | rs109055778 | 6 | 46,599,570 | 0.492 | 6.72E-05 | 10 | -0.023±0.006 |
|  | rs41658480 | 6 | 54,237,782 | 0.490 | 2.04E-05 | 5 | -0.024±0.006 |
|  | rs109819349 | 7 | 93,007,435 | 0.295 | 7.12E-05 | 10 | -0.029±0.007 |
|  | rs41625563 | 7 | 93,073,890 | 0.490 | 5.30E-05 | 10 | -0.023±0.006 |
|  | rs110059753 | 7 | 93,218,452 | 0.398 | 3.00E-08 | 5 | -0.034±0.006 |
|  | rs43650985 | 10 | 91,846,997 | 0.362 | 1.70E-05 | 5 | -0.027±0.006 |
|  | rs41718711 | 12 | 65,662,261 | 0.248 | 7.54E-05 | 10 | 0.033±0.008 |
|  | rs42646660 | 14 | 24,524,205 | 0.180 | 6.50E-07 | 5 | -0.053±0.011 |
|  | rs42646708 | 14 | 24,573,257 | 0.169 | 5.92E-05 | 10 | 0.046±0.011 |
|  | rs42306877 | 14 | 26,035,082 | 0.463 | 8.70E-05 | 10 | 0.023±0.006 |
|  | rs29021060 | 16 | 35,835,922 | 0.445 | 4.10E-05 | 10 | -0.023±0.006 |
|  | rs29020544 | 18 | 43,327,273 | 0.404 | 2.50E-06 | 5 | 0.027±0.006 |
|  | rs110474207 | 21 | 26,405,185 | 0.417 | 6.09E-05 | 10 | -0.024±0.006 |
|  | rs42115397 | 27 | 10,349,453 | 0.289 | 4.93E-05 | 10 | -0.029±0.007 |
| YWT, Kg | rs110633489 | 2 | 14,717,851 | 0.428 | 7.64E-05 | 10 | -3.510±0.886 |
|  | rs111028820 | 2 | 24,773,731 | 0.356 | 8.58E-05 | 10 | 3.867±0.984 |
|  | rs43050734 | 5 | 864,491 | 0.426 | 7.34E-06 | 5 | 3.907±0.870 |
|  | rs41660811 | 6 | 28,181,355 | 0.212 | 0.000117 | 10 | 5.009±1.299 |
|  | rs41665871 | 6 | 35,611,267 | 0.493 | 3.87E-06 | 5 | 4.026±0.871 |
|  | rs29012836 | 6 | 35,648,890 | 0.205 | 5.00E-08 | 5 | -8.619±1.576 |
|  | rs29013268 | 6 | 35,788,711 | 0.231 | 1.00E-08 | 5 | 9.057±1.390 |
|  | rs109704656 | 6 | 36,062,803 | 0.315 | 0.000113 | 10 | 3.86±0.999 |
|  | rs41592814 | 6 | 36,277,967 | 0.317 | 0.000108 | 10 | -4.089±1.055 |
|  | rs110332219 | 6 | 36,986,502 | 0.425 | 1.59E-05 | 5 | 3.778±0.874 |
|  | rs110587419 | 6 | 37,019,972 | 0.320 | 4.24E-05 | 5 | 4.198±1.024 |
|  | rs110483628 | 6 | 37,323,779 | 0.309 | 3.55E-05 | 5 | 4.305±1.040 |
|  | rs41605197 | 6 | 37,399,296 | 0.240 | 6.58E-06 | 5 | -5.423±1.202 |
|  | rs109514983 | 6 | 37,584,088 | 0.224 | 5.46E-06 | 5 | -5.576±1.224 |
|  | rs110909199 | 6 | 37,868,743 | 0.491 | 5.40E-07 | 5 | 4.387±0.874 |
|  | rs29010895 | 6 | 38,042,011 | 0.483 | 1.00E-08 | 5 | -5.175±0.879 |
|  | rs111018320 | 6 | 38,133,743 | 0.427 | 1.00E-08 | 5 | 6.573±0.889 |
|  | rs109454650 | 6 | 38,173,166 | 0.421 | 2.72E-05 | 5 | -3.853±0.917 |
|  | rs109248021 | 6 | 38,203,273 | 0.486 | 3.50E-07 | 5 | -4.447±0.871 |
|  | rs110668054 | 6 | 38,464,203 | 0.352 | 1.00E-08 | 5 | 8.233±0.970 |
|  | rs109315366 | 6 | 38,576,012 | 0.435 | 1.00E-08 | 5 | -10.09±0.892 |
|  | rs109331996 | 6 | 38,627,070 | 0.183 | 8.95E-05 | 10 | 6.193±1.579 |
|  | rs41569339 | 6 | 38,689,886 | 0.249 | 0.000125 | 10 | 4.260±1.110 |
|  | rs109795992 | 6 | 38,825,835 | 0.461 | 8.00E-08 | 5 | -4.658±0.867 |
|  | rs110961068 | 6 | 38,845,992 | 0.453 | 9.90E-07 | 5 | 4.270±0.871 |
|  | rs109294917 | 6 | 38,869,785 | 0.488 | 1.00E-08 | 5 | 6.370±0.867 |
|  | rs110834363 | 6 | 38,939,012 | 0.453 | 1.00E-08 | 5 | 7.511±0.879 |
|  | rs43027303 | 6 | 39,172,862 | 0.243 | 4.00E-08 | 5 | 6.483±1.179 |
|  | rs110240246 | 6 | 39,257,620 | 0.444 | 1.00E-08 | 5 | 6.943±0.884 |
|  | rs110012183 | 6 | 39,346,170 | 0.438 | 1.00E-08 | 5 | 7.321±0.868 |
|  | rs109692323 | 6 | 39,597,740 | 0.324 | 2.24E-06 | 5 | 4.894±1.033 |
|  | rs110266353 | 6 | 39,721,727 | 0.171 | 4.31E-05 | 5 | 7.066±1.725 |
|  | rs109090869 | 6 | 39,752,891 | 0.455 | 2.30E-07 | 5 | -4.481±0.865 |
|  | rs43458270 | 6 | 39,837,065 | 0.277 | 0.000122 | 10 | 4.478±1.165 |
|  | rs43467099 | 6 | 40,063,618 | 0.311 | 5.04E-05 | 5 | 4.191±1.033 |
|  | rs41650820 | 6 | 40,086,624 | 0.372 | 1.00E-08 | 5 | -5.454±0.909 |
|  | rs42989288 | 6 | 40,478,325 | 0.326 | 0.000142 | 10 | 3.864±1.015 |
|  | rs109258862 | 6 | 40,629,318 | 0.359 | 1.30E-07 | 5 | 5.085±0.961 |
|  | rs42521925 | 6 | 40,893,067 | 0.467 | 9.82E-06 | 5 | -3.932±0.888 |
|  | rs43461214 | 6 | 41,178,449 | 0.406 | 1.80E-07 | 5 | 4.712±0.902 |
|  | rs43457653 | 6 | 41,914,851 | 0.496 | 4.22E-05 | 5 | -3.502±0.854 |
|  | rs41595967 | 6 | 42,294,971 | 0.409 | 7.85E-05 | 10 | 3.618±0.915 |
|  | rs109945067 | 6 | 44,441,744 | 0.215 | 5.46E-05 | 5 | -5.596±1.385 |
|  | rs29025709 | 6 | 46,260,375 | 0.379 | 9.04E-05 | 10 | 3.653±0.932 |
|  | rs109032578 | 6 | 53,871,559 | 0.374 | 4.94E-05 | 5 | -3.957±0.974 |
|  | rs109073443 | 6 | 117,384,629 | 0.300 | 5.84E-05 | 5 | 4.389±1.091 |
|  | rs42179919 | 7 | 89,734,142 | 0.183 | 0.000134 | 10 | 6.002±1.570 |
|  | rs109769526 | 7 | 91,693,979 | 0.427 | 5.02E-05 | 5 | -3.502±0.863 |
|  | rs109819349 | 7 | 93,007,435 | 0.295 | 4.96E-05 | 5 | -4.534±1.116 |
|  | rs110059753 | 7 | 93,218,452 | 0.398 | 3.50E-07 | 5 | -4.701±0.922 |
|  | rs42403117 | 8 | 4,098,134 | 0.123 | 0.000102 | 10 | -9.571±2.460 |
|  | rs109976835 | 8 | 34,536,574 | 0.201 | 6.29E-05 | 10 | 6.135±1.531 |
|  | rs109493204 | 8 | 57,714,648 | 0.242 | 1.96E-05 | 5 | -5.403±1.264 |
|  | rs109832919 | 8 | 61,536,940 | 0.264 | 1.12E-05 | 5 | -4.864±1.106 |
|  | rs109733774 | 11 | 25,418,833 | 0.392 | 0.000127 | 10 | 3.537±0.922 |
|  | rs109736037 | 11 | 48,545,880 | 0.439 | 7.33E-05 | 10 | -3.743±0.943 |
|  | rs41612860 | 11 | 77,076,811 | 0.382 | 5.21E-05 | 5 | -3.677±0.908 |
|  | rs41612874 | 11 | 77,330,791 | 0.404 | 0.000127 | 10 | -3.515±0.917 |
|  | rs29021098 | 12 | 28,052,265 | 0.322 | 6.51E-06 | 5 | 4.740±1.050 |
|  | rs110508720 | 13 | 7,564,399 | 0.283 | 1.25E-05 | 5 | 4.956±1.133 |
|  | rs41631541 | 13 | 63,257,337 | 0.404 | 4.79E-06 | 5 | -4.066±0.888 |
|  | rs41576569 | 13 | 63,369,536 | 0.453 | 9.84E-05 | 10 | -3.462±0.888 |
|  | rs110030253 | 13 | 63,391,193 | 0.453 | 0.000116 | 10 | 3.429±0.889 |
|  | rs42649775 | 14 | 24,437,778 | 0.232 | 1.39E-06 | 5 | -6.562±1.357 |
|  | rs42646660 | 14 | 24,524,205 | 0.180 | 2.60E-07 | 5 | -8.105±1.57 |
|  | rs109394917 | 14 | 40,684,301 | 0.346 | 1.91E-06 | 5 | -4.667±0.978 |
|  | rs41632162 | 14 | 62,751,254 | 0.487 | 7.68E-05 | 10 | -3.405±0.86 |
|  | rs43350564 | 20 | 4,618,689 | 0.339 | 1.80E-07 | 5 | 5.122±0.979 |
|  | rs110708961 | 20 | 4,845,639 | 0.397 | 7.49E-06 | 5 | 4.057±0.905 |
|  | rs109291010 | 21 | 21,703,503 | 0.238 | 9.94E-05 | 10 | -5.069±1.301 |
|  | rs42847918 | 26 | 2,001,199 | 0.257 | 1.51E-05 | 5 | -5.046±1.165 |
| HCW, Kg | rs109514983 | 6 | 37,584,088 | 0.224 | 2.97E-05 | 10 | -5.520±1.319 |
|  | rs111018320 | 6 | 38,133,743 | 0.427 | 1.61E-06 | 5 | 4.072±0.847 |
|  | rs110668054 | 6 | 38,464,203 | 0.352 | 1.00E-08 | 5 | 6.336±0.927 |
|  | rs109315366 | 6 | 38,576,012 | 0.435 | 1.00E-08 | 5 | -6.015±0.838 |
|  | rs41569339 | 6 | 38,689,886 | 0.249 | 4.93E-05 | 10 | 4.330±1.065 |
|  | rs109795992 | 6 | 38,825,835 | 0.461 | 1.62E-05 | 5 | -3.645±0.844 |
|  | rs109294917 | 6 | 38,869,785 | 0.488 | 1.00E-08 | 5 | 4.841±0.831 |
|  | rs110834363 | 6 | 38,939,012 | 0.453 | 1.00E-08 | 5 | 6.248±0.843 |
|  | rs110240246 | 6 | 39,257,620 | 0.444 | 1.00E-08 | 5 | 5.829±0.856 |
|  | rs110012183 | 6 | 39,346,170 | 0.438 | 1.00E-08 | 5 | 5.796±0.829 |
|  | rs110266353 | 6 | 39,721,727 | 0.171 | 6.42E-06 | 5 | 7.838±1.734 |
|  | rs41650820 | 6 | 40,086,624 | 0.372 | 1.00E-08 | 5 | -5.046±0.873 |
|  | rs109258862 | 6 | 40,629,318 | 0.359 | 1.20E-07 | 5 | 4.876±0.919 |
|  | rs42521925 | 6 | 40,893,067 | 0.467 | 4.24E-05 | 10 | -3.430±0.836 |
|  | rs43461214 | 6 | 41,178,449 | 0.406 | 6.14E-06 | 5 | 3.850±0.850 |
|  | rs29025709 | 6 | 46,260,375 | 0.379 | 4.21E-05 | 10 | 3.568±0.870 |
|  | rs29016515 | 6 | 117,549,481 | 0.402 | 2.97E-05 | 10 | 3.683±0.880 |
|  | rs109819349 | 7 | 93,007,435 | 0.295 | 9.00E-06 | 5 | -4.942±1.111 |
|  | rs110059753 | 7 | 93,218,452 | 0.398 | 1.00E-08 | 5 | -5.613±0.886 |
|  | rs43707538 | 10 | 36,374,166 | 0.441 | 1.97E-05 | 5 | 3.634±0.850 |
|  | rs42646660 | 14 | 24,524,205 | 0.180 | 5.00E-08 | 5 | -8.024±1.465 |
|  | rs42646708 | 14 | 24,573,257 | 0.169 | 2.43E-05 | 10 | 6.819±1.612 |
|  | rs42717152 | 15 | 43,912,856 | 0.478 | 1.70E-05 | 5 | 3.56±0.826 |
| FAT, mm | rs109315366 | 6 | 38,576,012 | 0.435 | 1.11E-05 | 10 | 0.448±0.102 |
|  | rs109294917 | 6 | 38,869,785 | 0.488 | 8.10E-07 | 5 | -0.494±0.100 |
|  | rs110834363 | 6 | 38,939,012 | 0.453 | 1.60E-07 | 5 | -0.535±0.102 |
|  | rs110240246 | 6 | 39,257,620 | 0.444 | 3.33E-06 | 5 | -0.483±0.104 |
|  | rs110012183 | 6 | 39,346,170 | 0.438 | 6.31E-06 | 10 | -0.457±0.101 |
|  | rs109718273 | 8 | 36,293,755 | 0.382 | 4.76E-06 | 10 | -0.506±0.110 |
| REA, cm^2^ | rs110668054 | 6 | 38,464,203 | 0.352 | 1.00E-08 | 5 | 1.704±0.298 |
|  | rs109315366 | 6 | 38,576,012 | 0.435 | 1.00E-08 | 5 | -1.527±0.268 |
|  | rs110834363 | 6 | 38,939,012 | 0.453 | 1.88E-06 | 5 | 1.296±0.271 |
|  | rs110240246 | 6 | 39,257,620 | 0.444 | 8.87E-06 | 5 | 1.232±0.277 |
|  | rs110012183 | 6 | 39,346,170 | 0.438 | 5.00E-08 | 5 | 1.467±0.268 |
|  | rs42521925 | 6 | 40,893,067 | 0.467 | 1.36E-06 | 5 | -1.298±0.268 |
|  | rs109987408 | 6 | 68,546,212 | 0.468 | 3.81E-06 | 5 | -1.217±0.263 |
|  | rs41611571 | 6 | 107,720,898 | 0.103 | 9.78E-06 | 5 | -3.830±0.865 |
|  | rs42658517 | 6 | 107,742,938 | 0.161 | 7.16E-06 | 5 | -2.484±0.552 |
|  | rs109819349 | 7 | 93,007,435 | 0.295 | 1.00E-08 | 5 | -2.229±0.355 |
|  | rs41625563 | 7 | 93,073,890 | 0.490 | 1.17E-05 | 5 | -1.150±0.262 |
|  | rs110059753 | 7 | 93,218,452 | 0.398 | 1.00E-08 | 5 | -1.867±0.285 |
| MBS | rs29009626 | 7 | 93,886,136 | 0.491 | 2.75e-06 | 10 | -8.130±1.730 |
|  | rs110258661 | 25 | 42,726,313 | 0.317 | 2.86e-06 | 10 | -9.961±2.123 |
| ^1^Crossbred group included Kinsella composite, Beefbooster TX composite (www.beefbooster.com) and two and more way crosses involving Angus, Hereford, Charolais, Gelbvieh, Simmental, Limousin, and Piedmontese breeds.  ^2^Traits includes birth weight (BWT); weaning weight (WWT); pre-weaning daily gain (PDG); average daily gain (ADG); and yearling weight (YWT); hot carcass weight (HCW); back fat thickness (FAT); rib eye area (REA); marbling score (MBS)  BTA = *Bos taurus autosome*; bp = Base pairs; MAF = Minor allele frequency; FDR = False discovery rate | | | | | | | |

| **Table S3 Identities, positions, and effects of significantly associated additive SNPs obtained by single SNP regression mixed model for growth and carcass traits in combined population of beef cattle.** | | | | | | | |
| --- | --- | --- | --- | --- | --- | --- | --- |
| **Trait^1^** | **SNP reference** | **BTA** | **Position (bp)** | **MAF** | ***P*-value** | **FDR (%)** | **Allele substitution effects** |
| BWT, Kg | rs41585810 | 1 | 100,382,840 | 0.182 | 0.000146 | 10 | 0.658±0.173 |
|  | rs43111100 | 1 | 120,326,805 | 0.153 | 0.000132 | 10 | -0.854±0.223 |
|  | rs41625164 | 1 | 152,592,454 | 0.423 | 0.000178 | 10 | 0.418±0.111 |
|  | rs110241585 | 2 | 31,242,327 | 0.477 | 5.37E-05 | 5 | 0.450±0.111 |
|  | rs110423687 | 3 | 8,945,826 | 0.413 | 1.54E-05 | 5 | 0.490±0.113 |
|  | rs41575772 | 3 | 11,563,040 | 0.185 | 0.000161 | 10 | -0.727±0.193 |
|  | rs109909556 | 3 | 31,302,749 | 0.126 | 9.84E-05 | 10 | -1.010±0.259 |
|  | rs43712268 | 3 | 43,693,273 | 0.416 | 0.000172 | 10 | -0.413±0.110 |
|  | rs43339424 | 3 | 48,349,888 | 0.302 | 0.000101 | 10 | 0.519±0.133 |
|  | rs43339412 | 3 | 48392,443 | 0.280 | 2.95E-05 | 5 | 0.580±0.139 |
|  | rs109623041 | 3 | 49,681,867 | 0.435 | 9.64E-05 | 10 | 0.426±0.109 |
|  | rs29023590 | 3 | 52,943,988 | 0.222 | 0.000163 | 10 | -0.618±0.164 |
|  | rs110245482 | 4 | 105,359,174 | 0.496 | 4.35E-05 | 5 | -0.431±0.105 |
|  | rs110418487 | 4 | 119,961,092 | 0.271 | 1.00E-04 | 10 | -0.564±0.145 |
|  | rs41649876 | 6 | 27,433,375 | 0.450 | 1.80E-05 | 5 | 0.468±0.109 |
|  | rs41597449 | 6 | 28,099,293 | 0.292 | 0.000142 | 10 | 0.510±0.134 |
|  | rs41660811 | 6 | 28,181,355 | 0.229 | 2.56E-05 | 5 | 0.664±0.158 |
|  | rs41620119 | 6 | 28,213,461 | 0.233 | 1.34E-05 | 5 | -0.669±0.153 |
|  | rs29012331 | 6 | 31,067,604 | 0.387 | 1.30E-07 | 5 | -0.609±0.115 |
|  | rs29025673 | 6 | 31,126,062 | 0.293 | 0.000254 | 10 | -0.486±0.133 |
|  | rs41622316 | 6 | 31,275,687 | 0.198 | 1.00E-09 | 5 | -1.055±0.178 |
|  | rs109831465 | 6 | 32,805,721 | 0.169 | 3.86E-06 | 5 | 0.876±0.189 |
|  | rs109704656 | 6 | 36,062,803 | 0.350 | 0.000228 | 10 | 0.433±0.117 |
|  | rs110332219 | 6 | 36,986,502 | 0.400 | 5.78E-05 | 5 | 0.450±0.112 |
|  | rs110587419 | 6 | 37,019,972 | 0.341 | 4.15E-06 | 5 | 0.570±0.124 |
|  | rs110767541 | 6 | 37,104,193 | 0.453 | 5.90E-05 | 5 | 0.440±0.109 |
|  | rs41627896 | 6 | 37,218,883 | 0.354 | 5.00E-08 | 5 | 0.681±0.125 |
|  | rs109998457 | 6 | 37,252,345 | 0.348 | 1.80E-07 | 5 | 0.661±0.126 |
|  | rs109514983 | 6 | 37,584,088 | 0.239 | 9.00E-08 | 5 | -0.766±0.143 |
|  | rs109641632 | 6 | 37,653,391 | 0.167 | 7.48E-05 | 5 | -0.738±0.186 |
|  | rs41650794 | 6 | 37,839,427 | 0.487 | 6.56E-06 | 5 | -0.491±0.109 |
|  | rs110909199 | 6 | 37,868,743 | 0.477 | 1.00E-09 | 5 | 0.679±0.110 |
|  | rs29010895 | 6 | 38,042,011 | 0.493 | 1.00E-09 | 5 | -0.647±0.110 |
|  | rs111018320 | 6 | 38,133,743 | 0.411 | 7.00E-08 | 5 | 0.610±0.113 |
|  | rs110668054 | 6 | 38,464,203 | 0.359 | 1.00E-09 | 5 | 0.797±0.118 |
|  | rs109315366 | 6 | 38,576,012 | 0.416 | 1.00E-09 | 5 | -1.144±0.115 |
|  | rs41569339 | 6 | 38,689,886 | 0.258 | 0.000149 | 10 | 0.480±0.127 |
|  | rs109795992 | 6 | 38,825,835 | 0.471 | 1.00E-09 | 5 | -0.835±0.111 |
|  | rs110961068 | 6 | 38,845,992 | 0.464 | 1.00E-09 | 5 | 0.803±0.112 |
|  | rs109294917 | 6 | 38,869,785 | 0.482 | 1.00E-09 | 5 | 0.991±0.112 |
|  | rs109975627 | 6 | 38,914,175 | 0.130 | 0.000199 | 10 | 0.852±0.229 |
|  | rs110834363 | 6 | 38,939,012 | 0.434 | 1.00E-09 | 5 | 1.205±0.115 |
|  | rs110335851 | 6 | 39,034,201 | 0.254 | 0.000178 | 10 | -0.546±0.146 |
|  | rs110430755 | 6 | 39,094,340 | 0.143 | 0.000241 | 10 | 0.790±0.215 |
|  | rs110856492 | 6 | 39,114,894 | 0.143 | 0.000238 | 10 | -0.790±0.215 |
|  | rs43027303 | 6 | 39,172,862 | 0.257 | 8.82E-06 | 5 | 0.625±0.141 |
|  | rs110240246 | 6 | 39,257,620 | 0.452 | 1.00E-09 | 5 | 1.062±0.116 |
|  | rs110012183 | 6 | 39,346,170 | 0.443 | 1.00E-09 | 5 | 0.954±0.113 |
|  | rs110517212 | 6 | 39,371,150 | 0.346 | 1.00E-09 | 5 | 0.711±0.119 |
|  | rs42447219 | 6 | 39,438,580 | 0.186 | 0.000168 | 10 | 0.652±0.173 |
|  | rs110392766 | 6 | 39,529,973 | 0.498 | 3.24E-05 | 5 | 0.448±0.108 |
|  | rs109692323 | 6 | 39,597,740 | 0.324 | 5.97E-06 | 5 | 0.569±0.126 |
|  | rs109090869 | 6 | 39,752,891 | 0.433 | 1.00E-09 | 5 | -0.786±0.110 |
|  | rs109567582 | 6 | 39,816,133 | 0.440 | 9.39E-05 | 10 | -0.425±0.109 |
|  | rs43458270 | 6 | 39,837,065 | 0.279 | 1.00E-09 | 5 | 0.869±0.139 |
|  | rs43467099 | 6 | 40,063,618 | 0.307 | 8.90E-07 | 5 | 0.643±0.131 |
|  | rs109258862 | 6 | 40,629,318 | 0.375 | 1.00E-09 | 5 | 0.857±0.117 |
|  | rs42521925 | 6 | 40,893,067 | 0.493 | 8.91E-06 | 5 | -0.487±0.11 |
|  | rs109862934 | 6 | 41,588,847 | 0.257 | 4.62E-06 | 5 | 0.669±0.146 |
|  | rs43457653 | 6 | 41,914,851 | 0.479 | 1.06E-05 | 5 | -0.471±0.107 |
|  | rs43459808 | 6 | 42,057,261 | 0.461 | 1.20E-07 | 5 | 0.576±0.109 |
|  | rs43464875 | 6 | 42,446,118 | 0.192 | 0.000159 | 10 | 0.685±0.181 |
|  | rs43462203 | 6 | 42,567,228 | 0.231 | 1.93E-06 | 5 | -0.789±0.166 |
|  | rs41651273 | 6 | 42,655,795 | 0.306 | 0.000167 | 10 | -0.513±0.136 |
|  | rs41573706 | 6 | 43,037,439 | 0.439 | 0.000235 | 10 | -0.409±0.111 |
|  | rs109683796 | 6 | 45,479,538 | 0.319 | 8.20E-07 | 5 | -0.610±0.124 |
|  | rs29025709 | 6 | 46,260,375 | 0.368 | 6.52E-06 | 5 | 0.525±0.116 |
|  | rs109055778 | 6 | 46,599,570 | 0.499 | 3.40E-07 | 5 | -0.574±0.112 |
|  | rs109550729 | 6 | 46,820,055 | 0.302 | 0.000201 | 10 | -0.494±0.133 |
|  | rs110810914 | 6 | 87,281,196 | 0.264 | 9.59E-06 | 5 | 0.620±0.140 |
|  | rs109477453 | 7 | 82,762,865 | 0.427 | 0.00012 | 10 | -0.437±0.114 |
|  | rs41763830 | 7 | 87,359,924 | 0.119 | 0.000222 | 10 | -1.004±0.272 |
|  | rs109977037 | 7 | 90,900,133 | 0.263 | 0.000106 | 10 | -0.576±0.149 |
|  | rs109769526 | 7 | 91,693,979 | 0.414 | 5.18E-05 | 5 | -0.457±0.113 |
|  | rs41625107 | 7 | 91,762,948 | 0.452 | 2.47E-05 | 5 | 0.476±0.113 |
|  | rs109819349 | 7 | 9,300,435 | 0.324 | 1.00E-09 | 5 | -0.836±0.131 |
|  | rs41625563 | 7 | 93,073,890 | 0.479 | 2.00E-08 | 5 | -0.641±0.114 |
|  | rs110059753 | 7 | 93,218,452 | 0.420 | 1.00E-09 | 5 | -0.786±0.117 |
|  | rs41625576 | 7 | 93,289,032 | 0.185 | 4.59E-05 | 5 | -0.757±0.186 |
|  | rs109541071 | 8 | 25,668,823 | 0.100 | 0.000166 | 10 | 1.144±0.304 |
|  | rs41570400 | 10 | 99,626,471 | 0.376 | 5.60E-05 | 5 | -0.468±0.116 |
|  | rs41614761 | 11 | 84,083,499 | 0.453 | 0.000261 | 10 | 0.404±0.111 |
|  | rs110363085 | 12 | 40,009,119 | 0.305 | 0.000104 | 10 | -0.503±0.13 |
|  | rs109075576 | 13 | 2,011,011 | 0.291 | 4.88E-05 | 5 | -0.554±0.136 |
|  | rs110765746 | 13 | 57,524,735 | 0.119 | 1.68E-05 | 5 | 1.137±0.264 |
|  | rs110712904 | 13 | 75,742,150 | 0.423 | 0.000255 | 10 | 0.402±0.110 |
|  | rs42218359 | 14 | 7,342,696 | 0.422 | 0.00023 | 10 | 0.405±0.110 |
|  | rs110845517 | 14 | 16,967,274 | 0.174 | 6.32E-05 | 5 | -0.784±0.196 |
|  | rs42649775 | 14 | 24,437,778 | 0.165 | 1.00E-05 | 5 | -0.920±0.208 |
|  | rs42646660 | 14 | 24,524,205 | 0.144 | 3.00E-08 | 5 | -1.272±0.229 |
|  | rs42646708 | 14 | 24,573,257 | 0.137 | 3.10E-06 | 5 | 1.132±0.243 |
|  | rs41724332 | 14 | 24,643,266 | 0.120 | 0.000139 | 10 | -1.109±0.291 |
|  | rs29021334 | 14 | 25,612,510 | 0.204 | 0.000137 | 10 | -0.643±0.169 |
|  | rs110774011 | 14 | 25,698,286 | 0.451 | 0.00012 | 10 | 0.436±0.113 |
|  | rs42304759 | 14 | 26,196,375 | 0.488 | 5.75E-05 | 5 | 0.452±0.112 |
|  | rs42303720 | 14 | 26,264,142 | 0.487 | 1.88E-05 | 5 | -0.48±0.112 |
|  | rs41724601 | 14 | 26,713,734 | 0.466 | 0.000214 | 10 | -0.413±0.112 |
|  | rs41724536 | 14 | 26,766,010 | 0.443 | 0.000158 | 10 | -0.431±0.114 |
|  | rs42404949 | 14 | 26,949,215 | 0.460 | 5.37E-05 | 5 | 0.466±0.115 |
|  | rs110932157 | 14 | 28,155,879 | 0.345 | 0.000213 | 10 | -0.456±0.123 |
|  | rs41606574 | 14 | 31,513,907 | 0.334 | 0.000246 | 10 | 0.455±0.124 |
|  | rs109363625 | 15 | 75,937,148 | 0.235 | 0.000184 | 10 | -0.571±0.153 |
|  | rs41636582 | 17 | 2,520,482 | 0.258 | 0.000122 | 10 | 0.555±0.144 |
|  | rs109874691 | 17 | 64,011,938 | 0.483 | 0.00026 | 10 | 0.398±0.109 |
|  | rs110113612 | 18 | 46,611,338 | 0.464 | 0.000156 | 10 | 0.455±0.12 |
|  | rs109932054 | 19 | 47,798,697 | 0.342 | 8.54E-05 | 10 | 0.492±0.125 |
|  | rs29015626 | 20 | 4,567,765 | 0.291 | 0.000157 | 10 | 0.479±0.127 |
|  | rs43350564 | 20 | 4,618,689 | 0.349 | 6.50E-07 | 5 | 0.594±0.119 |
|  | rs110708961 | 20 | 4,845,639 | 0.416 | 4.50E-07 | 5 | 0.554±0.11 |
|  | rs42661291 | 20 | 4,962,725 | 0.265 | 0.000192 | 10 | -0.556±0.149 |
|  | rs110168637 | 22 | 19,286,702 | 0.158 | 5.20E-05 | 5 | 0.865±0.214 |
|  | rs110264113 | 22 | 55,511,639 | 0.398 | 9.69E-05 | 10 | -0.434±0.111 |
|  | rs41572498 | 22 | 55,586,234 | 0.384 | 5.90E-05 | 5 | 0.458±0.114 |
|  | rs110561429 | 25 | 10,896,067 | 0.490 | 8.49E-05 | 10 | -0.414±0.105 |
|  | rs110130856 | 25 | 38,330,924 | 0.348 | 0.000268 | 10 | -0.438±0.12 |
|  | rs110422161 | 27 | 21,940,197 | 0.209 | 0.0002 | 10 | 0.628±0.169 |
| WWT, Kg | rs29012836 | 6 | 35,648,890 | 0.167 | 2.72E-05 | 10 | -3.961±0.943 |
|  | rs29013268 | 6 | 35,788,711 | 0.187 | 7.40E-07 | 5 | 4.185±0.844 |
|  | rs111018320 | 6 | 38,133,743 | 0.411 | 1.00E-09 | 5 | 3.277±0.498 |
|  | rs110668054 | 6 | 38,464,203 | 0.359 | 3.93E-06 | 5 | 2.444±0.529 |
|  | rs109315366 | 6 | 38,576,012 | 0.416 | 1.00E-09 | 5 | -4.272±0.512 |
|  | rs109795992 | 6 | 38,825,835 | 0.471 | 2.24E-05 | 10 | -2.108±0.497 |
|  | rs109294917 | 6 | 38,869,785 | 0.482 | 1.60E-07 | 5 | 2.626±0.501 |
|  | rs110834363 | 6 | 38,939,012 | 0.434 | 1.00E-09 | 5 | 3.335±0.516 |
|  | rs110240246 | 6 | 39,257,620 | 0.452 | 3.28E-06 | 5 | 2.387±0.513 |
|  | rs110517212 | 6 | 39,371,150 | 0.346 | 1.14E-05 | 5 | 2.334±0.531 |
|  | rs109090869 | 6 | 39,752,891 | 0.433 | 3.08E-06 | 5 | -2.292±0.491 |
|  | rs109965915 | 7 | 53,859,609 | 0.324 | 1.19E-05 | 5 | -2.447±0.558 |
|  | rs41594575 | 7 | 53,907,954 | 0.320 | 5.23E-06 | 5 | 2.567±0.563 |
|  | rs110915503 | 7 | 53,932,886 | 0.323 | 1.34E-05 | 5 | -2.431±0.558 |
|  | rs109966288 | 7 | 62,664,915 | 0.261 | 2.06E-06 | 5 | -3.040±0.640 |
|  | rs109819349 | 7 | 93,007,435 | 0.324 | 3.49E-06 | 5 | -2.762±0.595 |
|  | rs110059753 | 7 | 93,218,452 | 0.420 | 1.20E-06 | 5 | -2.526±0.520 |
|  | rs110071409 | 9 | 56,616,446 | 0.363 | 2.99E-05 | 10 | 2.202±0.527 |
|  | rs109394917 | 14 | 40,684,301 | 0.337 | 1.74E-05 | 5 | -2.327±0.541 |
|  | rs110430859 | 15 | 8,495,973 | 0.231 | 5.33E-05 | 10 | 2.885±0.714 |
|  | rs41637950 | 19 | 54,756,421 | 0.385 | 3.54E-05 | 10 | -2.121±0.513 |
|  | rs43350564 | 20 | 4,618,689 | 0.349 | 1.01E-06 | 5 | 2.616±0.534 |
|  | rs42351264 | 20 | 7,296,768 | 0.460 | 4.15E-05 | 10 | 2.064±0.503 |
|  | rs109569668 | 22 | 43,478,537 | 0.439 | 5.51E-06 | 5 | -2.231±0.49 |
| PDG, Kg/d | rs111018320 | 6 | 38,133,743 | 0.411 | 1.00E-08 | 5 | 0.016±0.003 |
|  | rs109315366 | 6 | 38,576,012 | 0.416 | 1.00E-08 | 5 | -0.018±0.003 |
|  | rs110834363 | 6 | 38,939,012 | 0.434 | 5.76E-06 | 10 | 0.013±0.003 |
| ADG, Kg/d | rs41655687 | 4 | 63,906,905 | 0.276 | 0.000111 | 10 | 0.022±0.006 |
|  | rs43415920 | 4 | 113,506,092 | 0.248 | 1.59E-05 | 5 | 0.027±0.006 |
|  | rs43416912 | 4 | 113,532,717 | 0.249 | 1.20E-05 | 5 | 0.027±0.006 |
|  | rs41654528 | 5 | 106,230,591 | 0.219 | 5.08E-05 | 10 | -0.030±0.007 |
|  | rs41665871 | 6 | 35,611,267 | 0.466 | 7.96E-05 | 10 | 0.018±0.004 |
|  | rs110587419 | 6 | 37,019,972 | 0.341 | 1.13E-06 | 5 | 0.025±0.005 |
|  | rs41595968 | 6 | 37,463,048 | 0.255 | 6.67E-06 | 5 | 0.027±0.006 |
|  | rs110909199 | 6 | 37,868,743 | 0.477 | 1.24E-05 | 5 | 0.020±0.005 |
|  | rs29010895 | 6 | 38,042,011 | 0.493 | 1.60E-07 | 5 | -0.024±0.004 |
|  | rs111018320 | 6 | 38,133,743 | 0.411 | 5.34E-06 | 5 | 0.021±0.005 |
|  | rs110668054 | 6 | 38,464,203 | 0.359 | 1.00E-08 | 5 | 0.028±0.005 |
|  | rs109315366 | 6 | 38,576,012 | 0.416 | 1.00E-08 | 5 | -0.044±0.005 |
|  | rs109795992 | 6 | 38,825,835 | 0.471 | 1.00E-08 | 5 | -0.034±0.005 |
|  | rs110961068 | 6 | 38,845,992 | 0.464 | 1.00E-08 | 5 | 0.033±0.005 |
|  | rs109294917 | 6 | 38,869,785 | 0.482 | 1.00E-08 | 5 | 0.038±0.005 |
|  | rs110834363 | 6 | 38,939,012 | 0.434 | 1.00E-08 | 5 | 0.047±0.005 |
|  | rs110335851 | 6 | 39,034,201 | 0.254 | 4.09E-06 | 5 | -0.028±0.006 |
|  | rs110240246 | 6 | 39,257,620 | 0.452 | 1.00E-08 | 5 | 0.040±0.005 |
|  | rs110012183 | 6 | 39,346,170 | 0.443 | 1.00E-08 | 5 | 0.038±0.005 |
|  | rs109090869 | 6 | 39,752,891 | 0.433 | 2.00E-08 | 5 | -0.025±0.005 |
|  | rs109258862 | 6 | 40,629,318 | 0.375 | 1.11E-05 | 5 | 0.021±0.005 |
|  | rs43461214 | 6 | 41,178,449 | 0.421 | 4.14E-05 | 5 | 0.019±0.005 |
|  | rs43457653 | 6 | 41,914,851 | 0.479 | 3.93E-06 | 5 | -0.020±0.004 |
|  | rs41651258 | 6 | 42,023,749 | 0.228 | 4.55E-05 | 10 | 0.026±0.006 |
|  | rs43459808 | 6 | 42,057,261 | 0.461 | 1.06E-06 | 5 | 0.022±0.004 |
|  | rs43464875 | 6 | 42,446,118 | 0.192 | 2.32E-05 | 5 | 0.032±0.008 |
|  | rs43462203 | 6 | 42,567,228 | 0.231 | 4.95E-05 | 10 | -0.027±0.007 |
|  | rs41651273 | 6 | 42,655,795 | 0.306 | 2.35E-06 | 5 | -0.026±0.005 |
|  | rs109683796 | 6 | 45,479,538 | 0.319 | 4.42E-06 | 5 | -0.024±0.005 |
|  | rs29025709 | 6 | 46,260,375 | 0.368 | 1.00E-08 | 5 | 0.028±0.005 |
|  | rs43710090 | 6 | 46,481,458 | 0.491 | 8.08E-05 | 10 | -0.018±0.004 |
|  | rs110207017 | 6 | 46,533,015 | 0.446 | 1.04E-05 | 5 | -0.020±0.005 |
|  | rs109055778 | 6 | 46,599,570 | 0.499 | 1.02E-06 | 5 | -0.022±0.005 |
|  | rs41658480 | 6 | 54,237,782 | 0.464 | 5.39E-05 | 10 | -0.018±0.004 |
|  | rs109819349 | 7 | 93,007,435 | 0.324 | 5.00E-08 | 5 | -0.030±0.006 |
|  | rs41625563 | 7 | 93,073,890 | 0.479 | 2.50E-07 | 5 | -0.024±0.005 |
|  | rs110059753 | 7 | 93,218,452 | 0.420 | 1.00E-08 | 5 | -0.033±0.005 |
|  | rs109269005 | 11 | 67,347,867 | 0.165 | 8.79E-05 | 10 | -0.033±0.009 |
|  | rs109180881 | 11 | 67,862,943 | 0.121 | 3.08E-05 | 5 | 0.045±0.011 |
|  | rs41718711 | 12 | 65,662,261 | 0.251 | 8.03E-05 | 10 | 0.025±0.006 |
|  | rs42496826 | 13 | 45,276,113 | 0.229 | 3.39E-05 | 5 | -0.028±0.007 |
|  | rs110074594 | 13 | 52,982,280 | 0.115 | 4.95E-05 | 10 | 0.048±0.012 |
|  | rs110909859 | 13 | 63,688,976 | 0.478 | 5.68E-05 | 10 | -0.020±0.005 |
|  | rs42646660 | 14 | 24,524,205 | 0.144 | 2.40E-07 | 5 | -0.050±0.010 |
|  | rs42646708 | 14 | 24,573,257 | 0.137 | 2.82E-05 | 5 | 0.043±0.010 |
|  | rs41724385 | 14 | 24,607,527 | 0.127 | 0.00011 | 10 | -0.044±0.011 |
|  | rs29020544 | 18 | 43,327,273 | 0.415 | 6.37E-06 | 5 | 0.021±0.005 |
|  | rs109405571 | 20 | 4,238,552 | 0.364 | 3.12E-05 | 5 | 0.02±0.005 |
|  | rs43350564 | 20 | 4,618,689 | 0.349 | 4.13E-06 | 5 | 0.023±0.005 |
| YWT, Kg | rs29015773 | 1 | 8,998,063 | 0.470 | 0.000115 | 10 | 2.550±0.661 |
|  | rs41618226 | 1 | 40,379,850 | 0.398 | 0.000132 | 10 | -2.635±0.689 |
|  | rs43743867 | 2 | 13,375,340 | 0.311 | 0.000219 | 10 | -2.891±0.782 |
|  | rs110633489 | 2 | 14,717,851 | 0.428 | 9.78E-05 | 10 | -2.657±0.681 |
|  | rs111028820 | 2 | 24,773,731 | 0.364 | 0.000129 | 10 | 2.829±0.738 |
|  | rs109853660 | 2 | 106,549,887 | 0.140 | 0.00013 | 10 | -5.763±1.505 |
|  | rs110616281 | 3 | 73,229,266 | 0.446 | 0.000149 | 10 | -2.584±0.681 |
|  | rs43358795 | 3 | 97,560,648 | 0.150 | 0.000118 | 10 | 5.311±1.379 |
|  | rs41601710 | 3 | 105,467,351 | 0.321 | 1.77E-05 | 5 | 3.375±0.786 |
|  | rs43050734 | 5 | 864,491 | 0.437 | 1.26E-05 | 5 | 2.902±0.664 |
|  | rs41644577 | 6 | 26,706,330 | 0.405 | 4.53E-05 | 5 | -2.729±0.669 |
|  | rs110838417 | 6 | 29,709,875 | 0.206 | 0.000172 | 10 | 4.051±1.078 |
|  | rs41622316 | 6 | 31,275,687 | 0.198 | 4.65E-05 | 5 | -4.464±1.095 |
|  | rs110349978 | 6 | 32,128,224 | 0.128 | 0.000129 | 10 | 5.496±1.435 |
|  | rs41665871 | 6 | 35,611,267 | 0.466 | 0.000102 | 10 | 2.608±0.671 |
|  | rs29012836 | 6 | 35,648,890 | 0.167 | 1.30E-07 | 5 | -7.339±1.389 |
|  | rs29013268 | 6 | 35,788,711 | 0.187 | 1.00E-08 | 5 | 7.584±1.221 |
|  | rs41592814 | 6 | 36,277,967 | 0.283 | 2.33E-05 | 5 | -3.634±0.858 |
|  | rs110332219 | 6 | 36,986,502 | 0.400 | 1.29E-05 | 5 | 2.993±0.686 |
|  | rs110587419 | 6 | 37,019,972 | 0.341 | 3.31E-06 | 5 | 3.516±0.755 |
|  | rs110483628 | 6 | 37,323,779 | 0.315 | 0.000104 | 10 | 3.077±0.792 |
|  | rs41605197 | 6 | 37,399,296 | 0.260 | 1.89E-05 | 5 | -3.702±0.865 |
|  | rs41595968 | 6 | 37,463,048 | 0.255 | 4.12E-06 | 5 | 4.056±0.880 |
|  | rs109514983 | 6 | 37,584,088 | 0.239 | 1.54E-06 | 5 | -4.281±0.890 |
|  | rs110909199 | 6 | 37,868,743 | 0.477 | 1.01E-05 | 5 | 2.985±0.675 |
|  | rs29010895 | 6 | 38,042,011 | 0.493 | 2.00E-08 | 5 | -3.788±0.674 |
|  | rs111018320 | 6 | 38,133,743 | 0.411 | 1.00E-08 | 5 | 5.407±0.688 |
|  | rs109248021 | 6 | 38,203,273 | 0.481 | 1.91E-05 | 5 | -2.843±0.665 |
|  | rs110668054 | 6 | 38,464,203 | 0.359 | 1.00E-08 | 5 | 5.679±0.734 |
|  | rs109315366 | 6 | 38,576,012 | 0.416 | 1.00E-08 | 5 | -7.941±0.707 |
|  | rs109795992 | 6 | 38,825,835 | 0.471 | 1.00E-08 | 5 | -4.284±0.682 |
|  | rs110961068 | 6 | 38,845,992 | 0.464 | 1.00E-08 | 5 | 4.021±0.685 |
|  | rs109294917 | 6 | 38,869,785 | 0.482 | 1.00E-08 | 5 | 5.500±0.688 |
|  | rs110834363 | 6 | 38,939,012 | 0.434 | 1.00E-08 | 5 | 6.712±0.709 |
|  | rs43027303 | 6 | 39,172,862 | 0.257 | 3.00E-08 | 5 | 4.866±0.873 |
|  | rs110240246 | 6 | 39,257,620 | 0.452 | 1.00E-08 | 5 | 5.035±0.707 |
|  | rs110012183 | 6 | 39,346,170 | 0.443 | 1.00E-08 | 5 | 4.543±0.692 |
|  | rs110517212 | 6 | 39,371,150 | 0.346 | 7.00E-08 | 5 | 3.957±0.733 |
|  | rs42447219 | 6 | 39,438,580 | 0.186 | 1.10E-05 | 5 | 4.838±1.100 |
|  | rs110392766 | 6 | 39,529,973 | 0.498 | 2.37E-06 | 5 | 3.129±0.662 |
|  | rs109692323 | 6 | 39,597,740 | 0.324 | 4.60E-05 | 5 | 3.177±0.779 |
|  | rs109090869 | 6 | 39,752,891 | 0.433 | 1.00E-08 | 5 | -4.272±0.681 |
|  | rs43458270 | 6 | 39,837,065 | 0.279 | 1.80E-07 | 5 | 4.534±0.867 |
|  | rs43467099 | 6 | 40,063,618 | 0.307 | 3.50E-07 | 5 | 4.021±0.788 |
|  | rs109258862 | 6 | 40,629,318 | 0.375 | 2.30E-07 | 5 | 3.718±0.718 |
|  | rs42521925 | 6 | 40,893,067 | 0.493 | 6.94E-05 | 5 | -2.702±0.679 |
|  | rs43457658 | 6 | 41,858,119 | 0.207 | 4.39E-05 | 5 | 4.370±1.069 |
|  | rs43457653 | 6 | 41,914,851 | 0.479 | 1.47E-05 | 5 | -2.855±0.658 |
|  | rs41573706 | 6 | 43,037,439 | 0.439 | 0.000121 | 10 | -2.605±0.677 |
|  | rs109054259 | 6 | 45,440,921 | 0.261 | 6.76E-05 | 5 | 3.555±0.892 |
|  | rs109683796 | 6 | 45,479,538 | 0.319 | 0.000132 | 10 | -2.958±0.773 |
|  | rs109055778 | 6 | 46,599,570 | 0.499 | 6.98E-05 | 5 | -2.735±0.687 |
|  | rs109550729 | 6 | 46,820,055 | 0.302 | 9.38E-05 | 10 | -3.199±0.818 |
|  | rs109325337 | 6 | 69,416,751 | 0.386 | 3.19E-05 | 5 | -3.228±0.776 |
|  | rs41591941 | 7 | 16,634,828 | 0.213 | 9.52E-05 | 10 | 4.019±1.029 |
|  | rs110323027 | 7 | 46,293,900 | 0.279 | 0.000165 | 10 | -3.179±0.843 |
|  | rs109769526 | 7 | 91,693,979 | 0.414 | 2.53E-06 | 5 | -3.245±0.689 |
|  | rs109819349 | 7 | 93,007,435 | 0.324 | 1.00E-08 | 5 | -4.698±0.819 |
|  | rs41625563 | 7 | 93,073,890 | 0.479 | 8.20E-07 | 5 | -3.433±0.696 |
|  | rs110059753 | 7 | 93,218,452 | 0.420 | 1.00E-08 | 5 | -4.364±0.714 |
|  | rs109976835 | 8 | 34,536,574 | 0.213 | 1.77E-05 | 5 | 4.618±1.075 |
|  | rs109832919 | 8 | 61,536,940 | 0.272 | 1.45E-05 | 5 | -3.671±0.846 |
|  | rs109942542 | 8 | 71,293,524 | 0.467 | 0.000139 | 10 | -2.550±0.669 |
|  | rs109761047 | 9 | 103,777,260 | 0.473 | 2.44E-06 | 5 | -3.141±0.666 |
|  | rs109560569 | 10 | 100,133,280 | 0.280 | 7.09E-05 | 5 | 3.329±0.837 |
|  | rs109733774 | 11 | 25,418,833 | 0.351 | 3.50E-05 | 5 | 3.138±0.758 |
|  | rs41569395 | 11 | 67,830,333 | 0.487 | 0.00022 | 10 | 2.506±0.678 |
|  | rs41612874 | 11 | 77,330,791 | 0.406 | 4.19E-05 | 5 | -2.834±0.691 |
|  | rs29021098 | 12 | 28,052,265 | 0.320 | 2.09E-06 | 5 | 3.811±0.802 |
|  | rs43702157 | 12 | 54,550,449 | 0.428 | 0.000102 | 10 | -2.639±0.679 |
|  | rs110508720 | 13 | 7,564,399 | 0.258 | 6.58E-05 | 5 | 3.633±0.910 |
|  | rs41631541 | 13 | 63,257,337 | 0.369 | 3.63E-05 | 5 | -2.953±0.714 |
|  | rs110383563 | 14 | 24,326,513 | 0.465 | 0.000168 | 10 | 2.683±0.712 |
|  | rs42649775 | 14 | 24,437,778 | 0.165 | 3.10E-07 | 5 | -6.536±1.276 |
|  | rs42646660 | 14 | 24,524,205 | 0.144 | 1.30E-07 | 5 | -7.474±1.415 |
|  | rs109661415 | 14 | 30,397,178 | 0.235 | 3.01E-05 | 5 | -4.055±0.971 |
|  | rs109394917 | 14 | 40,684,301 | 0.337 | 4.00E-07 | 5 | -3.821±0.753 |
|  | rs42221575 | 15 | 60,250,434 | 0.140 | 0.000162 | 10 | 5.496±1.456 |
|  | rs41603739 | 15 | 65,813,540 | 0.461 | 0.00012 | 10 | 2.625±0.682 |
|  | rs110163969 | 15 | 65,871,270 | 0.118 | 0.00019 | 10 | 5.989±1.603 |
|  | rs109046738 | 17 | 17,778,684 | 0.416 | 3.07E-05 | 5 | -2.947±0.707 |
|  | rs29015626 | 20 | 4,567,765 | 0.291 | 4.95E-05 | 5 | 3.261±0.803 |
|  | rs43350564 | 20 | 4,618,689 | 0.349 | 1.00E-08 | 5 | 4.662±0.737 |
|  | rs43349755 | 20 | 4,746,836 | 0.436 | 0.000111 | 10 | 2.688±0.695 |
|  | rs110708961 | 20 | 4,845,639 | 0.416 | 1.30E-06 | 5 | 3.263±0.674 |
|  | rs110492458 | 20 | 4,886,018 | 0.272 | 3.81E-05 | 5 | 3.591±0.871 |
|  | rs108971629 | 20 | 6,832,903 | 0.339 | 0.000178 | 10 | -2.830±0.755 |
|  | rs42351264 | 20 | 7,296,768 | 0.460 | 0.000156 | 10 | 2.624±0.694 |
|  | rs42356467 | 20 | 7,351,732 | 0.481 | 0.000187 | 10 | -2.526±0.676 |
|  | rs41664902 | 20 | 12,158,936 | 0.331 | 0.000107 | 10 | 3.011±0.777 |
|  | rs110175546 | 20 | 17,240,999 | 0.442 | 9.37E-06 | 5 | -2.974±0.671 |
|  | rs41648560 | 25 | 18,029,588 | 0.172 | 1.39E-05 | 5 | 5.413±1.244 |
|  | rs110282224 | 25 | 18,067,339 | 0.171 | 0.000149 | 10 | 4.777±1.258 |
|  | rs109914995 | 29 | 49,906,123 | 0.372 | 2.02E-05 | 5 | -3.209±0.752 |
| HCW, Kg | rs110110700 | 4 | 115,581,055 | 0.393 | 3.46E-05 | 10 | 2.808±0.677 |
|  | rs111018320 | 6 | 38,133,743 | 0.411 | 1.69E-06 | 5 | 3.237±0.675 |
|  | rs110668054 | 6 | 38,464,203 | 0.359 | 1.00E-08 | 5 | 4.559±0.733 |
|  | rs109315366 | 6 | 38,576,012 | 0.416 | 1.00E-08 | 5 | -4.982±0.680 |
|  | rs41569339 | 6 | 38,689,886 | 0.258 | 2.39E-05 | 5 | 3.419±0.808 |
|  | rs109795992 | 6 | 38,825,835 | 0.471 | 1.56E-06 | 5 | -3.257±0.677 |
|  | rs110961068 | 6 | 38,845,992 | 0.464 | 9.47E-06 | 5 | 3.038±0.685 |
|  | rs109294917 | 6 | 38,869,785 | 0.482 | 1.00E-08 | 5 | 4.244±0.670 |
|  | rs110834363 | 6 | 38,939,012 | 0.434 | 1.00E-08 | 5 | 5.589±0.686 |
|  | rs43027303 | 6 | 39,172,862 | 0.257 | 6.42E-05 | 10 | 3.589±0.897 |
|  | rs110240246 | 6 | 39,257,620 | 0.452 | 1.00E-08 | 5 | 4.475±0.703 |
|  | rs110012183 | 6 | 39,346,170 | 0.443 | 1.00E-08 | 5 | 4.370±0.678 |
|  | rs41650820 | 6 | 40,086,624 | 0.395 | 2.25E-05 | 5 | -2.774±0.654 |
|  | rs109258862 | 6 | 40,629,318 | 0.375 | 4.00E-08 | 5 | 3.817±0.692 |
|  | rs43461214 | 6 | 41,178,449 | 0.421 | 4.59E-05 | 10 | 2.720±0.667 |
|  | rs29025709 | 6 | 46,260,375 | 0.368 | 2.63E-06 | 5 | 3.294±0.700 |
|  | rs109055778 | 6 | 46,599,570 | 0.499 | 5.18E-05 | 10 | -2.659±0.656 |
|  | rs110357652 | 6 | 53,312,840 | 0.232 | 3.49E-05 | 10 | 3.887±0.938 |
|  | rs109769526 | 7 | 91,693,979 | 0.414 | 7.88E-06 | 5 | -3.073±0.687 |
|  | rs109819349 | 7 | 9,3007,435 | 0.324 | 1.00E-08 | 5 | -4.778±0.803 |
|  | rs41625563 | 7 | 93,073,890 | 0.479 | 1.18E-06 | 5 | -3.241±0.666 |
|  | rs110059753 | 7 | 93,218,452 | 0.420 | 1.00E-08 | 5 | -5.037±0.704 |
|  | rs42252297 | 8 | 18,638,310 | 0.385 | 1.11E-05 | 5 | -3.036±0.690 |
|  | rs43710056 | 8 | 71,874,533 | 0.454 | 9.52E-06 | 5 | 2.851±0.643 |
|  | rs43707538 | 10 | 36,374,166 | 0.457 | 4.55E-06 | 5 | 3.004±0.654 |
|  | rs42646660 | 14 | 24,524,205 | 0.144 | 1.00E-08 | 5 | -7.686±1.341 |
|  | rs42646708 | 14 | 24,573,257 | 0.137 | 7.64E-06 | 5 | 6.572±1.467 |
|  | rs29021868 | 14 | 31,014,368 | 0.436 | 1.17E-05 | 5 | -2.831±0.645 |
|  | rs29014028 | 22 | 60,084,013 | 0.387 | 2.16E-05 | 5 | 2.936±0.690 |
| FAT, mm | rs109315366 | 6 | 38,576,012 | 0.416 | 1.07E-06 | 5 | 0.422±0.086 |
|  | rs109795992 | 6 | 38,825,835 | 0.471 | 1.53E-05 | 10 | 0.371±0.086 |
|  | rs109294917 | 6 | 38,869,785 | 0.482 | 7.00E-08 | 5 | -0.458±0.085 |
|  | rs110834363 | 6 | 38,939,012 | 0.434 | 1.00E-08 | 5 | -0.517±0.087 |
|  | rs110240246 | 6 | 39,257,620 | 0.452 | 2.30E-07 | 5 | -0.462±0.089 |
|  | rs109090869 | 6 | 39,752,891 | 0.433 | 6.91E-06 | 5 | 0.376±0.083 |
|  | rs109991451 | 17 | 62,662,034 | 0.486 | 2.10E-07 | 5 | 0.416±0.080 |
|  | rs109389031 | 29 | 25,419,745 | 0.422 | 1.20E-05 | 10 | -0.368±0.084 |
| REA, cm | rs109558564 | 2 | 6,183,129 | 0.250 | 3.11E-05 | 10 | -1.176±0.282 |
|  | rs29010895 | 6 | 38,042,011 | 0.493 | 2.30E-05 | 10 | -0.898±0.212 |
|  | rs111018320 | 6 | 38,133,743 | 0.411 | 5.46E-06 | 5 | 0.999±0.220 |
|  | rs110668054 | 6 | 38,464,203 | 0.359 | 3.00E-08 | 5 | 1.317±0.238 |
|  | rs109315366 | 6 | 38,576,012 | 0.416 | 1.00E-08 | 5 | -1.421±0.220 |
|  | rs109294917 | 6 | 38,869,785 | 0.482 | 8.58E-06 | 5 | 0.971±0.218 |
|  | rs110834363 | 6 | 38,939,012 | 0.434 | 1.00E-08 | 5 | 1.371±0.223 |
|  | rs110240246 | 6 | 39,257,620 | 0.452 | 1.15E-06 | 5 | 1.117±0.229 |
|  | rs110012183 | 6 | 39,346,170 | 0.443 | 2.00E-08 | 5 | 1.239±0.221 |
|  | rs42521925 | 6 | 40,893,067 | 0.493 | 3.49E-06 | 5 | -0.986±0.212 |
|  | rs109769526 | 7 | 91,693,979 | 0.414 | 1.31E-05 | 5 | -0.975±0.224 |
|  | rs41625107 | 7 | 91,762,948 | 0.452 | 6.54E-06 | 5 | 0.977±0.216 |
|  | rs109819349 | 7 | 93,007,435 | 0.324 | 1.00E-08 | 5 | -1.666±0.261 |
|  | rs41625563 | 7 | 93,073,890 | 0.479 | 2.58E-06 | 5 | -1.020±0.217 |
|  | rs110059753 | 7 | 93,218,452 | 0.420 | 1.00E-08 | 5 | -1.555±0.229 |
|  | rs42699424 | 8 | 42,486,699 | 0.325 | 3.23E-05 | 10 | -1.056±0.254 |
|  | rs41567899 | 9 | 46,089,759 | 0.465 | 3.93E-06 | 5 | -0.987±0.214 |
|  | rs43705618 | 9 | 46,118,697 | 0.465 | 3.91E-06 | 5 | 0.988±0.214 |
|  | rs109118784 | 24 | 8,605,493 | 0.116 | 3.19E-05 | 10 | -2.264±0.544 |
| MBS | rs109819349 | 7 | 93,007,435 | 0.324 | 5.00E-08 | 5 | 10.970±2.011 |
|  | rs110059753 | 7 | 93,218,452 | 0.420 | 7.40E-07 | 5 | 8.782±1.771 |
|  | rs29009626 | 7 | 93,886,136 | 0.479 | 7.58E-06 | 10 | -7.561±1.687 |
|  | rs110559366 | 15 | 38,879,030 | 0.338 | 5.00E-08 | 5 | -10.240±1.879 |
| LMY, % | rs41645114 | 2 | 5,640,288 | 0.455 | 1.57E-05 | 10 | -0.343±0.079 |
|  | rs109315366 | 6 | 38,576,012 | 0.416 | 1.50E-07 | 5 | -0.442±0.084 |
|  | rs109294917 | 6 | 38,869,785 | 0.482 | 2.70E-06 | 5 | 0.389±0.083 |
|  | rs110834363 | 6 | 38,939,012 | 0.434 | 3.00E-08 | 5 | 0.470±0.085 |
|  | rs110240246 | 6 | 39,257,620 | 0.452 | 3.15E-06 | 5 | 0.406±0.087 |
|  | rs110012183 | 6 | 39,346,170 | 0.443 | 1.67E-05 | 10 | 0.362±0.084 |
|  | rs109090869 | 6 | 39,752,891 | 0.433 | 1.56E-05 | 10 | -0.352±0.081 |
|  | rs29019622 | 8 | 44,764,274 | 0.421 | 1.89E-05 | 10 | -0.348±0.081 |
|  | rs109151014 | 8 | 45,957,514 | 0.196 | 1.49E-06 | 5 | 0.644±0.134 |
|  | rs17870398 | 10 | 43,272,273 | 0.204 | 4.94E-06 | 5 | 0.586±0.128 |
|  | rs43649457 | 10 | 90,705,511 | 0.463 | 3.37E-06 | 5 | 0.371±0.080 |
|  | rs43711168 | 13 | 65,424,218 | 0.400 | 2.72E-05 | 10 | 0.381±0.091 |
|  | rs43046141 | 16 | 55,909,374 | 0.194 | 2.38E-05 | 10 | -0.570±0.135 |
|  | rs41603517 | 22 | 47,465,329 | 0.497 | 2.54E-05 | 10 | -0.329±0.078 |
| YG | rs109025109 | 2 | 5,601,419 | 0.499 | 1.27E-05 | 10 | 0.063±0.014 |
|  | rs41645114 | 2 | 5,640,288 | 0.455 | 1.74E-06 | 10 | 0.069±0.014 |
|  | rs109315366 | 6 | 38,576,012 | 0.416 | 4.19E-06 | 10 | 0.071±0.015 |
|  | rs110834363 | 6 | 38,939,012 | 0.434 | 1.30E-05 | 10 | -0.068±0.015 |
|  | rs17870398 | 10 | 43,272,273 | 0.204 | 3.15E-06 | 10 | -0.108±0.023 |
|  | rs43649457 | 10 | 90,705,511 | 0.463 | 1.44E-05 | 10 | -0.063±0.015 |
|  | rs110909859 | 13 | 63,688,976 | 0.478 | 1.27E-05 | 10 | -0.071±0.016 |
|  | rs43711168 | 13 | 65,424,218 | 0.400 | 1.28E-05 | 10 | -0.072±0.017 |
|  | rs43046141 | 16 | 55,909,374 | 0.194 | 1.08E-05 | 10 | 0.108±0.024 |
| ^1^Traits includes birth weight (BWT); weaning weight (WWT); pre-weaning daily gain (PDG); average daily gain (ADG); and yearling weight (YWT); hot carcass weight (HCW); back fat thickness (FAT); rib eye area (REA); marbling score (MBS); lean meat yield (LMY) and yield grade (YG)  BTA = *Bos taurus autosome*; bp = Base pairs; MAF = Minor allele frequency; FDR = False discovery rate | | | | | | | |
