# Supplementary material for: Genome-wide association scan for heterotic quantitative trait loci in multi-breed and crossbred beef cattle
Source: Genet Sel Evol. 2018 Oct 5;50:48. doi: 10.1186/s12711-018-0405-y (PMC6173862; doi:10.1186/s12711-018-0405-y)
Supplement: Supplementary file 2 — Additional file 2: Figure S1. Quantile–quantile (Q–Q) plot of p values for additive SNP association with growth traits in purebreds. Figure S2. Quantile–quantile (Q–Q) plot of p values for additive SNP association with carcass traits in purebreds. Figure S3. Quantile–quantile (Q–Q) plot of p values for additive SNP association with growth traits in crossbreds. Figure S4. Quantile–quantile (Q–Q) plot of p values for additive SNP association with carcass traits in crossbreds. Figure S5. Quantile–quantile (Q–Q) plot of p values for additive SNP association with growth traits in combined data. Figure S6. Quantile–quantile (Q–Q) plot of p values for additive SNP association with carcass traits in combined data. Figure S7. Quantile–quantile (Q–Q) plot of p values for dominance SNP association with growth traits in purebreds. Figure S8. Quantile–quantile (Q–Q) plot of p values for dominance SNP association with carcass traits in purebreds. Figure S9. Quantile–quantile (Q–Q) plot of p values for dominance SNP association with growth traits in crossbreds. Figure S10. Quantile–quantile (Q–Q) plot of p values for dominance SNP association with carcass traits in crossbreds. Figure S11. Quantile–quantile (Q–Q) plot of p values for dominance SNP association with growth traits in the combined data. Figure S12. Quantile–quantile (Q–Q) plot of p values for dominance SNP association with carcass traits in the combined data. Figure S13. Joint genome-wide association analysis for additive SNP effects on growth traits in purebreds. Figure S14. Joint genome-wide association analysis for additive SNP effects on carcass traits in purebreds. Figure S15. Joint genome-wide association analysis for additive SNP effects on growth traits in crossbreds. Figure S16. Joint genome-wide association analysis for additive SNP effects on carcass traits in crossbreds. Figure S17. Joint genome-wide association analysis for additive SNP effects on growth traits in the combined data. Figure S18. Joint genom [file 12711_2018_405_MOESM2_ESM.docx]

| 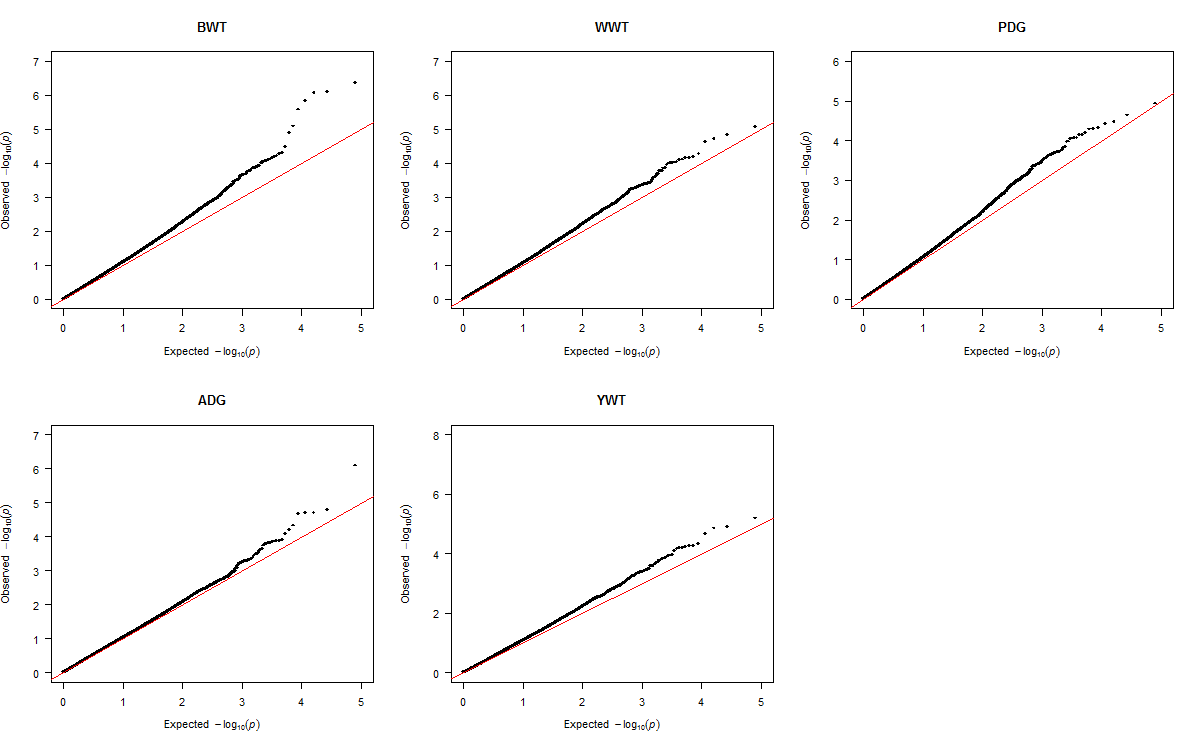 |
| --- |
| **Figure S1 Quantile-quantile (Q-Q) plot of *p*-values for additive SNP association with growth traits in Purebreds.** Purebred group included individuals with > 80% of Angus, Hereford and Charolais breed proportions, respectively. The traits are birth weight (BWT); weaning weight (WWT); pre-weaning daily gain (PDG); average daily gain (ADG); and yearling weight (YWT). The red line denotes the expected distribution under the null hypothesis of no significant association. Deviations between the red line and black dots indicate how the test statistics of SNPs deviates from the null hypothesis |

| 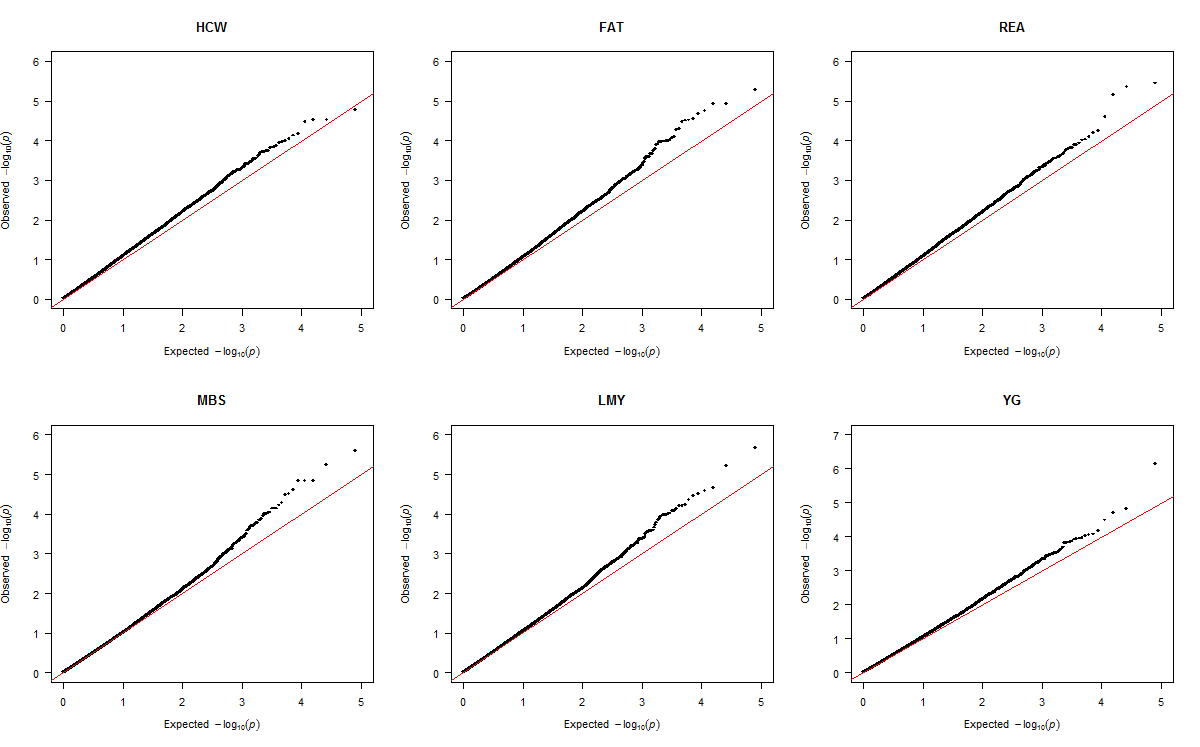 |
| --- |
| **Figure S2 Quantile-quantile (Q-Q) plot of *p*-values for additive SNP association with carcass traits in Purebreds.** Purebred group included individuals with > 80% of Angus, Hereford and Charolais breed proportions, respectively. The traits are hot carcass weight (HCW); back fat thickness (FAT); rib eye area (REA); marbling score (MBS); lean meat yield (LMY) and yield grade (YG). The red line denotes the expected distribution under the null hypothesis of no significant association. Deviations between the red line and black dots indicate how the test statistics of SNPs deviates from the null hypothesis |

| 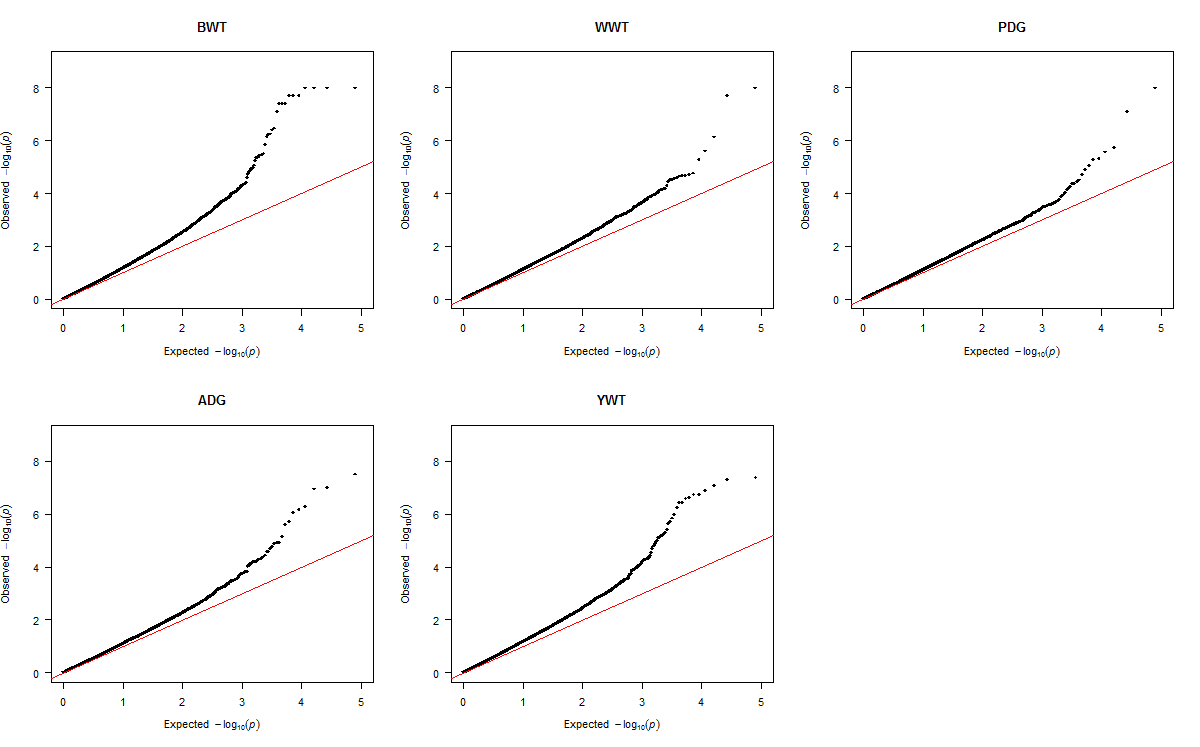 |
| --- |
| **Figure S3 Quantile-quantile (Q-Q) plot of *p*-values for additive SNP association with growth traits in Crossbreds.** Crossbred group included Kinsella composite, Beefbooster TX composite (www.beefbooster.com) and two and more way crosses involving Angus, Hereford, Charolais, Gelbvieh, Simmental, Limousin, and Piedmontese breeds. The traits are birth weight (BWT); weaning weight (WWT); pre-weaning daily gain (PDG); average daily gain (ADG); and yearling weight (YWT). The red line denotes the expected distribution under the null hypothesis of no significant association. Deviations between the red line and black dots indicate how the test statistics of SNPs deviates from the null hypothesis |

| 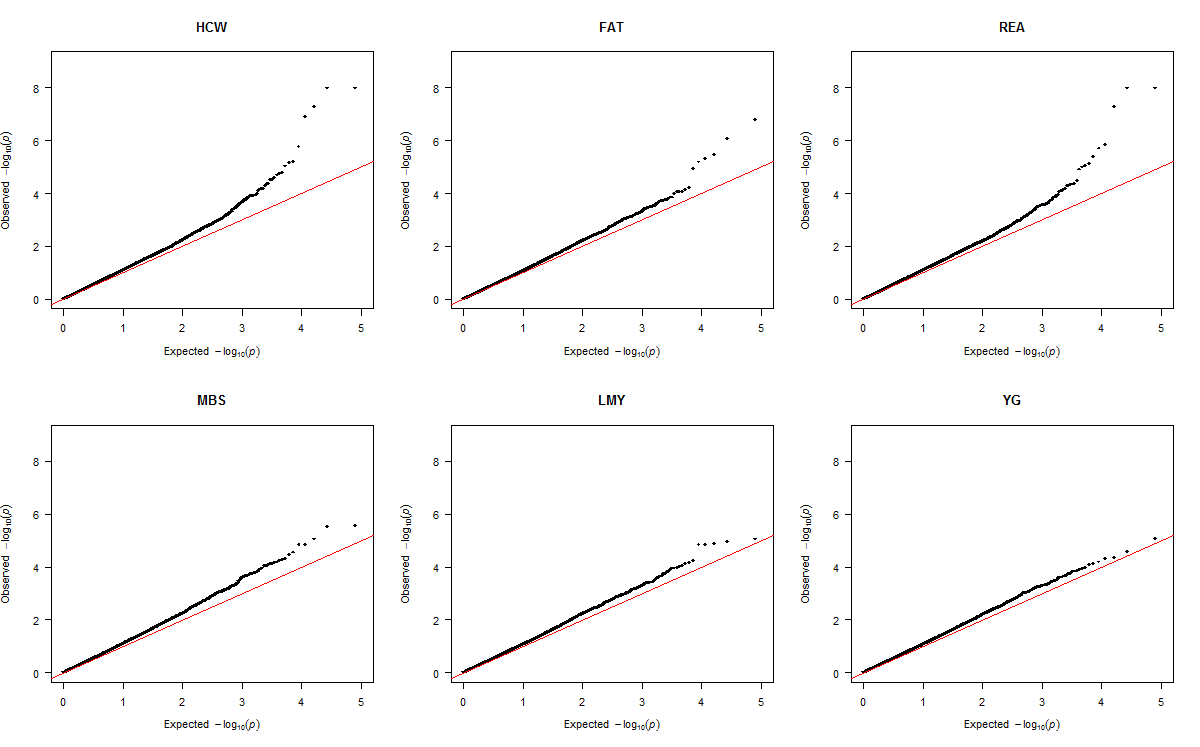 |
| --- |
| **Figure S4 Quantile-quantile (Q-Q) plot of *p*-values for additive SNP association with carcass traits in Crossbreds.** Crossbred group included Kinsella composite, Beefbooster TX composite (www.beefbooster.com) and two and more way crosses involving Angus, Hereford, Charolais, Gelbvieh, Simmental, Limousin, and Piedmontese breeds. The traits are hot carcass weight (HCW); back fat thickness (FAT); rib eye area (REA); marbling score (MBS); lean meat yield (LMY) and yield grade (YG). The red line denotes the expected distribution under the null hypothesis of no significant association. Deviations between the red line and black dots indicate how the test statistics of SNPs deviates from the null hypothesis |

| 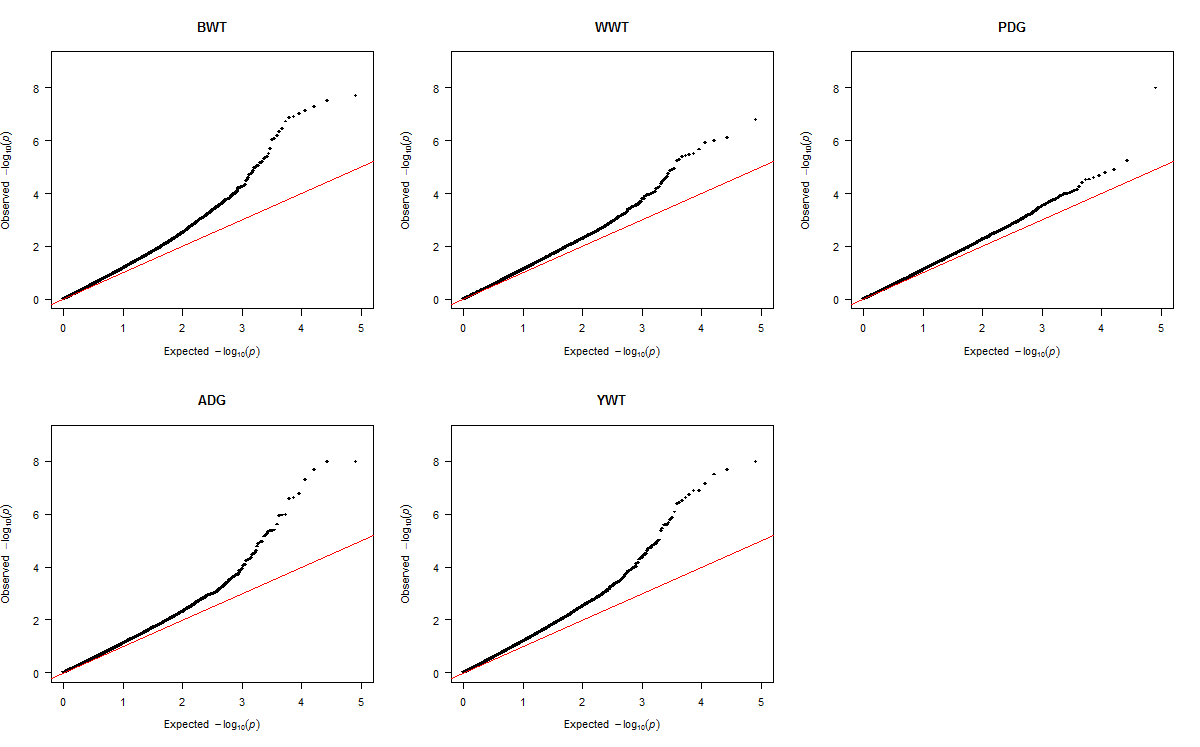 |
| --- |
| **Figure S5 Quantile-quantile (Q-Q) plot of *p*-values for additive SNP association with growth traits in combined data.** The traits are birth weight (BWT); weaning weight (WWT); pre-weaning daily gain (PDG); average daily gain (ADG); and yearling weight (YWT). The red line denotes the expected distribution under the null hypothesis of no significant association. Deviations between the red line and black dots indicate how the test statistics of SNPs deviates from the null hypothesis |

| 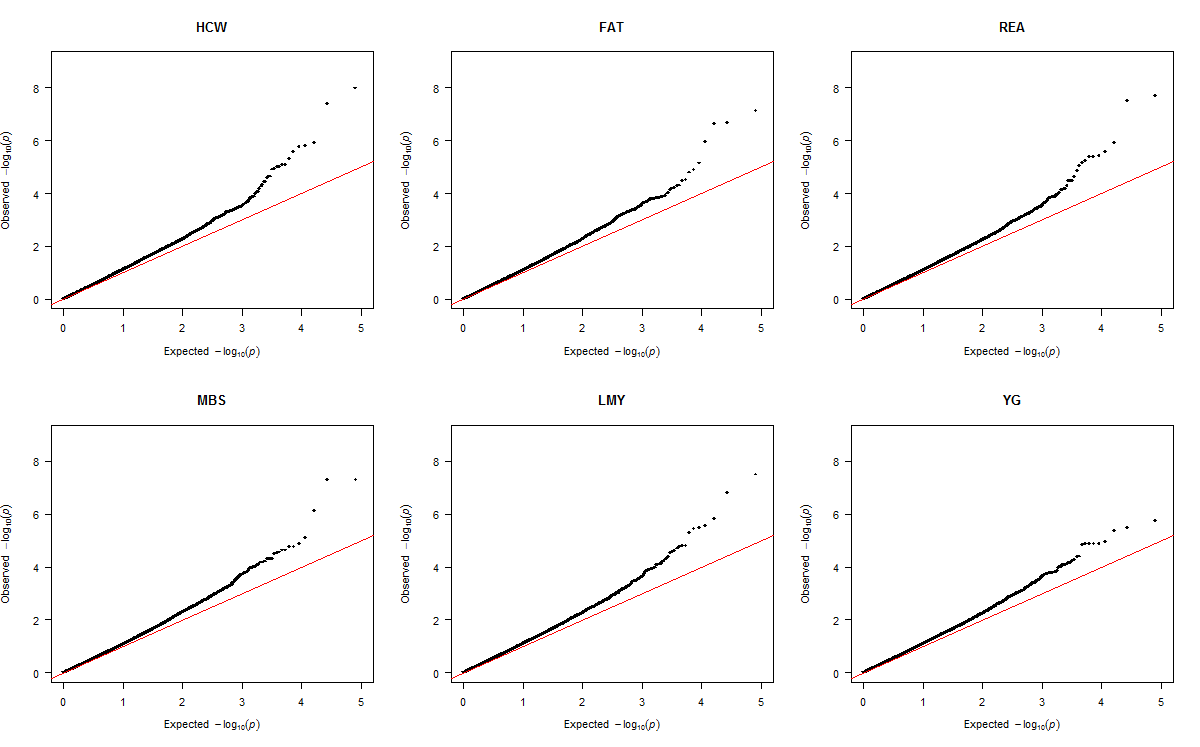 |
| --- |
| **Figure S6 Quantile-quantile (Q-Q) plot of *p*-values for additive SNP association with carcass traits in combined data.** The traits are hot carcass weight (HCW); back fat thickness (FAT); rib eye area (REA); marbling score (MBS); lean meat yield (LMY) and yield grade (YG). The red line denotes the expected distribution under the null hypothesis of no significant association. Deviations between the red line and black dots indicate how the test statistics of SNPs deviates from the null hypothesis |

| 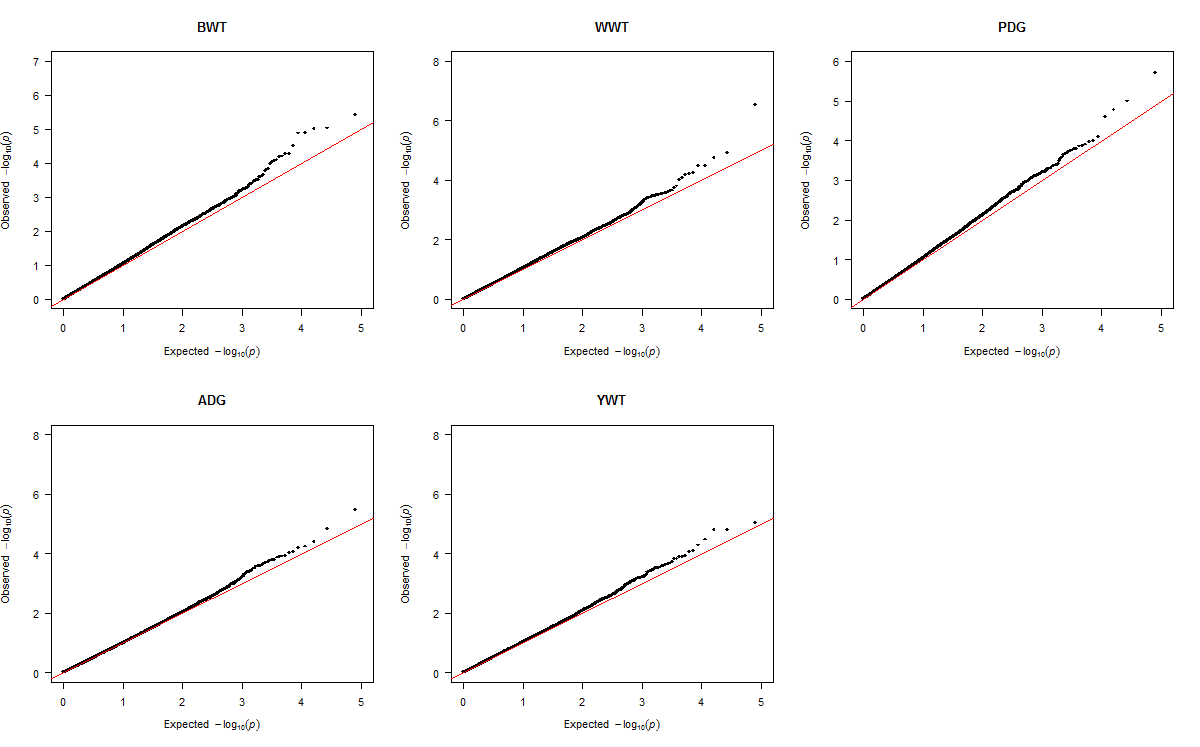 |
| --- |
| **Figure S7 Quantile-quantile (Q-Q) plot of *p*-values for dominance SNP association with growth traits in Purebreds.** Purebred group included individuals with > 80% of Angus, Hereford and Charolais breed proportions, respectively. The traits are birth weight (BWT); weaning weight (WWT); pre-weaning daily gain (PDG); average daily gain (ADG); and yearling weight (YWT). The red line denotes the expected distribution under the null hypothesis of no significant association. Deviations between the red line and black dots indicate how the test statistics of SNPs deviates from the null hypothesis |

| 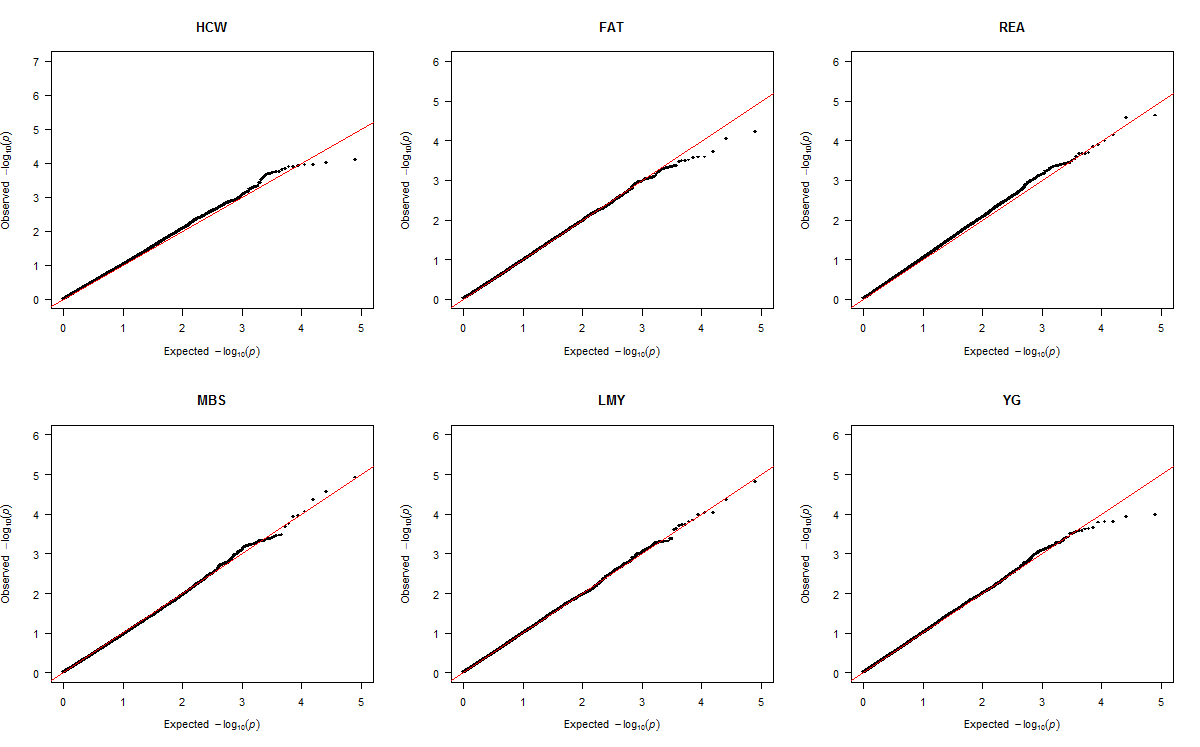 |
| --- |
| **Figure S8 Quantile-quantile (Q-Q) plot of *p*-values for dominance SNP association with carcass traits in Purebreds.** Purebred group included individuals with > 80% of Angus, Hereford and Charolais breed proportions, respectively. The traits are hot carcass weight (HCW); back fat thickness (FAT); rib eye area (REA); marbling score (MBS); lean meat yield (LMY) and yield grade (YG). The red line denotes the expected distribution under the null hypothesis of no significant association. Deviations between the red line and black dots indicate how the test statistics of SNPs deviates from the null hypothesis |

| 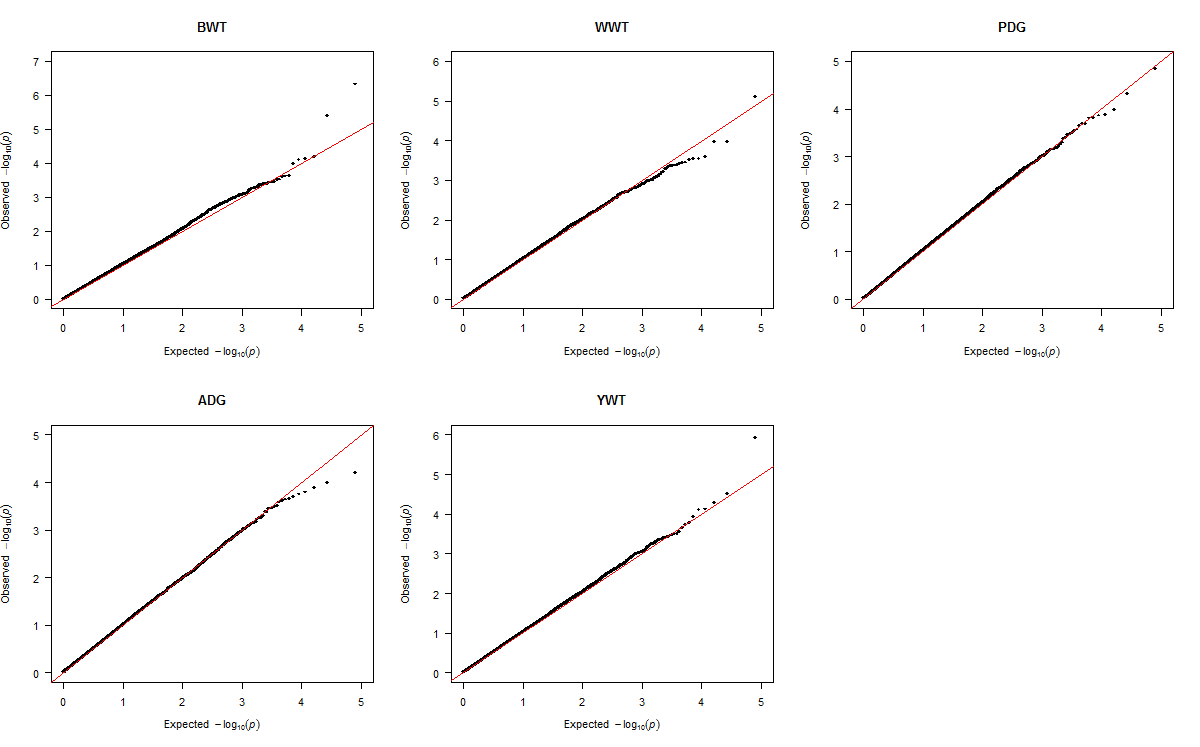 |
| --- |
| **Figure S9 Quantile-quantile (Q-Q) plot of *p*-values for dominance SNP association with growth traits in Crossbreds.** Crossbred group included Kinsella composite, Beefbooster TX composite (www.beefbooster.com) and two and more way crosses involving Angus, Hereford, Charolais, Gelbvieh, Simmental, Limousin, and Piedmontese breeds. The traits are birth weight (BWT); weaning weight (WWT); pre-weaning daily gain (PDG); average daily gain (ADG); and yearling weight (YWT). The red line denotes the expected distribution under the null hypothesis of no significant association. Deviations between the red line and black dots indicate how the test statistics of SNPs deviates from the null hypothesis |

| 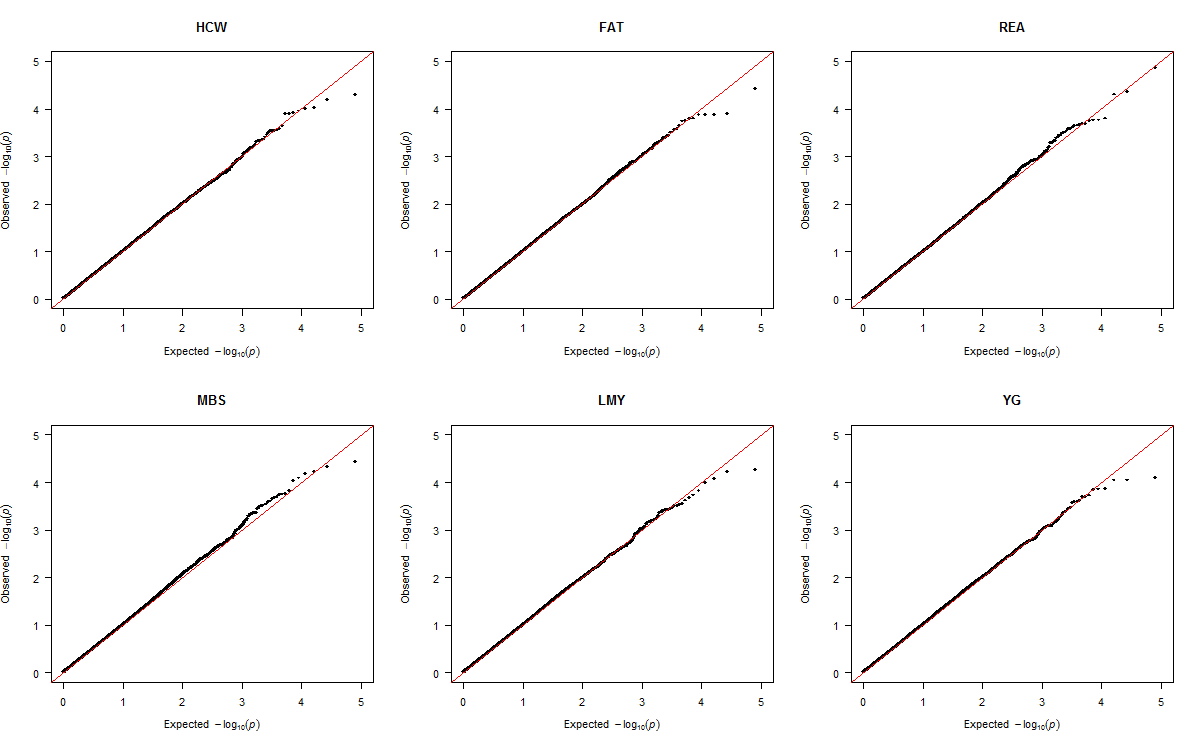 |
| --- |
| **Figure S10 Quantile-quantile (Q-Q) plot of *p*-values for dominance SNP association with carcass traits in Crossbreds.** Crossbred group included Kinsella composite, Beefbooster TX composite (www.beefbooster.com) and two and more way crosses involving Angus, Hereford, Charolais, Gelbvieh, Simmental, Limousin, and Piedmontese breeds. The traits are hot carcass weight (HCW); back fat thickness (FAT); rib eye area (REA); marbling score (MBS); lean meat yield (LMY) and yield grade (YG). The red line denotes the expected distribution under the null hypothesis of no significant association. Deviations between the red line and black dots indicate how the test statistics of SNPs deviates from the null hypothesis |

| 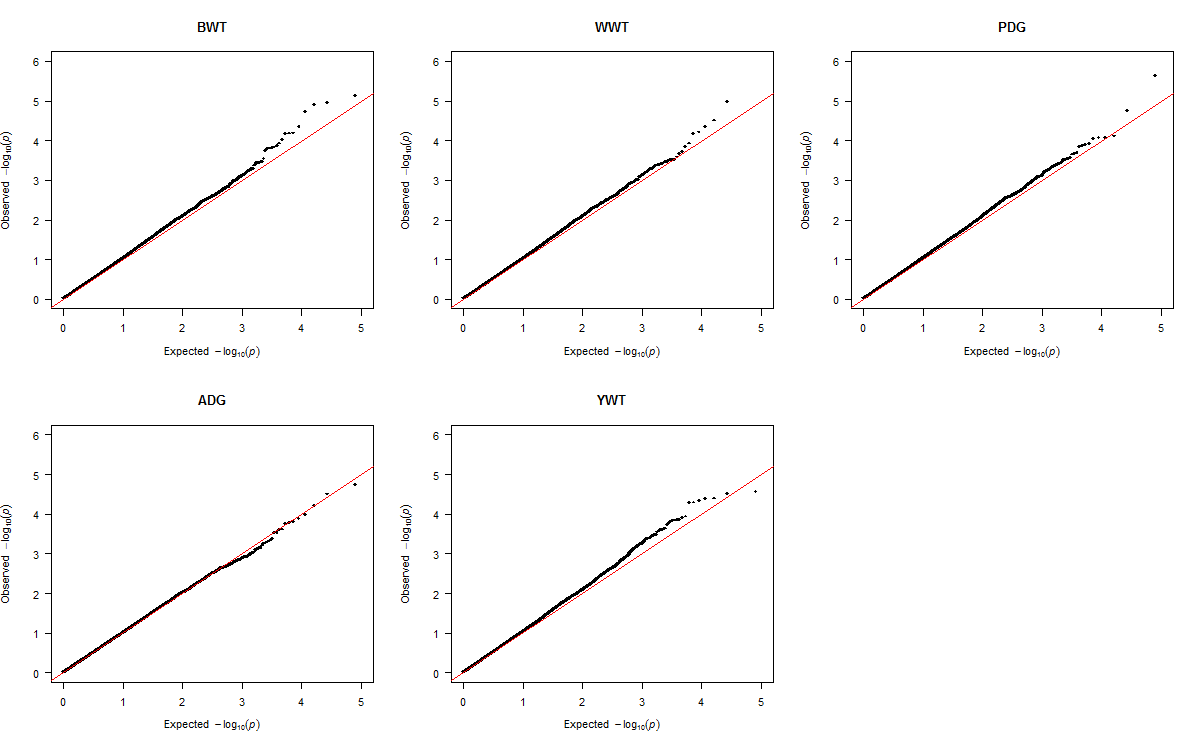 |
| --- |
| **Figure S11 Quantile-quantile (Q-Q) plot of *p*-values for dominance SNP association with growth traits in combined data.** The traits are birth weight (BWT); weaning weight (WWT); pre-weaning daily gain (PDG); average daily gain (ADG); and yearling weight (YWT). The red line denotes the expected distribution under the null hypothesis of no significant association. Deviations between the red line and black dots indicate how the test statistics of SNPs deviates from the null hypothesis |

| 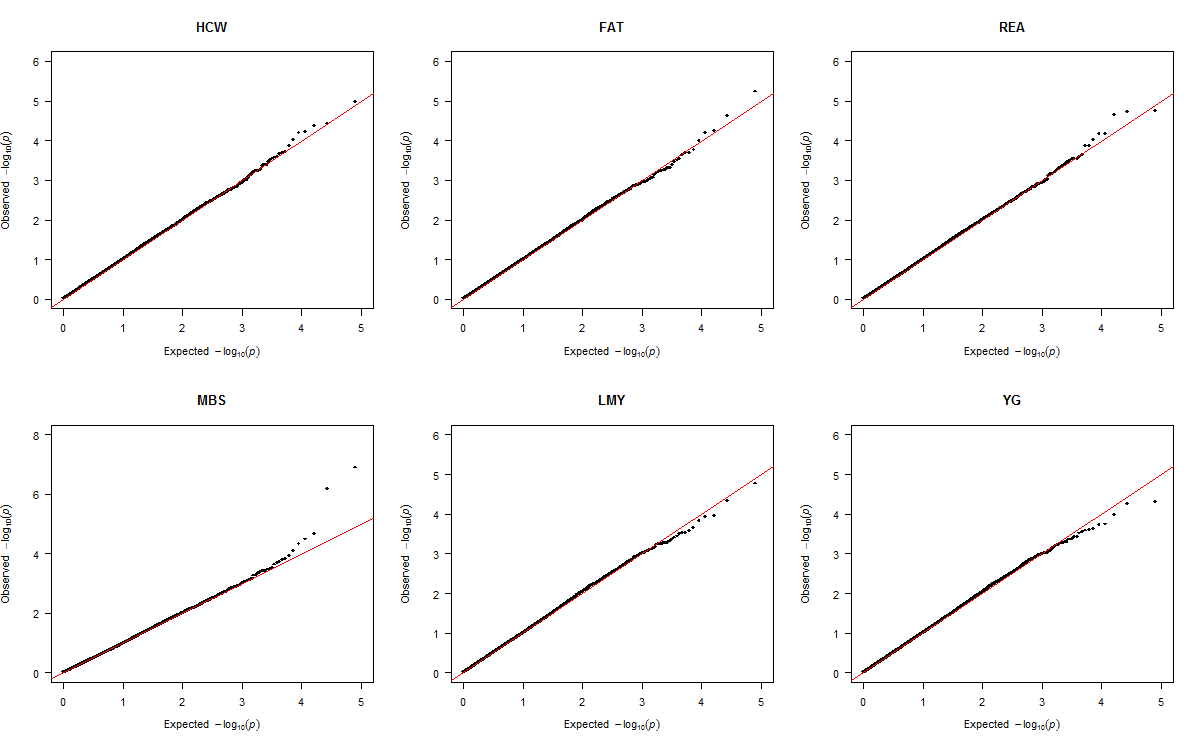 |
| --- |
| **Figure S12 Quantile-quantile (Q-Q) plot of *p*-values for dominance SNP association with carcass traits in combined data.** The traits are hot carcass weight (HCW); back fat thickness (FAT); rib eye area (REA); marbling score (MBS); lean meat yield (LMY) and yield grade (YG). The red line denotes the expected distribution under the null hypothesis of no significant association. Deviations between the red line and black dots indicate how the test statistics of SNPs deviates from the null hypothesis |

| 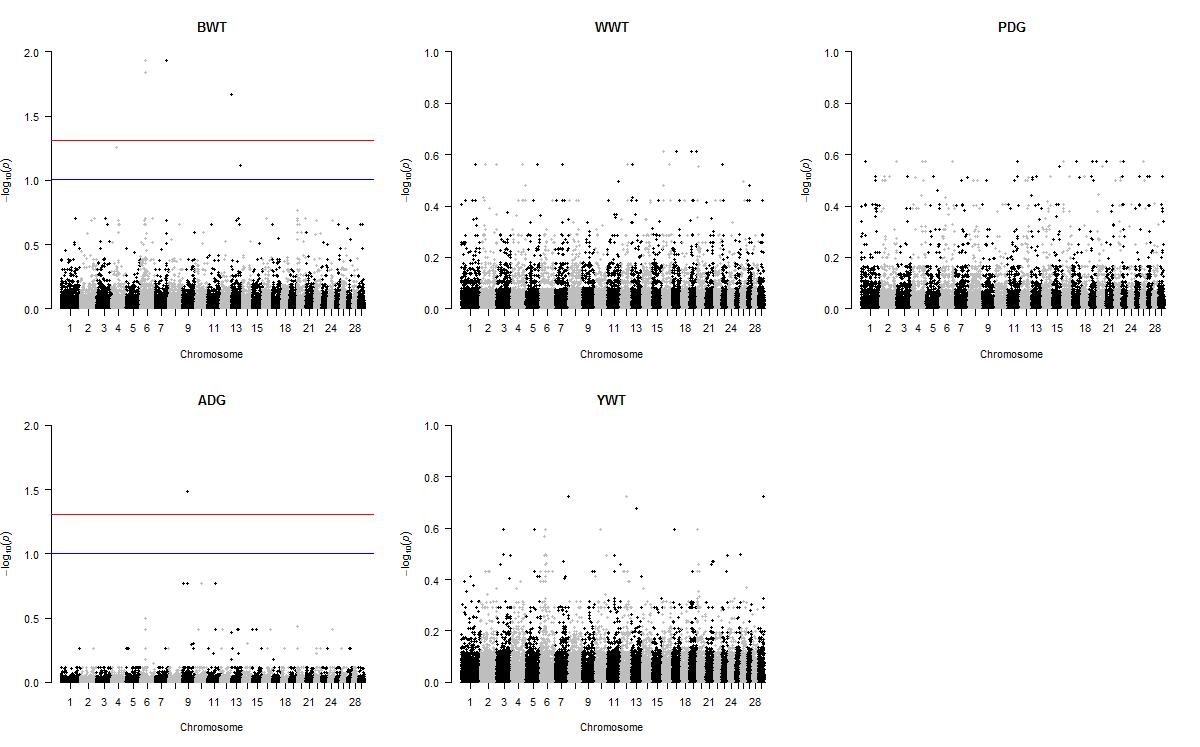 |
| --- |
| **Figure S13 Joint genome-wide association analysis for additive SNP effects on growth traits of Purebreds.** Purebred group included individuals with > 80% of Angus, Hereford and Charolais breed proportions, respectively. The traits are birth weight (BWT); weaning weight (WWT); pre-weaning daily gain (PDG); average daily gain (ADG); and yearling weight (YWT). Significant SNPs were determined by false discovery rate correction at 5% (red line) and 10% (blue line) |

| 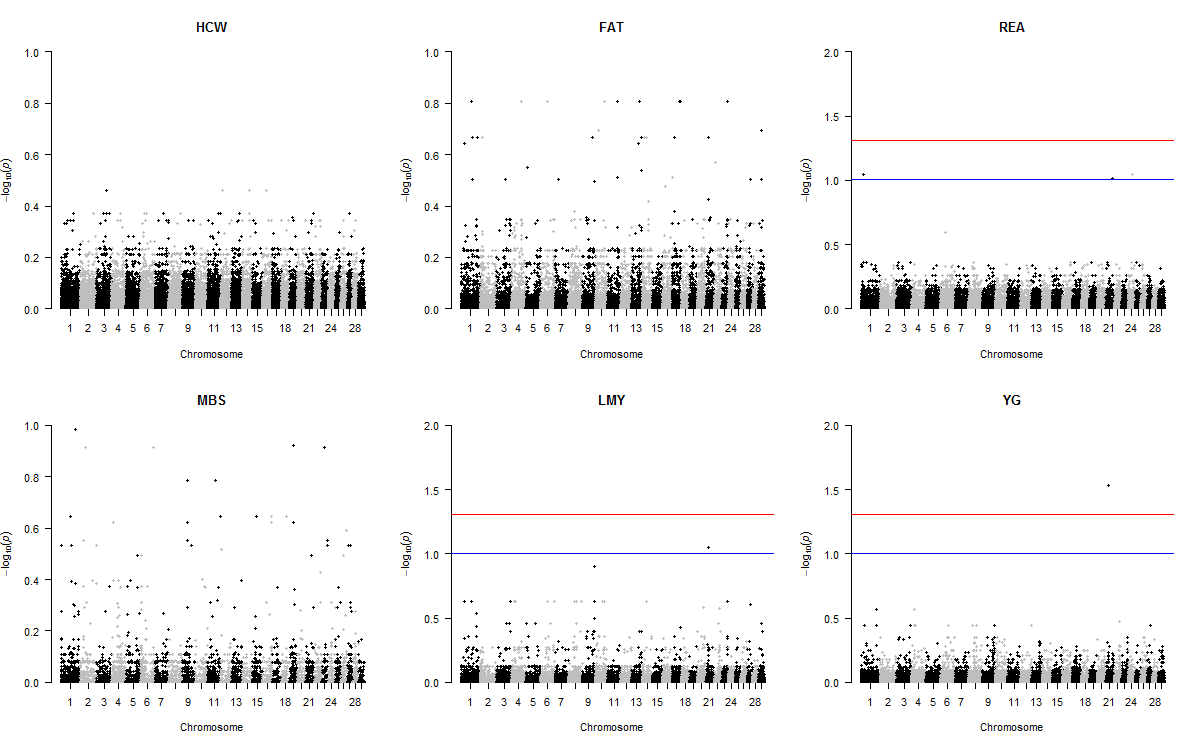 |
| --- |
| **Figure S14 Joint genome-wide association analysis for additive SNP effects on carcass traits of Purebreds.** Purebred group included individuals with > 80% of Angus, Hereford and Charolais breed proportions, respectively. The traits are hot carcass weight (HCW); back fat thickness (FAT); rib eye area (REA); marbling score (MBS); lean meat yield (LMY) and yield grade (YG). Significant SNPs were determined by false discovery rate correction at 5% (red line) and 10% (blue line) |

| 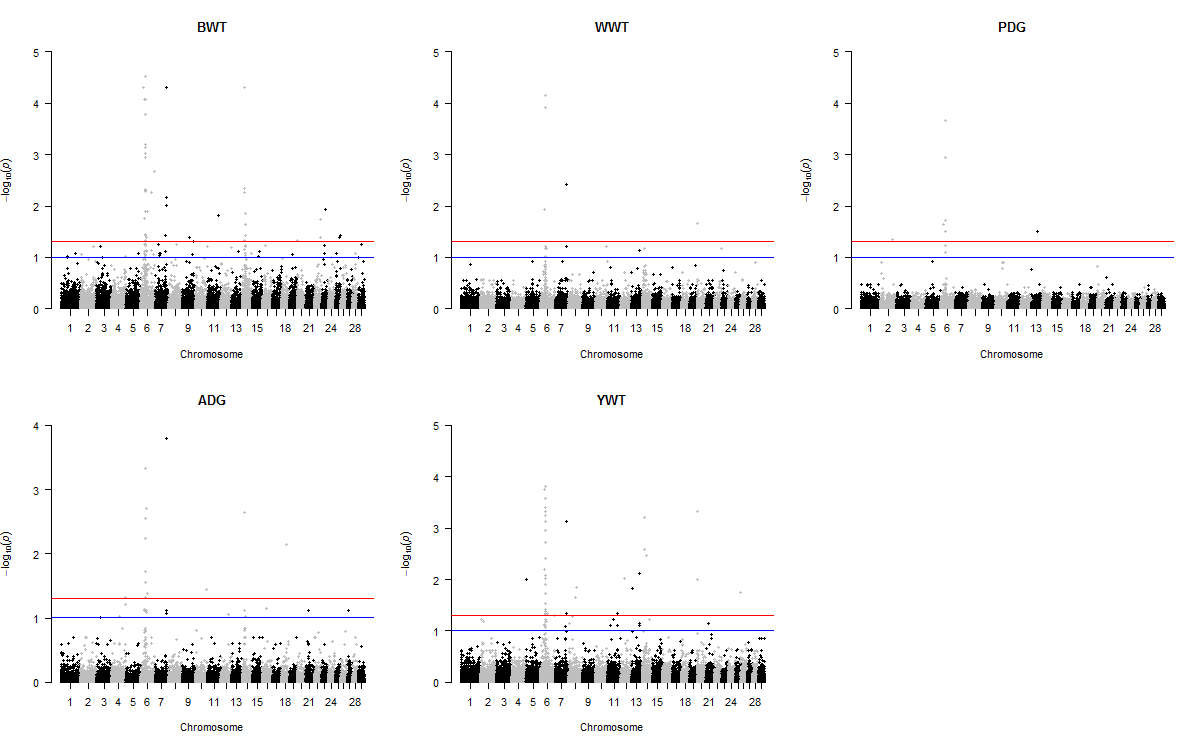 |
| --- |
| **Figure S15 Joint genome-wide association analysis for additive SNP effects on growth traits of Crossbreds.** Crossbred group included Kinsella composite, Beefbooster TX composite (www.beefbooster.com) and two and more way crosses involving Angus, Hereford, Charolais, Gelbvieh, Simmental, Limousin, and Piedmontese breeds. The traits are birth weight (BWT); weaning weight (WWT); pre-weaning daily gain (PDG); average daily gain (ADG); and yearling weight (YWT). Significant SNPs were determined by false discovery rate correction at 5% (red line) and 10% (blue line) |

| 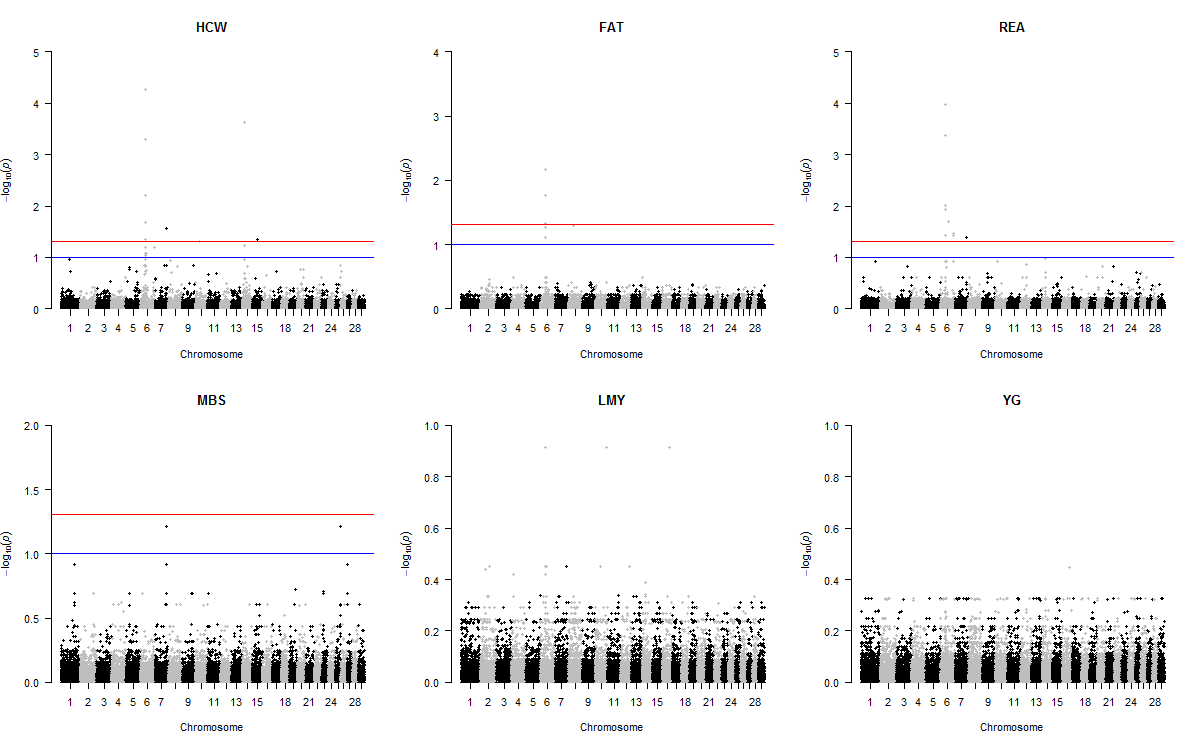 |
| --- |
| **Figure S16 Joint genome-wide association analysis for additive SNP effects on carcass traits of Crossbreds.** Crossbred group included Kinsella composite, Beefbooster TX composite (www.beefbooster.com) and two and more way crosses involving Angus, Hereford, Charolais, Gelbvieh, Simmental, Limousin, and Piedmontese breeds. The traits are hot carcass weight (HCW); back fat thickness (FAT); rib eye area (REA); marbling score (MBS); lean meat yield (LMY) and yield grade (YG). Significant SNPs were determined by false discovery rate correction at 5% (red line) and 10% (blue line) |

| 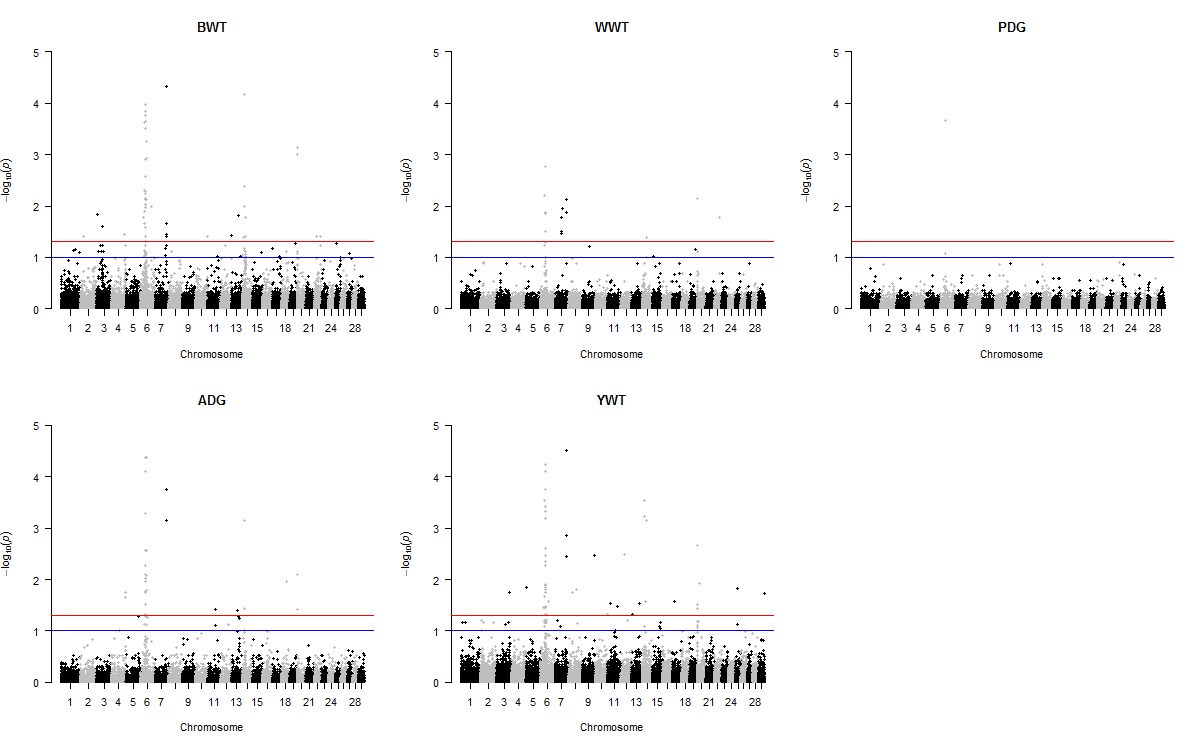 |
| --- |
| **Figure S17 Joint genome-wide association analysis for additive SNP effects on growth traits in combined data.** The traits are birth weight (BWT); weaning weight (WWT); pre-weaning daily gain (PDG); average daily gain (ADG); and yearling weight (YWT). Significant SNPs were determined by false discovery rate correction at 5% (red line) and 10% (blue line) |

| 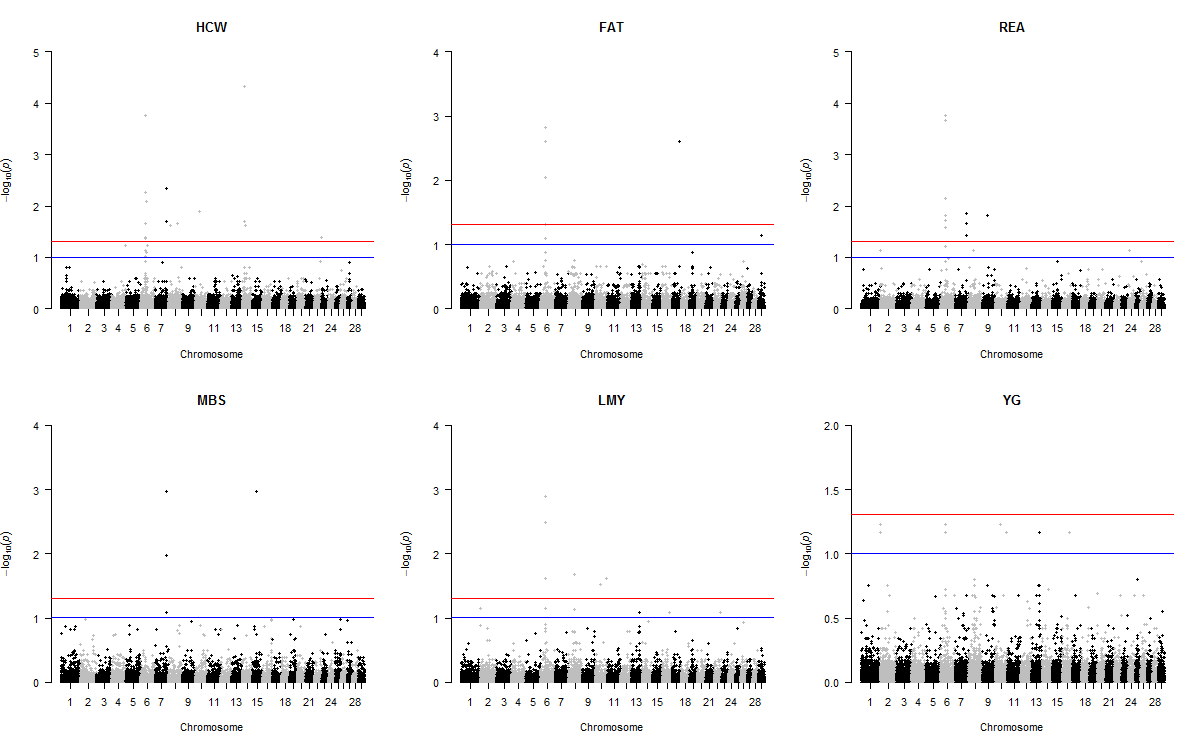 |
| --- |
| **Figure S18 Joint genome-wide association analysis for additive SNP effects on carcass traits in combined data.** The traits are hot carcass weight (HCW); back fat thickness (FAT); rib eye area (REA); marbling score (MBS); lean meat yield (LMY) and yield grade (YG). Significant SNPs were determined by false discovery rate correction at 5% (red line) and 10% (blue line) |

| 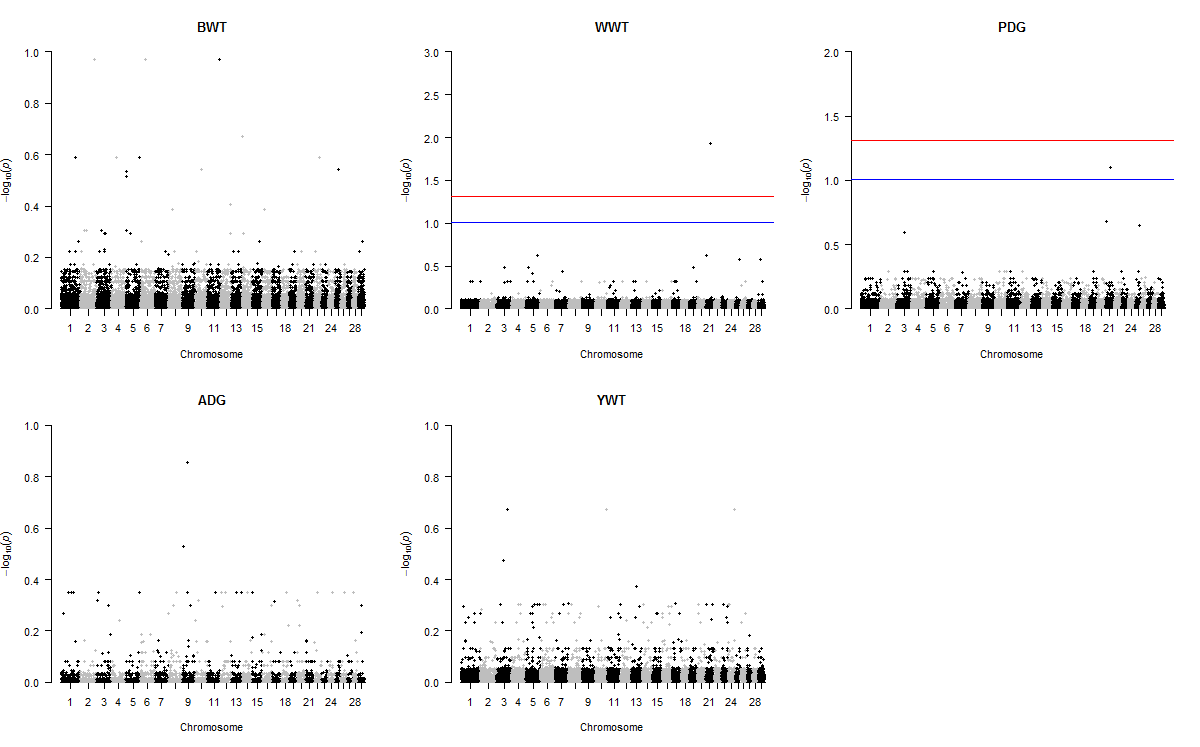 |
| --- |
| **Figure S19 Joint genome-wide association analysis for dominance SNP effects on growth traits of Purebreds.** Purebred group included individuals with > 80% of Angus, Hereford and Charolais breed proportions, respectively. The traits are birth weight (BWT); weaning weight (WWT); pre-weaning daily gain (PDG); average daily gain (ADG); and yearling weight (YWT). Significant SNPs were determined by false discovery rate correction at 5% (red line) and 10% (blue line) |

| 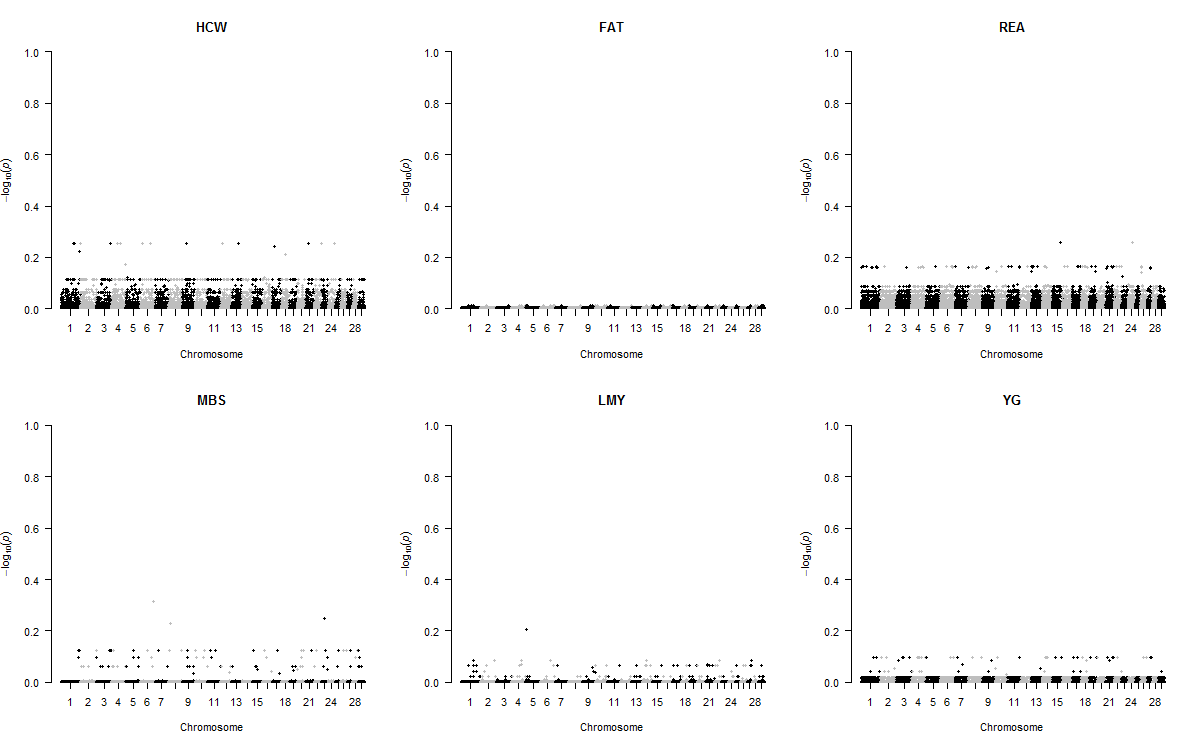 |
| --- |
| **Figure S20 Joint genome-wide association analysis for dominance SNP effects on carcass traits of Purebreds.** Purebred group included individuals with > 80% of Angus, Hereford and Charolais breed proportions, respectively. The traits are hot carcass weight (HCW); back fat thickness (FAT); rib eye area (REA); marbling score (MBS); lean meat yield (LMY) and yield grade (YG). Significant SNPs were determined by false discovery rate correction at 5% (red line) and 10% (blue line) |

| 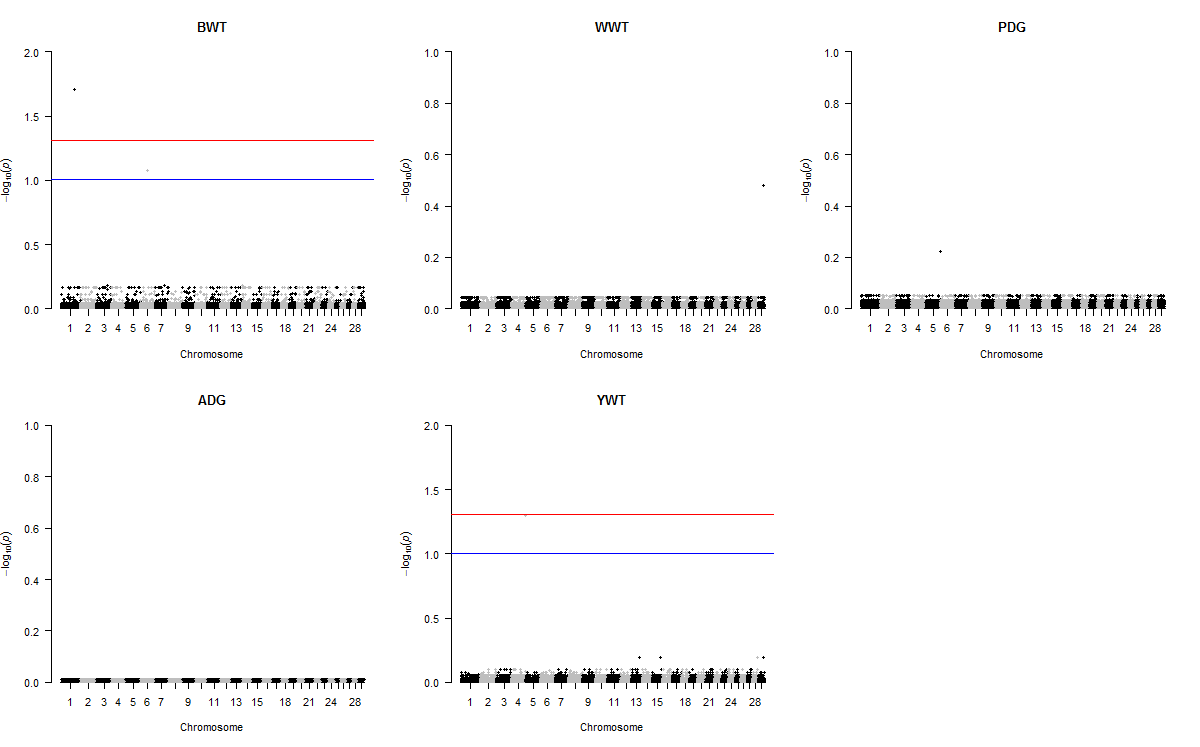 |
| --- |
| **Figure S21 Joint genome-wide association analysis for dominance SNP effects on growth traits of Crossbreds.** Crossbred group included Kinsella composite, Beefbooster TX composite (www.beefbooster.com) and two and more way crosses involving Angus, Hereford, Charolais, Gelbvieh, Simmental, Limousin, and Piedmontese breeds. The traits are birth weight (BWT); weaning weight (WWT); pre-weaning daily gain (PDG); average daily gain (ADG); and yearling weight (YWT). Significant SNPs were determined by false discovery rate correction at 5% (red line) and 10% (blue line) |

| 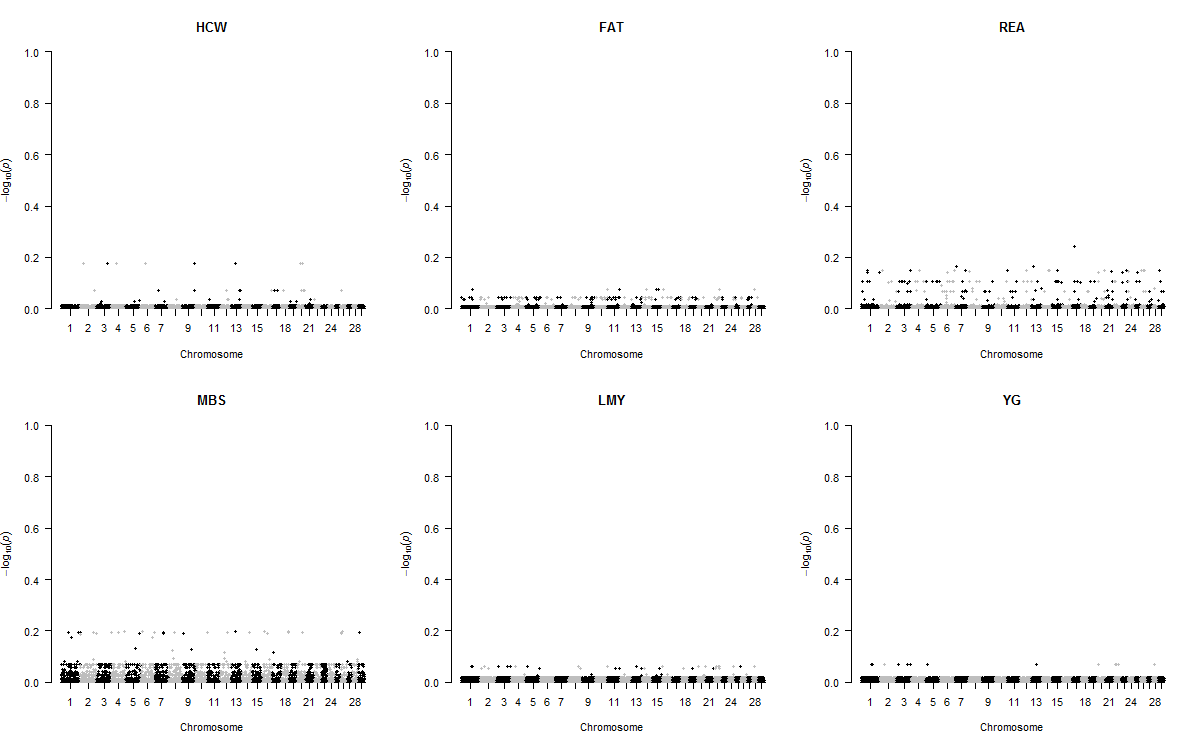 |
| --- |
| **Figure S22 Joint genome-wide association analysis for dominance SNP effects on carcass traits of Crossbreds**. Crossbred group included Kinsella composite, Beefbooster TX composite (www.beefbooster.com) and two and more way crosses involving Angus, Hereford, Charolais, Gelbvieh, Simmental, Limousin, and Piedmontese breeds. The traits are hot carcass weight (HCW); back fat thickness (FAT); rib eye area (REA); marbling score (MBS); lean meat yield (LMY) and yield grade (YG). Significant SNPs were determined by false discovery rate correction at 5% (red line) and 10% (blue line) |

| 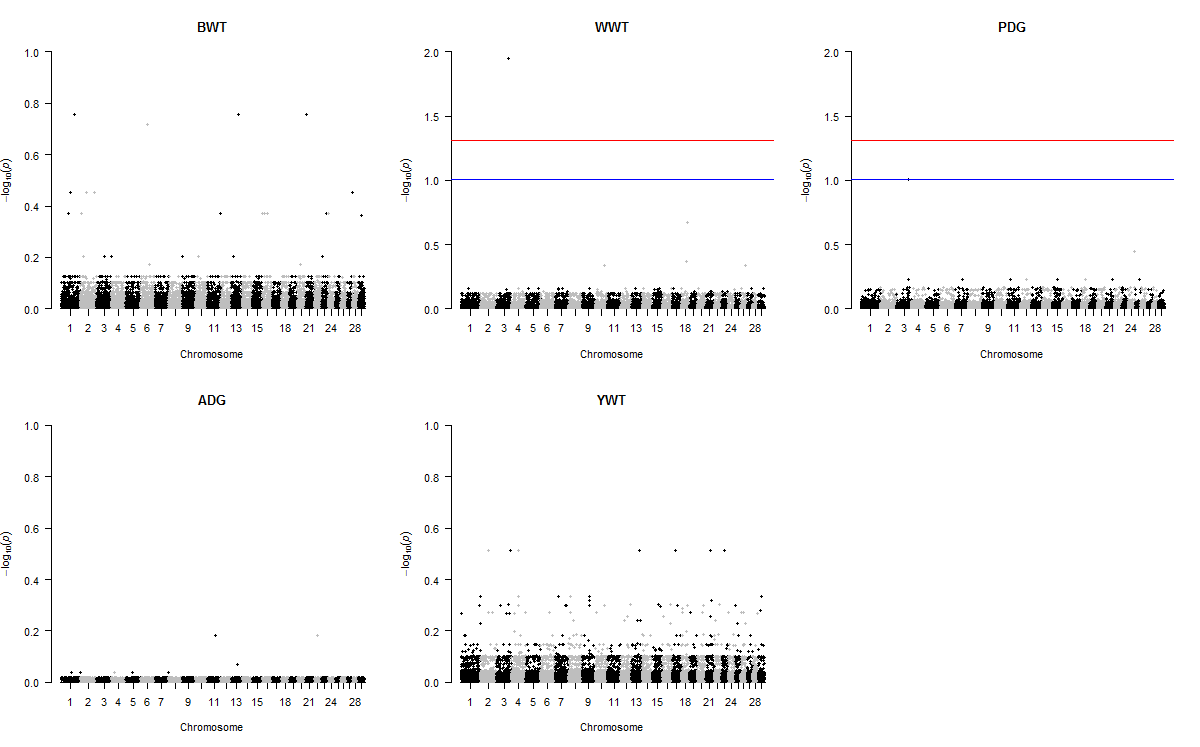 |
| --- |
| **Figure S23 Joint genome-wide association analysis for dominance SNP effects on growth traits in combined data.** The traits are birth weight (BWT); weaning weight (WWT); pre-weaning daily gain (PDG); average daily gain (ADG); and yearling weight (YWT). Significant SNPs were determined by false discovery rate correction at 5% (red line) and 10% (blue line) |

| 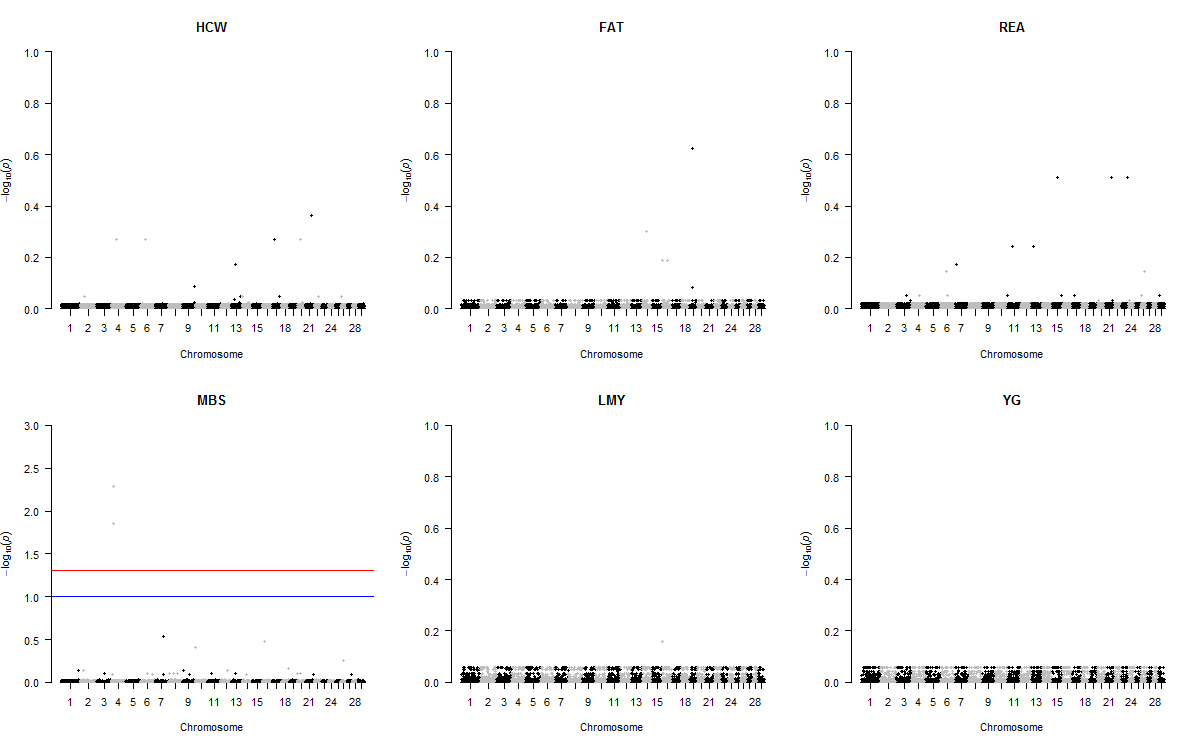 |
| --- |
| **Figure S24 Joint genome-wide association analysis for dominance SNP effects on carcass traits in combined data.** The traits are hot carcass weight (HCW); back fat thickness (FAT); rib eye area (REA); marbling score (MBS); lean meat yield (LMY) and yield grade (YG). Significant SNPs were determined by false discovery rate correction at 5% (red line) and 10% (blue line) |
